# Supplementary material for: Exercise-induced response of proteinogenic and non-proteinogenic plasma free amino acids is sport-specific: A comparison of sprint and endurance athletes
Source: PLoS One. 2024 Aug 30;19(8):e0309529. doi: 10.1371/journal.pone.0309529 (PMC11364291; doi:10.1371/journal.pone.0309529)
Supplement: S1 Table — (PDF) [file pone.0309529.s001.PDF]

S1 Table Multiple reaction monitoring (MRM) transitions for 42 amino acids being analyzed using LC-MS/MS-base methodology and their corresponding internal standards with the applied collision energy values and limit of quantitation values achieved by the methodology used

No	Amino acid	Abbrev.	MRM transition (Q1 → Q3)		Collision energy (eV)	Limit of quantitation (μmol·L^{-1})
			Analyte	Internal std.		
1	1-methylhistidine	1MHis	318.2 → 121.1	310.2 → 113.1	30	0.2
2	3-methylhistidine	3MHis	318.2 → 121.1	310.2 → 113.1	30	0.2
3	alanine	Ala	238.2 → 121.1	230.2 → 113.1	30	0.2
4	anserine	Ans	389.2 → 121.1	381.2 → 113.1	30	0.5
5	arginine	Arg	323.2 → 121.1	315.2 → 113.1	30	0.5
6	argininosuccinic acid	Asa	439.2 → 121.1	431.2 → 113.1	50	1.0
7	asparagine	Asn	281.2 → 121.1	273.2 → 113.1	30	0.5
8	aspartic acid	Asp	282.1 → 121.1	274.1 → 113.1	30	0.1
9	carnosine	Car	375.2 → 121.1	367.2 → 113.1	30	0.5
10	citrulline	Cit	324.2 → 121.1	316.2 → 113.1	30	0.5
11	cystathionine	Cth	519.3 → 121.1	503.3 → 113.1	50	0.5
12	cystine	Cys	537.2 → 121.1	521.2 → 113.1	50	1.0
13	ethanolamine	EtN	210.2 → 121.1	202.2 → 113.1	30	0.5
14	glutamic acid	Glu	296.2 → 121.1	288.2 → 113.1	30	0.5
15	glutamine	Gln	295.2 → 121.1	287.2 → 113.1	30	0.5
16	glycine	Gly	224.1 → 121.1	216.1 → 113.1	30	1.0
17	histidine	His	304.2 → 121.1	296.2 → 113.1	30	0.5
18	homocitrulline	Hcit	338.2 → 121.1	330.2 → 113.1	30	0.2
19	homocystine	Hcy	565.3 → 121.1	549.3 → 113.1	50	0.5
20	hydroxyproline	Hyp	280.1 → 121.1	272.1 → 113.1	30	0.2
21	isoleucine	Ile	280.2 → 121.1	272.2 → 113.1	30	0.5
22	leucine	Leu	280.2 → 121.1	272.2 → 113.1	30	0.5
23	lysine	Lys	443.3 → 121.1	427.3 → 113.1	50	0.5
24	methionine	Met	298.2 → 121.1	290.2 → 113.1	30	0.1
25	ornithine	Orn	429.3 → 121.1	413.3 → 113.1	50	0.5
26	phenylalanine	Phe	314.2 → 121.1	306.2 → 113.1	30	0.2
27	phosphoethanolamine	PEtN	290.1 → 121.1	282.1 → 113.1	30	0.5
28	phosphoserine	PSer	334.1 → 121.1	326.1 → 113.1	30	0.5
29	proline	Pro	264.2 → 121.1	256.2 → 113.1	30	0.1
30	sarcosine	Sar	238.2 → 121.1	230.2 → 113.1	30	0.2
31	serine	Ser	254.2 → 121.1	246.2 → 113.1	30	0.5
32	taurine	Tau	274.1 → 121.1	266.1 → 113.1	30	0.5
33	threonine	Thr	268.2 → 121.1	260.2 → 113.1	30	0.2
34	tryptophan	Trp	353.2 → 121.1	345.2 → 113.1	30	0.1
35	tyrosine	Tyr	330.2 → 121.1	322.2 → 113.1	30	0.5
36	valine	Val	266.2 → 121.1	258.2 → 113.1	30	0.2
37	α-aminoadipic acid	Aad	310.2 → 121.1	302.2 → 113.1	30	0.2
38	α-amino-n-butyric acid	Abu	252.2 → 121.1	244.2 → 113.1	30	0.2
39	β-alanine	bAla	238.2 → 121.1	230.2 → 113.1	30	0.5
40	β-aminoisobutyric acid	bAib	252.2 → 121.1	244.2 → 113.1	30	0.2
41	γ-amino-n-butyric acid	GABA	252.2 → 121.1	244.2 → 113.1	30	0.05
42	δ-hydroxylysine	Hyl	459.3 → 121.1	443.3 → 113.1	50	0.5
