# Supplementary material for: Exercise-induced response of proteinogenic and non-proteinogenic plasma free amino acids is sport-specific: A comparison of sprint and endurance athletes
Source: PLoS One. 2024 Aug 30;19(8):e0309529. doi: 10.1371/journal.pone.0309529 (PMC11364291; doi:10.1371/journal.pone.0309529)
Supplement: S2 Table — (PDF) [file pone.0309529.s002.PDF]

S2 Table Mean values ± standard deviations of the absolute and SMM-adjusted concentrations of individual PFAAs or their classes in endurance and sprint-trained athletes before exercise, during the progressive test until exhaustion, and post-exercise recovery.

Legend: EN endurance athletes, SP sprint-trained athletes

**Absolute** (μmol·L^{-1})		**Rest**	**Exercise: Speed** (km·h^{-1})					**Exhaust.**	**Recovery: Time** (min)				
			10	*12*	*14*	*16*	*18*		*5*	*10*	*15*	*20*	*30*
**His**	EN	79.0±13.8	68.6±11.3	67.2±8.2	65.0±8.5	67.2±8.8	65.2±7.8	66.5±9.2	63.7±8.5	67.8±9.8	74.0±12.2	74.4±12.5	76.1±7.3
	SP	75.1±9.6	69.0±9.0	68.8±9.2	69.9±9.1	67.7±7.1	--	69.9±6.2	68.5±6.5	73.3±6.6	77.6±8.4	77.1±8.9	75.9±7.3
**Ile**	EN	76.4±29.4	60.2±14.2	57.7±12.8	55.8±11.3	56.0±10.9	52.9±9.1	52.8±9.1	53.7±8.0	53.4±9.0	54.8±10.3	52.5±10.6	76.4±29.4
	SP	83.4±42.7	71.7±31.5	71.6±29.7	71.2±26.8	68.3±24.6	--	69.0±23.7	72.5±25.0	68.9±20.8	68.6±20.1	69.3±26.3	83.4±42.7
**Leu**	EN	137±45	110±24	105±20	100±18	103±19	97±12	96±12	100±11	98±15	103±17	100±16	99±14
	SP	148±68	128±52	130±53	128±47	122±42	--	123±40	132±44	126±37	128±37	130±46	129±52
**Lys**	EN	223±47	194±31	188±27	184±26	189±27	183±28	185±26	174±28	169±28	172±33	170±34	177±30
	SP	244±81	211±59	214±61	214±51	211±53	--	214±51	205±49	198±42	198±38	194±34	202±39
**Met**	EN	32.8±7.3	27.2±4.7	26.8±5.0	25.7±4.3	26.5±4.3	25.5±4.4	25.3±4.3	25.2±4.3	25.8±4.5	26.8±5.1	26.5±5.6	27.2±4.4
	SP	37.1±13.7	31.7±9.3	31.7±9.3	31.8±8.2	30.2±7.8	--	31.0±7.0	30.9±7.3	30.3±6.3	31.4±6.0	31.2±6.1	31.2±6.4
**Phe**	EN	71.6±11.3	62.3±8.1	60.7±7.8	58.4±4.9	59.5±5.3	57.8±4.4	57.2±4.5	56.0±4.8	57.1±4.5	59.9±6.1	59.4±6.2	61.8±4.1
	SP	68.9±15.6	60.5±10.3	60.5±10.3	61.5±10.8	59.2±9.2	--	60.4±10.4	60.4±9.0	60.5±9.0	62.5±10.6	62.6±9.4	63.4±9.2
**Thr**	EN	136±32	117±23	116±23	111±21	111±22	104±23	97±23	98±23	102±23	109±26	110±27	117±24
	SP	146±36	127±25	124±27	123±22	110±21	--	112±21	115±22	121±22	126±22	125±18	127±23
**Trp**	EN	50.6±8.0	44.4±6.6	43.1±5.6	42.2±5.4	42.1±5.6	38.4±4.2	33.5±4.8	32.0±5.2	35.0±5.3	38.3±6.1	40.4±7.0	43.5±5.8
	SP	47.4±11.7	40.6±8.4	40.0±8.7	38.9±7.9	34.4±6.8	--	33.0±6.8	31.8±6.9	34.1±6.4	37.3±7.4	39.4±6.4	42.0±6.7
**Val**	EN	303±74	259±45	249±40	247±43	251±42	239±33	231±33	234±31	241±34	248±40	249±43	256±37
	SP	316±101	285±81	284±79	285±78	266±70	--	268±68	281±73	279±64	292±60	295±66	296±75
**Arg**	EN	71.8±19.1	69.6±17.5	62.9±11.2	61.0±12.1	62.7±12.6	62.6±11.5	62.2±10.5	58.3±9.4	55.8±8.3	59.9±8.7	57.9±8.6	60.9±13.6
	SP	86.8±17.7	77.1±14.0	79.1±12.9	80.1±12.5	75.9±11.0	--	79.6±13.0	74.7±10.9	73.5±8.6	72.2±11.1	70.5±8.7	75.5±14.8
**Cyss**	EN	44.8±9.9	38.5±6.3	38.8±7.6	41.2±6.5	42.1±7.5	39.5±6.9	39.4±5.2	40.5±5.2	44.2±7.6	45.5±7.3	46.7±5.9	47.4±6.5
	SP	30.6±5.9	28.3±5.2	27.4±4.5	30.2±7.8	27.8±6.7	--	25.8±4.8	28.1±4.3	30.6±6.2	30.8±4.1	32.2±7.0	30.2±7.5
**Gln**	EN	617±90	569±88	544±74	526±60	548±70	542±61	542±72	496±67	512±76	552±95	553±88	599±95
	SP	623±53	589±62	599±49	603±55	591±44	--	611±36	578±44	573±48	594±57	616±66	625±43
**Gly**	EN	183±20	170±21	164±18	162±20	170±19	162±22	160±20	147±20	144±20	149±23	148±26	163±25
	SP	203±40	189±41	188±39	190±38	180±38	--	183±34	177±41	172±36	175±44	175±43	185±42
**Pro**	EN	229±61	203±46	194±47	185±41	186±44	174±41	167±42	166±40	175±42	187±49	196±49	209±50
	SP	212±43	179±20	183±24	184±22	168±21	--	169±20	173±27	185±24	195±27	198±19	201±19
**Tyr**	EN	85.8±29.3	72.3±21.4	71.0±20.6	69.7±19.8	70.9±19.6	67.8±16.3	67.8±16.1	67.3±14.5	68.4±15.8	71.2±16.4	70.9±19.6	73.5±17.7
	SP	84.5±35.1	73.5±25.9	72.9±23.9	74.7±25.9	71.0±24.4	--	72.5±23.4	72.7±21.6	71.6±20.8	74.4±20.8	73.2±17.6	74.2±21.5
**Ala**	EN	408±78	375±54	393±47	398±50	429±47	462±48	506±47	496±50	501±42	506±50	500±55	519±49
	SP	358±96	334±101	381±80	405±79	437±97	--	456±77	445±71	464±71	461±78	469±60	474±73
**Asn**	EN	62.1±9.0	53.6±6.4	52.8±8.3	51.5±6.3	52.0±6.8	48.1±7.1	47.1±5.7	45.8±7.3	47.6±6.3	50.3±6.8	50.1±7.6	53.8±7.3
	SP	76.2±24.1	65.4±14.8	63.9±14.7	64.8±14.3	59.6±11.8	--	58.9±12.4	59.9±15.1	61.4±13.6	63.7±13.3	65.0±12.6	66.8±15.8
**Asp**	EN	2.30±1.47	1.74±0.60	1.86±0.74	2.31±1.12	2.03±0.75	2.04±0.64	2.15±1.07	3.11±1.07	2.84±1.06	3.06±1.45	2.48±0.96	2.64±1.63
	SP	2.12±1.18	1.98±1.01	1.88±0.83	2.27±1.28	2.19±1.49	--	2.23±1.22	4.09±2.35	4.17±1.80	4.31±3.11	3.13±0.91	2.61±1.26
**Glu**	EN	18.8±9.2	11.1±4.4	13.1±4.7	14.3±4.6	14.4±6.3	11.7±5.4	12.6±5.6	22.8±5.9	28.1±8.2	29.9±9.8	32.9±10.1	30.5±10.9
	SP	14.3±5.8	11.9±3.7	12.4±3.8	10.4±1.9	9.0±1.6	--	10.3±3.3	22.3±3.5	26.8±3.5	32.6±7.5	33.9±8.4	30.6±10.3
**Ser**	EN	90.9±15.8	79.8±15.0	78.6±12.5	76.7±9.6	75.2±8.0	69.2±8.9	66.1±11.5	68.5±10.5	70.9±10.0	77.2±16.9	75.2±14.1	80.0±11.4
	SP	85.5±22.8	73.7±17.4	74.1±18.1	75.0±16.3	67.2±18.8	--	67.3±15.9	78.0±25.0	76.1±21.7	82.8±28.4	78.9±17.7	82.0±19.5

		Rest	Exercise: Speed (km·h^{-1})					Exhaust.	Recovery: Time (min)				
			10	*12*	*14*	*16*	*18*		*5*	*10*	*15*	*20*	*30*
**1Mhis**	EN	14.1±9.7	12.0±7.6	11.9±7.7	11.3±7.5	11.8±7.7	11.4±7.5	11.0±7.0	10.5±6.5	10.7±6.8	11.4±7.2	11.4±7.2	12.0±7.9
	SP	9.6±8.8	8.8±8.6	8.7±8.9	9.2±9.9	7.7±6.4	--	7.8±6.7	8.4±8.1	8.4±8.3	8.8±8.5	8.9±8.7	8.9±8.4
**3Mhis**	EN	5.65±1.20	5.10±1.09	4.95±1.04	4.90±1.04	5.03±0.97	4.93±1.16	5.08±1.06	4.95±1.17	4.99±1.06	5.34±1.31	5.43±1.28	5.67±1.09
	SP	6.46±1.64	5.99±1.38	5.99±1.49	6.10±1.40	5.87±1.33	--	6.11±1.45	6.20±1.42	6.35±1.48	6.68±1.47	6.68±1.45	6.66±1.48
**Aad**	EN	1.11±0.32	0.96±0.28	1.00±0.26	1.01±0.29	0.99±0.29	0.99±0.27	1.11±0.25	1.16±0.23	1.16±0.18	1.15±0.19	1.13±0.19	1.08±0.22
	SP	1.81±1.33	1.54±1.15	1.66±1.31	1.62±1.21	1.69±1.38	--	1.78±1.35	1.92±1.55	1.83±1.33	1.75±1.06	1.62±0.95	1.50±0.97
**Abu**	EN	22.6±7.5	20.0±6.3	19.4±5.9	18.7±5.4	18.7±5.2	17.2±5.1	15.6±4.5	16.4±4.5	17.6±4.8	18.5±5.4	18.8±5.1	19.6±5.6
	SP	22.0±7.4	19.5±6.3	19.5±6.2	18.9±6.7	16.5±5.0	--	16.8±4.8	18.2±5.9	18.7±5.9	19.9±5.6	20.1±5.6	20.3±5.5
**bAib**	EN	1.95±0.67	1.92±0.69	1.86±0.72	1.90±0.64	2.00±0.79	2.04±0.62	2.10±0.80	1.99±0.69	1.95±0.73	1.93±0.61	2.06±0.60	1.91±0.62
	SP	2.42±0.78	2.37±0.79	2.38±0.84	2.66±0.90	2.50±0.89	--	2.66±0.94	2.67±0.97	2.66±0.90	2.49±0.98	2.46±0.84	2.54±0.95
**bAla**	EN	38.3±9.3	40.4±6.4	38.5±5.1	40.4±5.8	38.1±6.6	39.3±5.7	36.2±6.4	38.5±6.0	39.9±7.9	40.1±8.2	40.1±9.6	43.0±9.6
	SP	41.1±8.6	37.1±15.3	34.4±12.6	39.7±9.8	28.1±11.9	--	32.1±11.8	37.5±9.8	40.2±10.4	42.1±7.3	40.6±12.5	33.1±11.7
**Cit**	EN	33.9±19.1	30.9±16.2	30.3±13.5	29.4±12.9	29.8±13.3	27.9±10.7	27.5±9.6	26.2±8.6	25.5±9.0	26.4±7.0	25.5±8.2	25.7±7.5
	SP	29.2±3.9	27.6±3.2	27.8±3.9	27.2±3.3	25.8±3.2	--	26.3±4.0	27.7±3.8	27.1±4.3	27.6±4.0	27.7±4.1	27.7±4.9
**EtN**	EN	9.5±1.6	9.9±1.4	10.1±1.6	10.3±1.3	11.2±1.5	12.0±1.7	12.6±1.6	11.4±1.6	11.5±1.6	11.2±1.5	10.7±1.5	10.5±1.3
	SP	9.4±0.9	10.0±1.3	10.7±1.4	11.4±1.8	11.5±1.7	--	12.2±1.5	11.7±1.4	11.2±1.2	10.9±1.3	10.3±2.0	9.8±1.4
**Hyp**	EN	9.6±4.9	8.7±4.4	8.5±4.3	8.0±4.0	8.0±4.0	7.4±3.9	6.8±3.7	7.0±4.1	7.4±4.5	8.1±4.9	8.1±4.6	8.4±3.9
	SP	12.7±4.6	10.8±3.8	10.8±3.9	10.3±3.5	9.1±3.1	--	9.1±3.2	9.9±3.9	10.1±3.6	10.9±4.0	11.1±4.3	11.3±4.4
**Orn**	EN	69.6±24.3	61.4±17.5	59.4±19.3	59.9±18.2	60.6±19.1	58.4±18.9	58.017.9±	53.0±17.2	52.8±17.0	53.1±17.1	52.6±19.0	54.1±14.5
	SP	55.6±10.3	51.2±11.1	48.0±9.0	49.2±9.1	48.1±10.6	--	49.8±10.1	49.0±13.5	47.8±13.3	48.5±12.1	47.2±11.2	49.5±12.5
**PEtN**	EN	2.09±1.01	1.52±0.67	1.66±0.71	1.76±0.61	2.02±0.76	1.74±0.47	2.07±0.53	2.11±0.69	2.03±0.92	1.98±0.99	1.69±0.71	1.52±0.78
	SP	1.44±0.38	1.21±0.24	1.32±0.22	1.48±0.24	1.63±0.26	--	1.71±0.25	1.89±0.26	1.79±0.29	1.68±0.27	1.48±0.33	1.44±0.25
**Sar**	EN	4.46±0.83	4.29±0.86	3.85±0.89	3.64±0.90	3.75±0.89	3.76±0.85	3.51±0.94	3.52±0.90	3.59±0.93	3.80±0.82	3.67±0.89	4.01±0.97
	SP	4.75±0.64	4.40±0.63	4.34±0.77	4.50±0.70	3.84±0.51	--	3.86±0.50	4.29±0.56	4.28±0.56	4.55±0.54	4.46±0.64	4.24±0.68
**Tau**	EN	42.9±10.9	37.4±4.5	39.1±4.9	40.5±6.9	44.1±9.0	40.1±5.0	42.5±7.0	43.1±8.0	44.0±10.6	45.0±13.0	41.9±9.0	40.2±8.0
	SP	33.8±6.5	30.8±5.3	31.3±5.4	32.4±6.3	32.7±6.5	--	34.2±6.4	35.8±6.7	35.5±6.0	35.6±5.7	33.9±5.4	33.0±5.4
**Prot.**	EN	2923±415	2587±259	2529±249	2477±213	2560±232	2501±198	2518±231	2448±242	2499±257	2615±323	2615±337	2750±259
	SP	2943±574	2646±405	2708±385	2743±336	2658±347	--	2715±323	2709±334	2728±237	2808±229	2839±197	2884±272
**Ess.**	EN	1109±225	944±132	914±113	890±108	907±109	861±88	844±91	837±100	849±107	885±136	882±142	910±108
	SP	1166±357	1024±256	1025±256	1023±229	968±210	--	980±203	997±214	991±177	1022±166	1024±174	1036±208
**n-Ess.**	EN	1814±210	1644±140	1616±146	1588±115	1653±135	1640±121	1674±149	1611±149	1650±157	1730±194	1733±203	1840±163
	SP	1777±242	1622±191	1684±159	1720±144	1690±161	--	1735±130	1712±158	1737±118	1786±141	1815±94	1848±112
**BCAA**	EN	516±147	430±82	412±71	404±70	411±69	388±50	380±50	388±47	392±54	405±66	402±67	408±58
	SP	547±210	485±162	486±159	484±150	456±133	--	460±129	486±139	473±119	489±113	494±134	494±150
**n-Prot.**	EN	256±46	234±32	231±34	232±30	236±34	227±28	224±26	220±27	223±31	228±34	223±25	228±20
	SP	230±24	211±24	207±25	215±32	195±19	--	204±21	215±20	216±24	221±23	217±25	210±22
**Total**	EN	3178±441	2822±273	2760±269	2709±227	2796±250	2729±213	2742±248	2668±263	2722±282	2843±350	2838±359	2978±270
	SP	3173±588	2858±407	2915±400	2958±353	2853±357	--	2919±330	2924±346	2944±251	3029±240	3056±201	3094±284

SMM-adjusted (µmol·L^{-1}·kg SMM^{-1})		Rest	**Exercise: Speed** (km·h^{-1})					Exhaust.	**Recovery: Time** (min)				
			10	*12*	*14*	*16*	*18*		*5*	*10*	*15*	*20*	*30*
**His**	EN	2.42±0.41	2.11±0.38	2.07±0.32	2.00±0.34	2.07±0.36	2.01±0.33	2.05±0.37	1.97±0.37	2.10±0.42	2.28±0.46	2.30±0.52	2.35±0.36
	SP	1.86±0.25	1.71±0.22	1.70±0.19	1.73±0.23	1.67±0.14	--	1.73±0.18	1.70±0.20	1.82±0.23	1.93±0.28	1.92±0.29	1.89±0.25
**Ile**	EN	2.31±0.80	1.83±0.35	1.75±0.30	1.70±0.27	1.71±0.27	1.61±0.22	1.61±0.26	1.65±0.24	1.64±0.28	1.68±0.33	1.61±0.33	1.61±0.28
	SP	2.04±0.94	1.75±0.66	1.75±0.62	1.75±0.59	1.68±0.51	--	1.690.50±	1.78±0.52	1.70±0.44	1.69±0.42	1.70±0.52	1.70±0.59
**Leu**	EN	4.16±1.23	3.37±0.63	3.20±0.54	3.07±0.50	3.16±0.53	2.96±0.37	2.94±0.45	3.08±0.44	3.02±0.59	3.16±0.65	3.07±0.59	3.04±0.53
	SP	3.64±1.53	3.15±1.11	3.18±1.10	3.15±1.04	2.99±0.89	--	3.03±0.86	3.25±0.94	3.11±0.81	3.17±0.79	3.19±0.93	3.15±1.04
**Lys**	EN	6.82±1.36	5.95±1.04	5.79±1.03	5.68±1.03	5.84±1.10	5.63±1.07	5.72±1.13	5.37±1.15	5.23±1.19	5.33±1.34	5.27±1.39	5.49±1.31
	SP	6.01±1.75	5.19±1.21	5.27±1.25	5.27±1.06	5.18±1.02	--	5.27±1.06	5.05±1.02	4.90±0.94	4.90±0.97	4.80±0.71	5.00±0.88
**Met**	EN	1.00±0.19	0.83±0.12	0.82±0.12	0.78±0.11	0.81±0.12	0.78±0.13	0.77±0.13	0.77±0.14	0.79±0.14	0.82±0.18	0.82±0.19	0.84±0.16
	SP	0.91±0.31	0.78±0.21	0.78±0.19	0.78±0.19	0.74±0.17	--	0.76±0.16	0.76±0.16	0.74±0.13	0.78±0.15	0.77±0.13	0.77±0.14
**Phe**	EN	2.19±0.32	1.91±0.25	1.86±0.23	1.79±0.18	1.83±0.21	1.77±0.18	1.76±0.22	1.72±0.22	1.76±0.23	1.84±0.27	1.83±0.29	1.90±0.25
	SP	1.71±0.43	1.51±0.31	1.51±0.32	1.53±0.35	1.47±0.30	--	1.51±0.35	1.50±0.30	1.51±0.32	1.56±0.38	1.56±0.33	1.58±0.32
**Thr**	EN	4.13±0.86	3.58±0.68	3.53±0.64	3.38±0.63	3.41±0.66	3.16±0.64	2.96±0.70	3.01±0.72	3.12±0.75	3.35±0.85	3.39±0.88	3.59±0.79
	SP	3.59±0.79	3.15±0.60	3.06±0.56	3.05±0.50	2.72±0.42	--	2.76±0.45	2.83±0.44	3.00±0.49	3.13±0.56	3.10±0.49	3.15±0.55
**Trp**	EN	1.55±0.24	1.36±0.22	1.32±0.19	1.30±0.20	1.29±0.20	1.18±0.14	1.03±0.18	0.98±0.17	1.08±0.20	1.18±0.24	1.24±0.26	1.34±0.24
	SP	1.17±0.27	1.01±0.19	0.99±0.20	0.97±0.21	0.85±0.18	--	0.82±0.17	0.78±0.15	0.85±0.16	0.93±0.21	0.98±0.17	1.04±0.19
**Val**	EN	9.22±1.92	7.92±1.18	7.61±0.99	7.56±1.14	7.68±1.07	7.30±0.86	7.09±0.95	7.17±0.91	7.39±1.04	7.60±1.34	7.65±1.41	7.85±1.17
	SP	7.79±2.30	7.02±1.78	7.01±1.68	7.03±1.82	6.58±1.57	--	6.63±1.55	6.94±1.61	6.90±1.50	7.25±1.50	7.29±1.45	7.30±1.64
**Arg**	EN	2.19±0.55	2.14±0.60	1.93±0.36	1.87±0.39	1.93±0.43	1.93±0.42	1.92±0.41	1.80±0.38	1.72±0.33	1.85±0.39	1.79±0.38	1.89±0.53
	SP	2.15±0.43	1.91±0.32	1.96±0.29	1.98±0.30	1.88±0.24	--	1.97±0.29	1.85±0.24	1.83±0.28	1.80±0.36	1.75±0.25	1.87±0.36
**Cyss**	EN	1.38±0.36	1.18±0.21	1.20±0.28	1.27±0.25	1.31±0.33	1.23±0.31	1.22±0.27	1.26±0.27	1.37±0.35	1.42±0.36	1.45±0.31	1.47±0.32
	SP	0.76±0.17	0.70±0.14	0.68±0.13	0.75±0.22	0.69±0.17	--	0.64±0.11	0.70±0.11	0.76±0.17	0.77±0.13	0.81±0.20	0.75±0.19
**Gln**	EN	19.0±3.6	17.6±4.0	16.8±3.1	16.3±3.0	17.0±3.3	16.7±3.0	16.8±3.3	15.3±3.0	15.9±3.5	17.1±4.0	17.1±4.0	18.6±4.4
	SP	15.5±1.6	14.6±1.3	14.9±1.6	15.0±1.8	14.7±1.1	--	15.2±1.8	14.4±1.8	14.3±2.0	14.8±2.2	15.3±2.3	15.6±2.2
**Gly**	EN	5.67±1.06	5.27±1.13	5.09±0.96	5.03±1.10	5.26±1.03	5.00±0.96	4.96±0.96	4.54±0.93	4.45±0.95	4.61±1.05	4.61±1.22	5.08±1.24
	SP	5.02±0.95	4.66±0.94	4.64±0.90	4.70±0.88	4.44±0.82	--	4.51±0.77	4.38±1.04	4.25±0.93	4.33±1.05	4.34±1.03	4.58±1.02
**Pro**	EN	6.96±1.58	6.20±1.31	5.92±1.31	5.63±1.16	5.68±1.25	5.32±1.27	5.11±1.29	5.09±1.22	5.36±1.28	5.71±1.57	6.01±1.56	6.42±1.65
	SP	5.27±1.11	4.45±0.62	4.55±0.65	4.56±0.63	4.17±0.63	--	4.20±0.66	4.30±0.72	4.61±0.79	4.86±0.87	4.94±0.79	4.99±0.61
**Tyr**	EN	2.60±0.80	2.20±0.57	2.16±0.55	2.12±0.53	2.16±0.53	2.07±0.43	2.07±0.45	2.06±0.41	2.09±0.46	2.18±0.49	2.17±0.53	2.25±0.52
	SP	2.10±0.91	1.83±0.67	1.81±0.62	1.86±0.68	1.77±0.64	--	1.81±0.62	1.81±0.56	1.78±0.56	1.86±0.58	1.82±0.54	1.85±0.58
**Ala**	EN	12.4±1.9	11.4±1.2	12.0±1.2	12.2±1.4	13.2±1.7	14.2±1.9	15.6±2.0	15.2±1.9	15.4±2.2	15.6±2.4	15.4±2.6	16.0±2.5
	SP	8.9±2.3	8.1±1.9	9.4±1.7	10.0±1.8	10.8±2.3	--	11.4±2.1	11.0±1.6	11.6±2.4	11.6±2.7	11.8±2.4	11.9±2.4
**Asn**	EN	1.90±0.27	1.64±0.17	1.61±0.23	1.58±0.21	1.60±0.23	1.47±0.19	1.45±0.21	1.41±0.25	1.47±0.25	1.55±0.27	1.55±0.31	1.66±0.32
	SP	1.88±0.57	1.61±0.34	1.58±0.33	1.60±0.35	1.48±0.30	--	1.46±0.32	1.48±0.34	1.52±0.32	1.58±0.34	1.61±0.32	1.65±0.39
**Asp**	EN	0.071±0.047	0.053±0.019	0.056±0.022	0.073±0.044	0.062±0.024	0.063±0.021	0.067±0.033	0.095±0.030	0.087±0.032	0.094±0.044	0.077±0.036	0.081±0.049
	SP	0.051±0.022	0.049±0.026	0.046±0.018	0.056±0.030	0.053±0.032	--	0.055±0.030	0.103±0.063	0.103±0.046	0.102±0.059	0.076±0.017	0.063±0.026
**Glu**	EN	0.58±0.31	0.34±0.14	0.40±0.15	0.44±0.17	0.45±0.20	0.36±0.17	0.40±0.20	0.71±0.23	0.87±0.27	0.92±0.33	1.01±0.33	0.93±0.32
	SP	0.35±0.13	0.29±0.09	0.31±0.09	0.26±0.05	0.22±0.04	--	0.26±0.08	0.55±0.09	0.67±0.12	0.81±0.20	0.84±0.21	0.76±0.26
**Ser**	EN	2.79±0.54	2.46±0.53	2.41±0.38	2.37±0.43	2.32±0.34	2.13±0.34	2.04±0.45	2.11±0.40	2.19±0.43	2.38±0.58	2.33±0.60	2.47±0.52
	SP	2.10±0.49	1.82±0.34	1.83±0.38	1.85±0.33	1.65±0.37	--	1.66±0.34	1.93±0.62	1.88±0.50	2.03±0.53	1.95±0.37	2.02±0.37
**1Mhis**	EN	0.43±0.28	0.36±0.22	0.36±0.23	0.34±0.22	0.36±0.23	0.35±0.22	0.34±0.21	0.32±0.19	0.33±0.21	0.35±0.22	0.35±0.22	0.37±0.23
	SP	0.25±0.24	0.23±0.23	0.23±0.24	0.24±0.26	0.20±0.18	--	0.20±0.19	0.22±0.22	0.22±0.22	0.23±0.23	0.23±0.24	0.23±0.23

		Rest	**Exercise: Speed** (km·h^{-1})					**Exhaust.**	**Recovery: Time** (min)				
			10	*12*	*14*	*16*	*18*		*5*	*10*	*15*	*20*	*30*
**3Mhis**	EN	0.174±0.041	0.157±0.038	0.153±0.036	0.151±0.035	0.155±0.035	0.152±0.038	0.157±0.036	0.153±0.041	0.154±0.037	0.165±0.045	0.168±0.045	0.175±0.039
	SP	0.159±0.036	0.148±0.031	0.148±0.033	0.151±0.031	0.145±0.029	--	0.151±0.032	0.153±0.032	0.157±0.034	0.166±0.036	0.166±0.035	0.165±0.035
**Aad**	EN	0.034±0.008	0.029±0.007	0.030±0.007	0.031±0.007	0.030±0.007	0.030±0.007	0.034±0.006	0.036±0.007	0.036±0.006	0.035±0.006	0.035±0.006	0.033±0.006
	SP	0.044±0.032	0.037±0.026	0.040±0.030	0.039±0.028	0.041±0.031	--	0.043±0.030	0.046±0.034	0.044±0.029	0.043±0.023	0.040±0.021	0.036±0.021
**Abu**	EN	0.69±0.21	0.61±0.19	0.59±0.18	0.57±0.16	0.57±0.16	0.52±0.15	0.48±0.14	0.50±0.14	0.54±0.15	0.57±0.18	0.58±0.16	0.60±0.18
	SP	0.55±0.20	0.49±0.18	0.49±0.18	0.48±0.19	0.42±0.15	--	0.42±0.14	0.46±0.16	0.47±0.16	0.50±0.17	0.50±0.16	0.51±0.16
**bAib**	EN	0.061±0.024	0.059±0.023	0.058±0.024	0.059±0.022	0.062±0.026	0.063±0.021	0.065±0.027	0.062±0.025	0.060±0.025	0.060±0.022	0.064±0.022	0.059±0.021
	SP	0.060±0.018	0.058±0.017	0.059±0.021	0.065±0.022	0.061±0.019	--	0.065±0.022	0.065±0.022	0.066±0.023	0.061±0.023	0.061±0.020	0.062±0.021
**bAla**	EN	1.18±0.32	1.24±0.24	1.19±0.21	1.24±0.23	1.17±0.21	1.20±0.15	1.11±0.19	1.20±0.28	1.23±0.30	1.24±0.31	1.23±0.31	1.32±0.28
	SP	1.01±0.21	0.96±0.50	0.84±0.27	0.99±0.29	0.70±0.30	--	0.80±0.30	0.94±0.28	1.00±0.30	1.05±0.23	1.03±0.37	0.81±0.26
**Cit**	EN	1.04±0.57	0.95±0.48	0.93±0.40	0.90±0.38	0.92±0.40	0.86±0.32	0.84±0.29	0.80±0.26	0.78±0.27	0.81±0.21	0.78±0.25	0.79±0.23
	SP	0.73±0.13	0.69±0.10	0.69±0.10	0.68±0.11	0.64±0.10	--	0.65±0.11	0.69±0.09	0.67±0.11	0.69±0.11	0.69±0.10	0.69±0.11
**EtN**	EN	0.29±0.05	0.31±0.05	0.31±0.06	0.32±0.05	0.34±0.06	0.37±0.06	0.39±0.06	0.35±0.06	0.35±0.06	0.35±0.07	0.33±0.06	0.32±0.05
	SP	0.24±0.04	0.25±0.04	0.27±0.04	0.28±0.06	0.29±0.05	--	0.30±0.05	0.29±0.05	0.28±0.05	0.27±0.05	0.26±0.06	0.24±0.05
**Hyp**	EN	0.294±0.157	0.265±0.138	0.260±0.134	0.244±0.124	0.246±0.126	0.226±0.123	0.207±0.118	0.2150.132±	0.228±0.145	0.249±0.156	0.249±0.148	0.260±0.127
	SP	0.313±0.108	0.268±0.094	0.267±0.091	0.255±0.084	0.226±0.073	--	0.226±0.077	0.244±0.093	0.249±0.086	0.270±0.096	0.276±0.105	0.280±0.105
**Orn**	EN	2.15±0.82	1.90±0.60	1.84±0.66	1.85±0.62	1.88±0.65	1.80±0.64	1.79±0.61	1.64±0.58	1.64±0.60	1.65±0.60	1.64±0.66	1.68±0.54
	SP	1.38±0.24	1.26±0.25	1.19±0.22	1.22±0.22	1.18±0.20	--	1.23±0.24	1.22±0.36	1.19±0.35	1.19±0.25	1.16±0.24	1.22±0.28
**PEtN**	EN	0.065±0.033	0.046±0.018	0.050±0.021	0.054±0.020	0.063±0.027	0.053±0.013	0.064±0.021	0.066±0.028	0.063±0.035	0.063±0.039	0.053±0.026	0.047±0.026
	SP	0.036±0.010	0.030±0.007	0.033±0.006	0.037±0.006	0.040±0.006	--	0.043±0.007	0.047±0.006	0.044±0.008	0.042±0.008	0.037±0.008	0.036±0.007
**Sar**	EN	0.137±0.026	0.131±0.026	0.118±0.028	0.111±0.027	0.115±0.028	0.115±0.025	0.108±0.029	0.108±0.029	0.110±0.030	0.117±0.027	0.113±0.029	0.123±0.032
	SP	0.117±0.013	0.109±0.019	0.107±0.013	0.112±0.017	0.095±0.012	--	0.096±0.011	0.106±0.011	0.106±0.008	0.113±0.013	0.111±0.018	0.104±0.011
**Tau**	EN	1.33±0.39	1.15±0.17	1.21±0.20	1.25±0.29	1.37±0.37	1.24±0.22	1.32±0.32	1.34±0.36	1.37±0.45	1.41±0.54	1.30±0.38	1.25±0.33
	SP	0.84±0.15	0.76±0.12	0.77±0.13	0.80±0.15	0.81±0.14	--	0.85±0.16	0.89±0.16	0.88±0.16	0.89±0.18	0.84±0.14	0.82±0.14
**Prot.**	EN	89.4±11.4	79.4±9.4	77.6±8.5	76.1±8.9	78.7±9.8	76.9±9.2	77.510.8±	75.3±10.7	77.0±12.1	80.6±14.1	80.7±15.0	84.8±13.7
	SP	72.8±13.0	65.3±7.3	66.9±7.5	67.9±7.6	65.7±6.9	--	67.3±8.2	67.1±7.6	67.8±8.0	69.8±8.9	70.6±7.7	71.6±7.9
**Ess.**	EN	33.8±5.9	28.9±3.6	28.0±3.2	27.3±3.3	27.8±3.4	26.4±2.9	25.9±3.5	25.7±3.8	26.1±4.2	27.2±5.1	27.2±5.4	28.0±4.4
	SP	28.7±7.9	25.3±5.4	25.2±5.2	25.3±5.1	23.9±4.3	--	24.2±4.4	24.6±4.5	24.5±4.0	25.3±4.2	25.3±3.7	25.6±4.3
**n-Ess.**	EN	55.6±6.7	50.6±6.4	49.6±5.7	48.8±5.9	50.9±6.7	50.5±6.6	51.6±7.5	49.6±7.1	50.9±8.1	53.4±9.3	53.5±9.9	56.8±9.6
	SP	44.0±6.1	40.0±2.9	41.7±3.7	42.6±3.7	41.8±3.5	--	43.1±4.7	42.5±4.6	43.3±5.7	44.5±6.3	45.2±5.9	46.0±5.5
**BCAA**	EN	15.7±3.9	13.1±2.1	12.6±1.8	12.3±1.8	12.5±1.8	11.9±1.3	11.6±1.5	11.9±1.5	12.0±1.8	12.4±2.3	12.3±2.2	12.5±1.9
	SP	13.5±4.7	11.9±3.5	11.9±3.3	11.9±3.4	11.2±2.9	--	11.4±2.8	12.0±3.0	11.7±2.7	12.1±2.6	12.2±2.7	12.1±3.1
**n-Prot.**	EN	7.86±1.60	7.21±1.15	7.10±1.26	7.14±1.21	7.28±1.32	6.98±1.01	6.90±1.11	6.79±1.22	6.90±1.38	7.06±1.51	6.89±1.22	7.02±1.05
	SP	5.72±0.78	5.29±1.02	5.12±0.61	5.35±1.01	4.84±0.55	--	5.08±0.73	5.35±0.73	5.38±0.88	5.52±0.88	5.40±0.93	5.21±0.61
**Total**	EN	97.3±12.4	86.6±10.1	84.7±9.4	83.2±9.7	86.0±10.8	83.9±9.9	84.4±11.7	82.1±11.8	83.9±13.4	87.7±15.5	87.6±16.2	91.9±14.6
	SP	78.5±13.4	70.6±7.7	72.1±7.8	73.2±8.3	70.5±7.1	--	72.4±8.6	72.4±8.2	73.2±8.7	75.4±9.6	76.0±8.4	76.8±8.3
