# Supplementary material for: Exercise-induced response of proteinogenic and non-proteinogenic plasma free amino acids is sport-specific: A comparison of sprint and endurance athletes
Source: PLoS One. 2024 Aug 30;19(8):e0309529. doi: 10.1371/journal.pone.0309529 (PMC11364291; doi:10.1371/journal.pone.0309529)
Supplement: S1 Fig — (PDF) [file pone.0309529.s003.PDF]

S1 Figures The time course of the individual PFAAs concentrations (absolute values) in endurance (open circles, dashed line) and sprint-trained (black squares, solid line) athletes before exercise, during the progressive test until exhaustion (Ex), and post-exercise recovery. For the full names of the amino acids, see the list of abbreviations in the main text.

Explanation: ANOVA main effects for the group (sports specialty), stage (exercise and recovery phase), and their interaction are shown in bold, if statistically significant. Bonferroni post hoc tests are denoted as follows: * different from the sprint-trained group at the same test stage (phase), # different from pre-exercise values, † different from values at exhaustion. If there was no group effect or interaction, post hoc test markings apply to both sports groups.

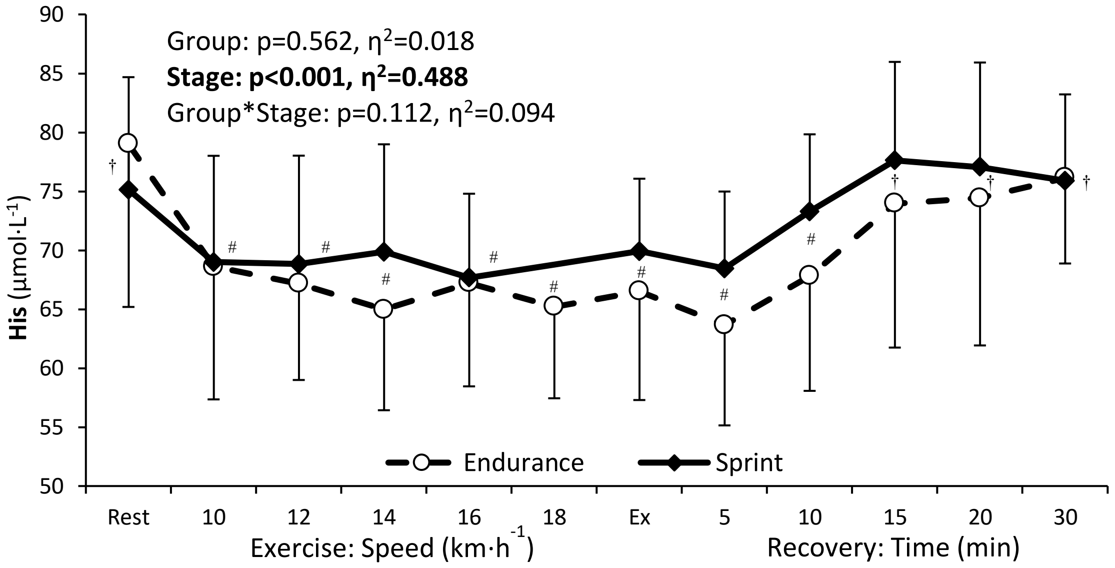

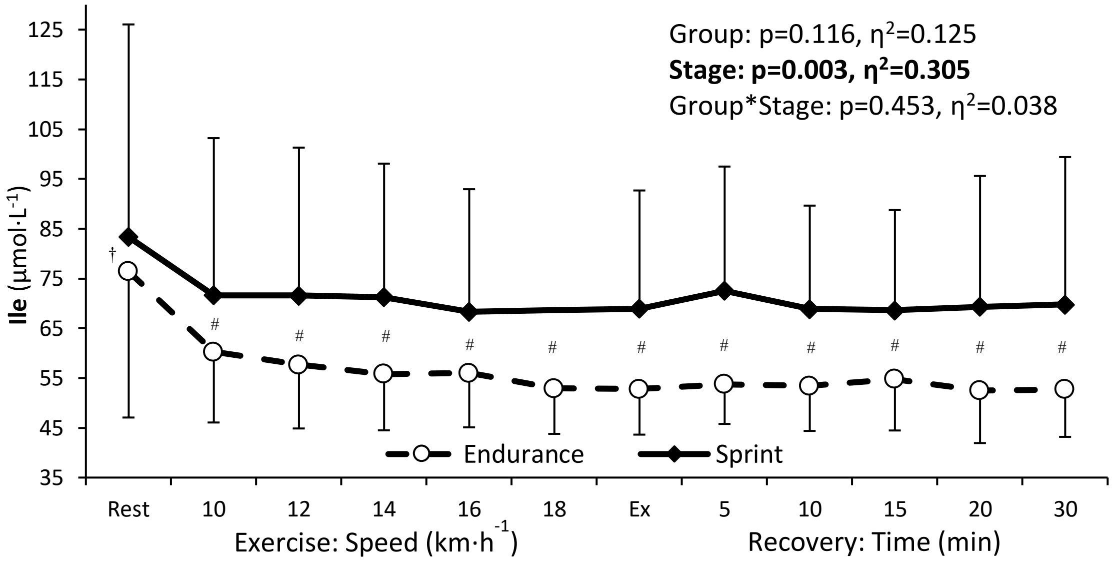

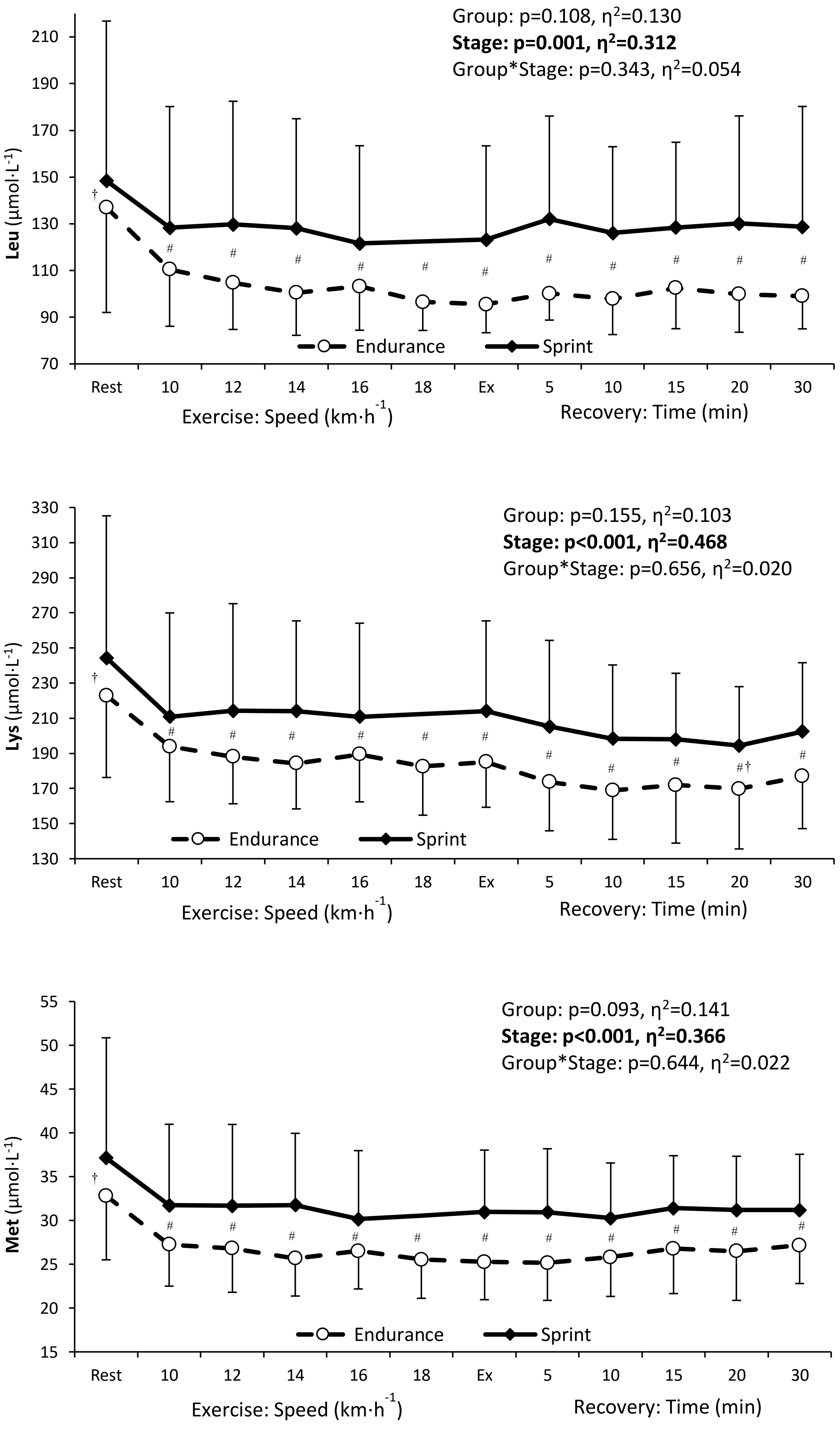

Group: p=0.108, η2=0.130
Stage: p=0.001, η2=0.312
Group*Stage: p=0.343, η2=0.054
Leu (µmol·L-1)
Endurance
Sprint
Rest 10 12 14 16 18 Ex 5 10 15 20 30
Exercise: Speed (km·h-1)
Recovery: Time (min)
Group: p=0.155, η2=0.103
Stage: p<0.001, η2=0.468
Group*Stage: p=0.656, η2=0.020
Lys (µmol·L-1)
Endurance
Sprint
Rest 10 12 14 16 18 Ex 5 10 15 20 30
Exercise: Speed (km·h-1)
Recovery: Time (min)
Group: p=0.093, η2=0.141
Stage: p<0.001, η2=0.366
Group*Stage: p=0.644, η2=0.022
Met (µmol·L-1)
Endurance
Sprint
Rest 10 12 14 16 18 Ex 5 10 15 20 30
Exercise: Speed (km·h-1)
Recovery: Time (min)

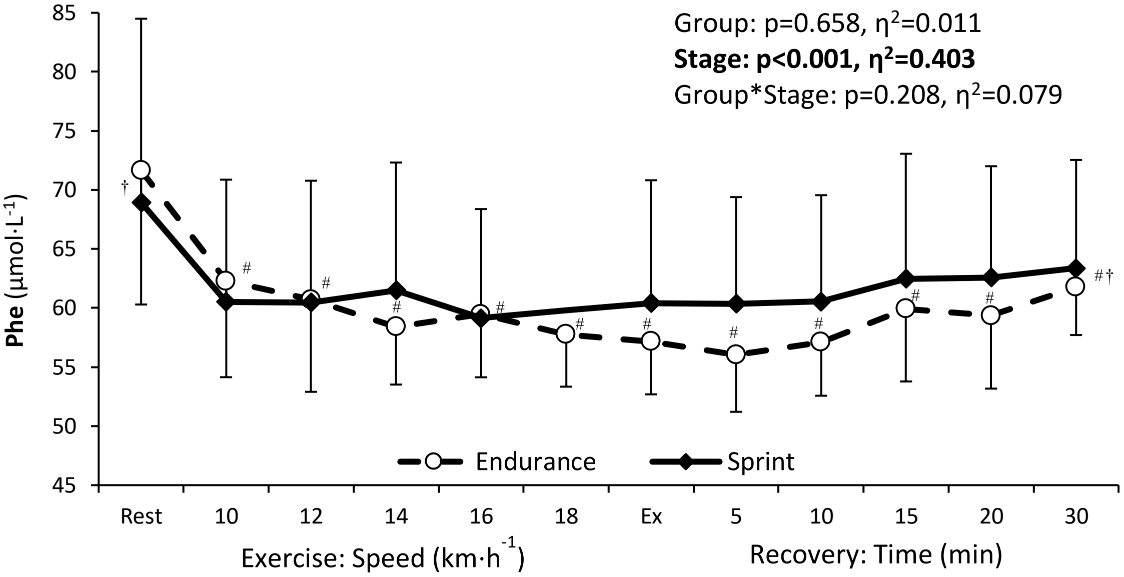
Group: p=0.658, η2=0.011
Stage: p<0.001, η2=0.403
Group*Stage: p=0.208, η2=0.079
Phe (μmol·L-1)
85 80 75 70 65 60 55 50 45
Endurance
Sprint
Rest 10 12 14 16 18 Ex 5 10 15 20 30
Exercise: Speed (km·h-1)
Recovery: Time (min)

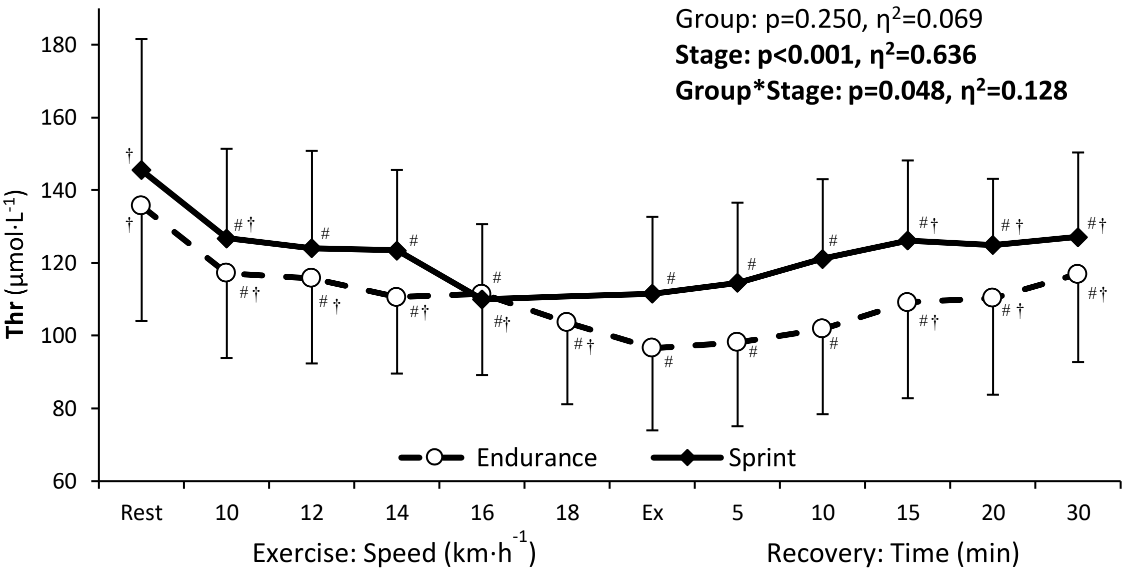
Group: p=0.250, η2=0.069
Stage: p<0.001, η2=0.636
Group*Stage: p=0.048, η2=0.128
Thr (μmol·L-1)
180 160 140 120 100 80 60
Endurance
Sprint
Rest 10 12 14 16 18 Ex 5 10 15 20 30
Exercise: Speed (km·h-1)
Recovery: Time (min)

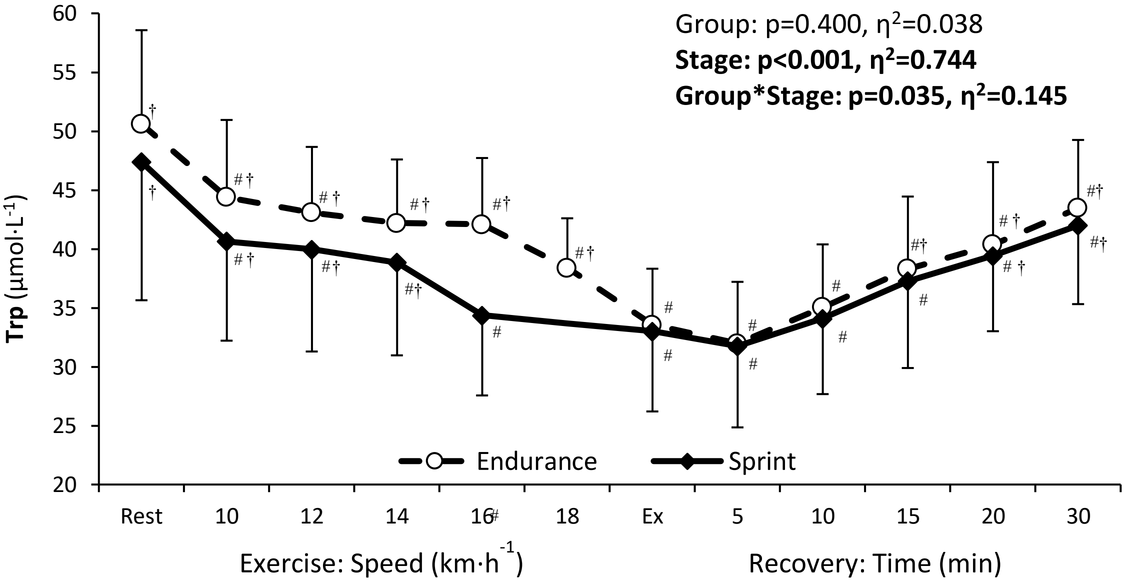
Group: p=0.400, η2=0.038
Stage: p<0.001, η2=0.744
Group*Stage: p=0.035, η2=0.145
Trp (μmol·L-1)
60 55 50 45 40 35 30 25 20
Endurance
Sprint
Rest 10 12 14 16 18 Ex 5 10 15 20 30
Exercise: Speed (km·h-1)
Recovery: Time (min)

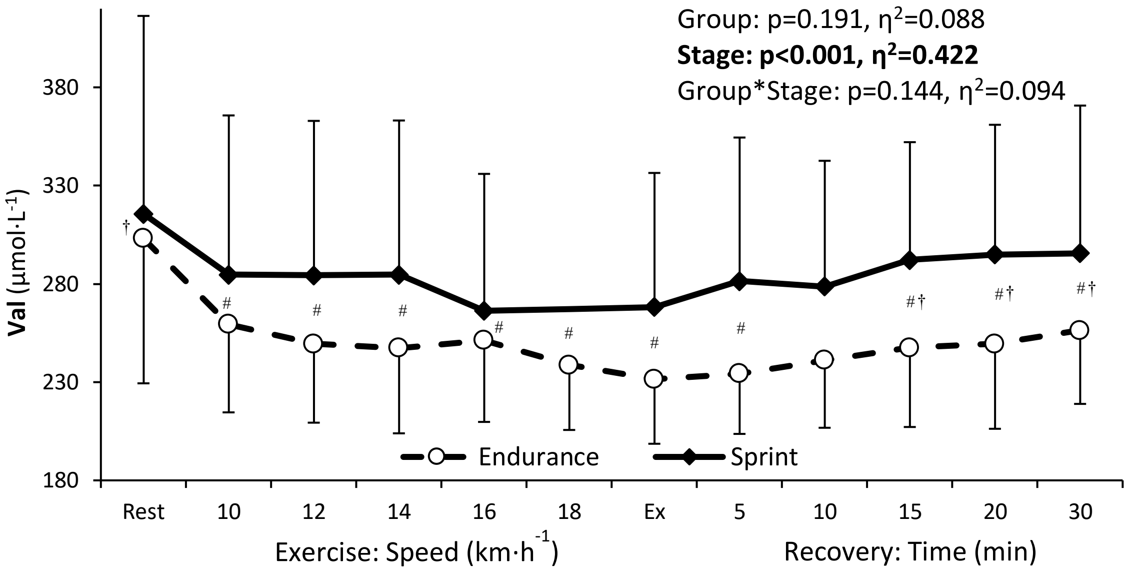
Group: p=0.191, η2=0.088
Stage: p<0.001, η2=0.422
Group*Stage: p=0.144, η2=0.094
Val (μmol·L-1)
Endurance
Sprint
Rest 10 12 14 16 18 Ex 5 10 15 20 30
Exercise: Speed (km·h-1)
Recovery: Time (min)

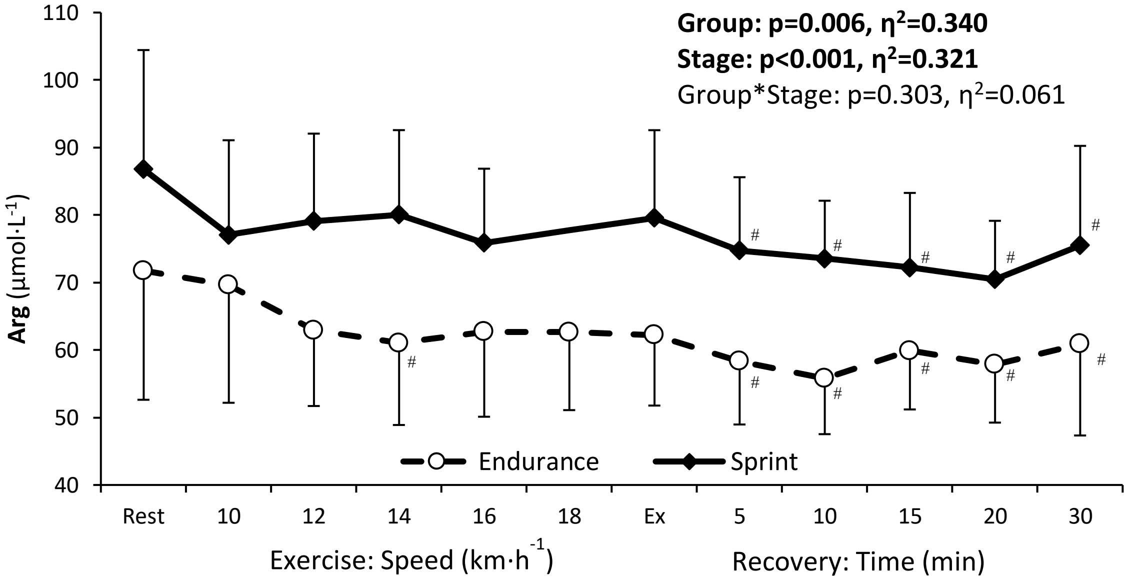
Group: p=0.006, η2=0.340
Stage: p<0.001, η2=0.321
Group*Stage: p=0.303, η2=0.061
Arg (μmol·L-1)
Endurance
Sprint
Rest 10 12 14 16 18 Ex 5 10 15 20 30
Exercise: Speed (km·h-1)
Recovery: Time (min)

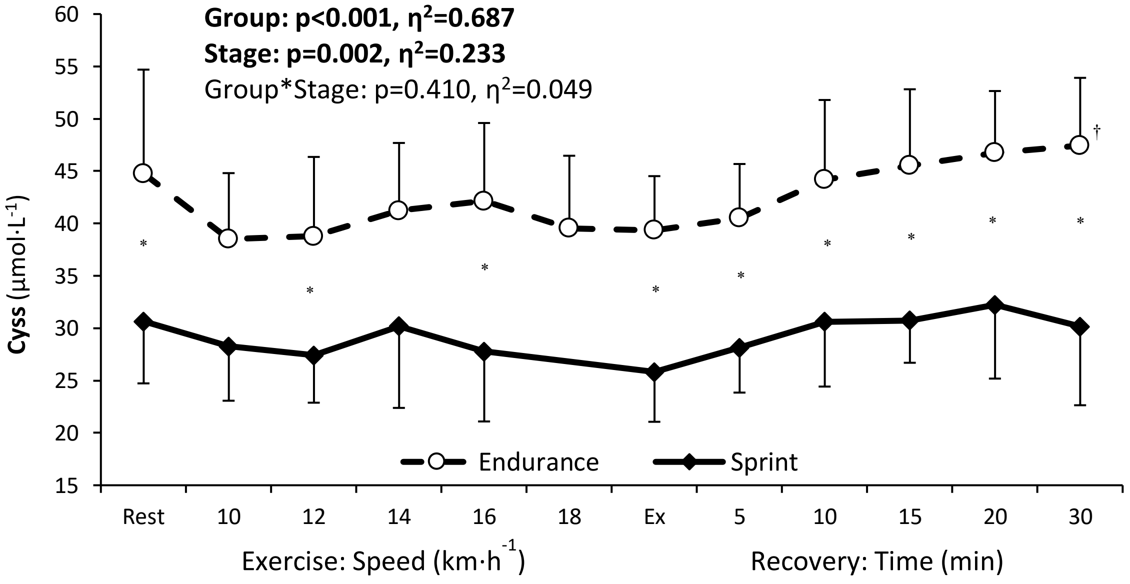
Group: p<0.001, η2=0.687
Stage: p=0.002, η2=0.233
Group*Stage: p=0.410, η2=0.049
Cyss (μmol·L-1)
Endurance
Sprint
Rest 10 12 14 16 18 Ex 5 10 15 20 30
Exercise: Speed (km·h-1)
Recovery: Time (min)

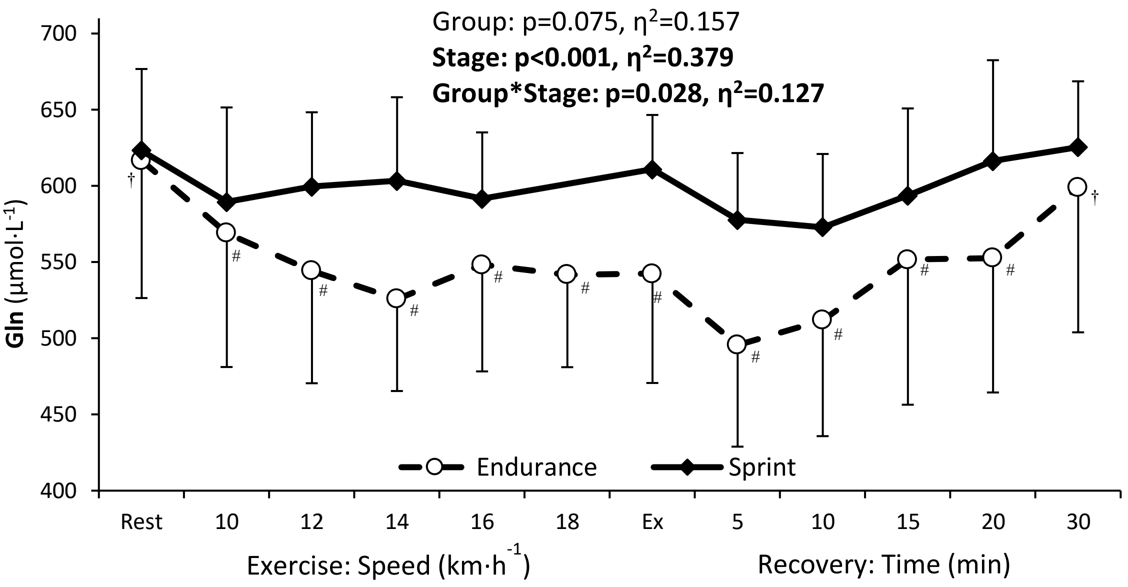
Group: p=0.075, η2=0.157
Stage: p<0.001, η2=0.379
Group*Stage: p=0.028, η2=0.127
Gln (µmol·L-1)
Endurance
Sprint
Rest
10
12
14
16
18
Ex
5
10
15
20
30
Exercise: Speed (km·h-1)
Recovery: Time (min)

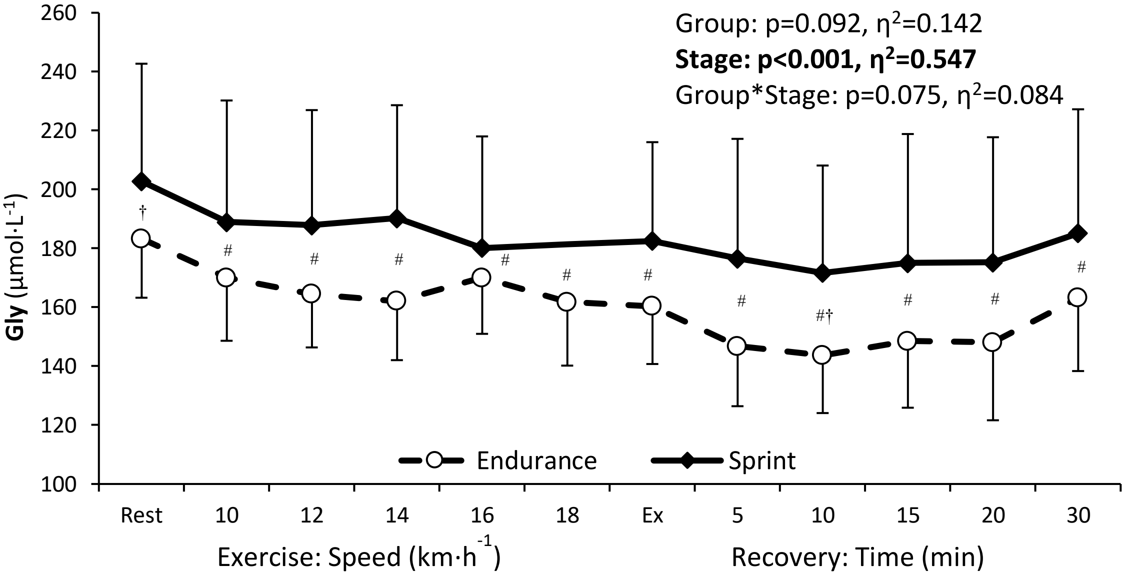
Group: p=0.092, η2=0.142
Stage: p<0.001, η2=0.547
Group*Stage: p=0.075, η2=0.084
Gly (µmol·L-1)
Endurance
Sprint
Exercise: Speed (km·h-1)
Recovery: Time (min)

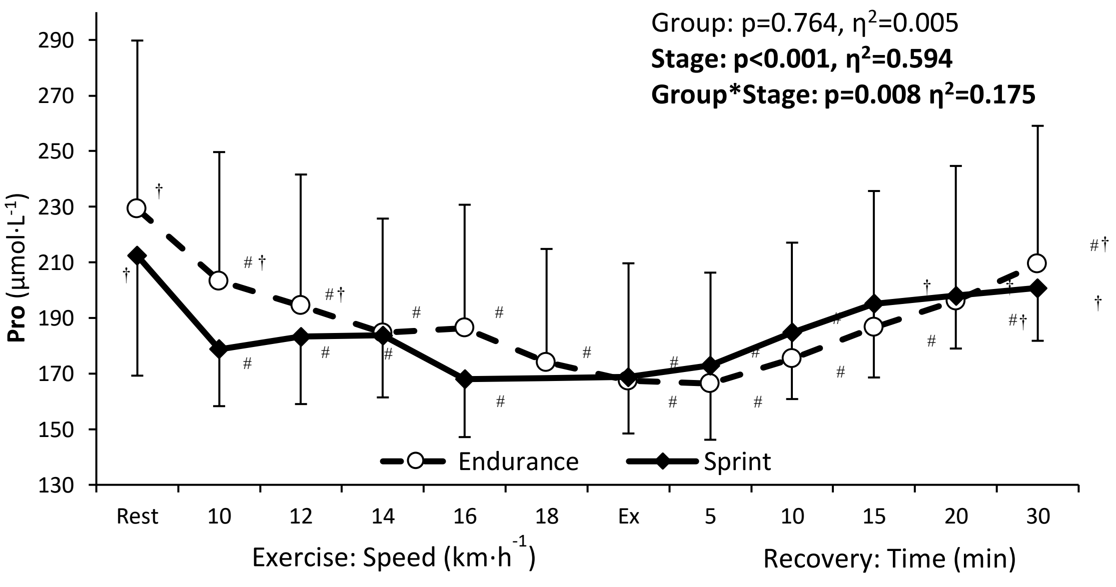
Group: p=0.764, η2=0.005
Stage: p<0.001, η2=0.594
Group*Stage: p=0.008 η2=0.175
Pro (µmol·L-1)
Endurance
Sprint
Exercise: Speed (km·h-1)
Recovery: Time (min)

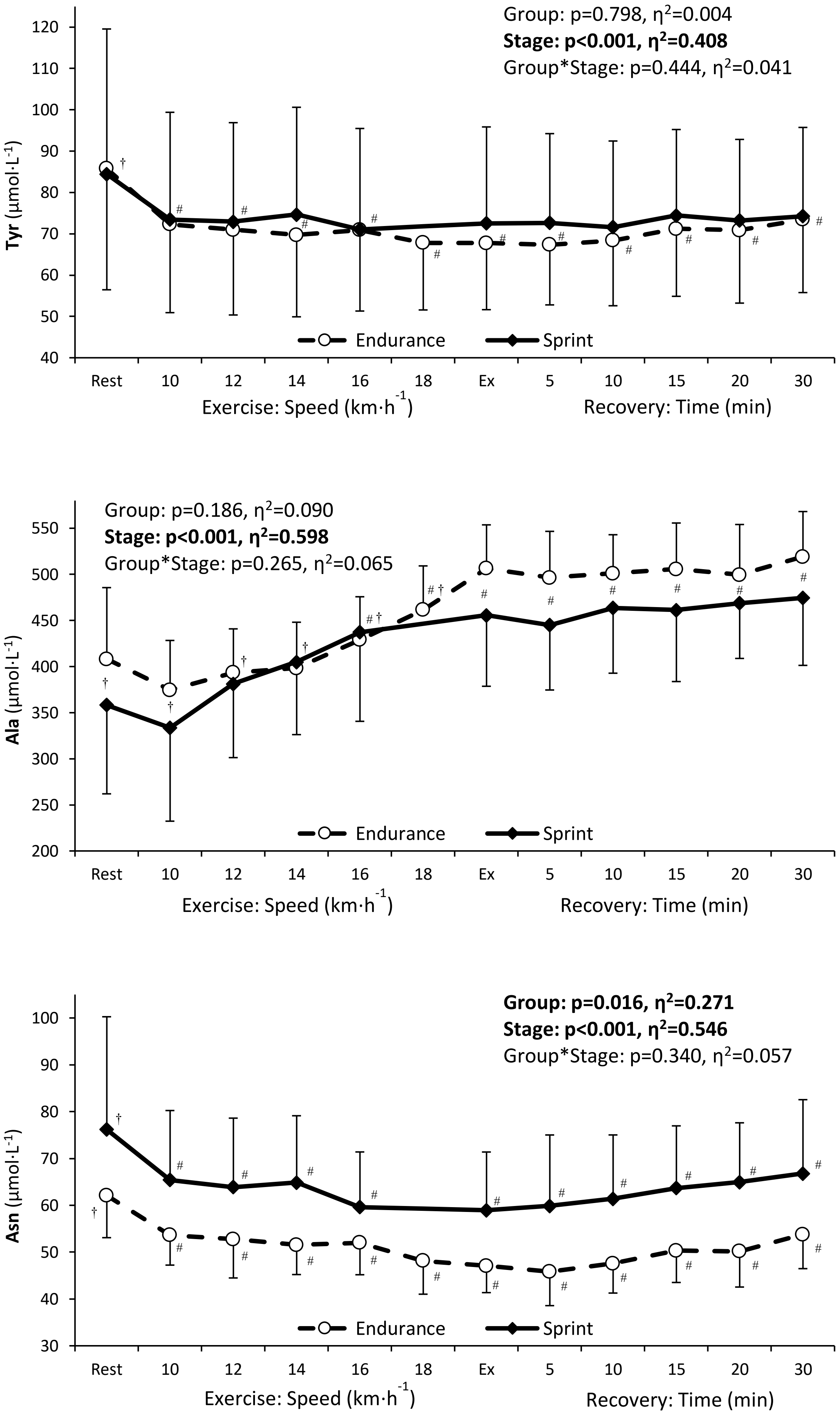

Group: p=0.798, η2=0.004
Stage: p<0.001, η2=0.408
Group*Stage: p=0.444, η2=0.041
Tyr (µmol·L-1)
Endurance
Sprint
Rest 10 12 14 16 18 Ex 5 10 15 20 30
Exercise: Speed (km·h-1)
Recovery: Time (min)
Group: p=0.186, η2=0.090
Stage: p<0.001, η2=0.598
Group*Stage: p=0.265, η2=0.065
Ala (µmol·L-1)
Group: p=0.016, η2=0.271
Stage: p<0.001, η2=0.546
Group*Stage: p=0.340, η2=0.057
Asn (µmol·L-1)

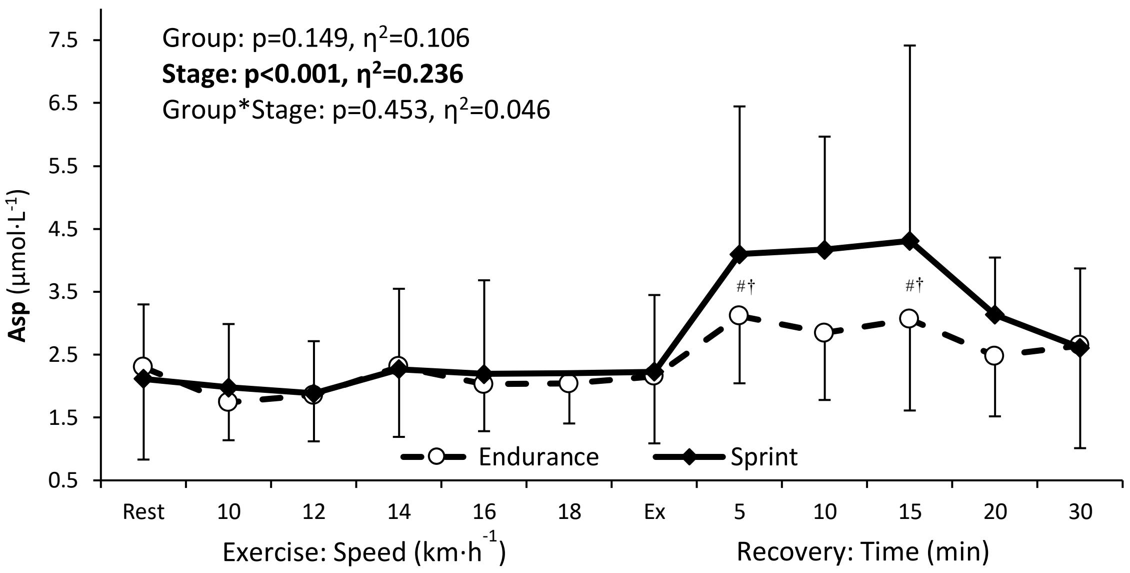
Group: p=0.149, η^2=0.106
Stage: p<0.001, η^2=0.236
Group*Stage: p=0.453, η^2=0.046
Asp (µmol·L^{-1})
Endurance
Sprint
Rest 10 12 14 16 18 Ex 5 10 15 20 30
Exercise: Speed (km·h^{-1})
Recovery: Time (min)

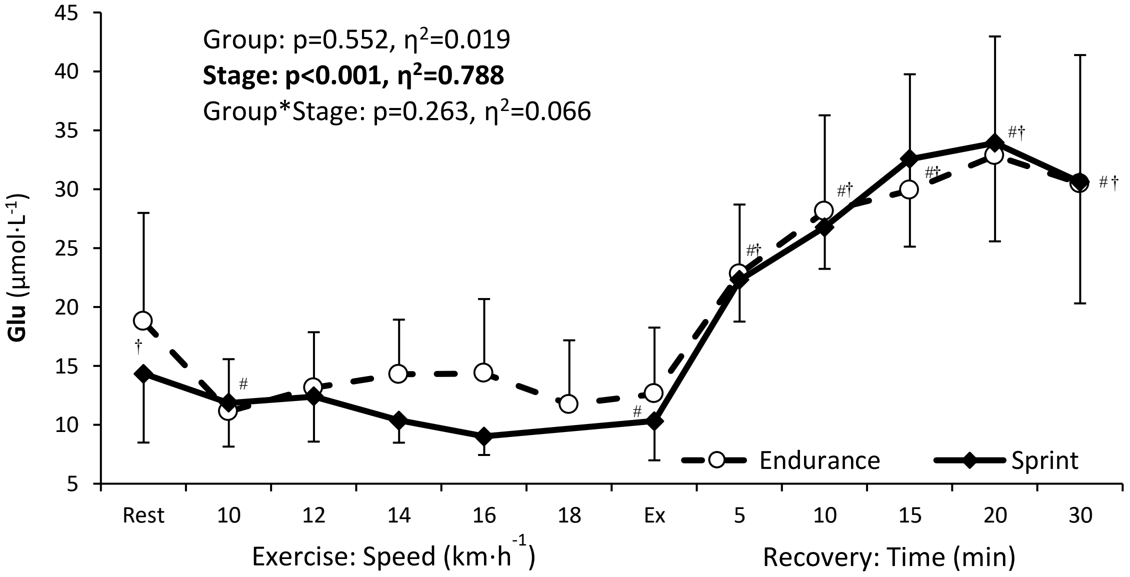
Group: p=0.552, η^2=0.019
Stage: p<0.001, η^2=0.788
Group*Stage: p=0.263, η^2=0.066
Glu (µmol·L^{-1})
Endurance
Sprint
Rest 10 12 14 16 18 Ex 5 10 15 20 30
Exercise: Speed (km·h^{-1})
Recovery: Time (min)

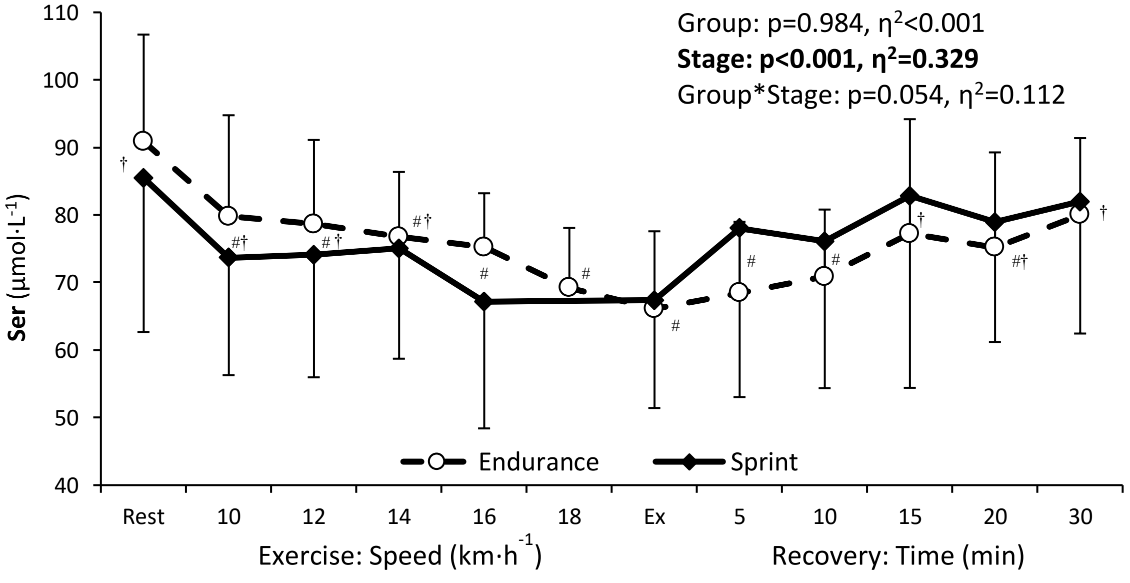
Group: p=0.984, η^2<0.001
Stage: p<0.001, η^2=0.329
Group*Stage: p=0.054, η^2=0.112
Ser (µmol·L^{-1})
Endurance
Sprint
Rest 10 12 14 16 18 Ex 5 10 15 20 30
Exercise: Speed (km·h^{-1})
Recovery: Time (min)

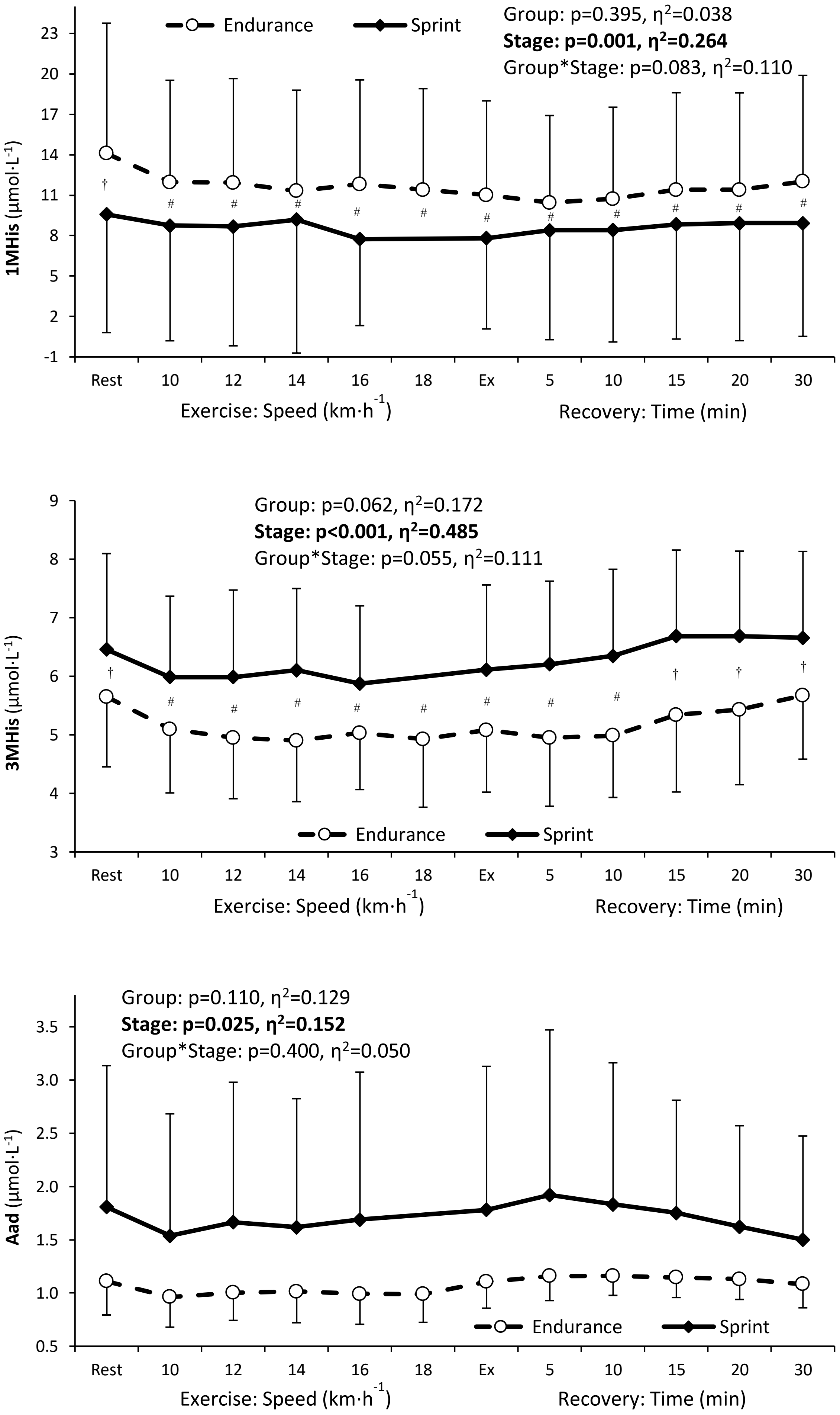

Endurance
Sprint
Group: p=0.395, η2=0.038
Stage: p=0.001, η2=0.264
Group*Stage: p=0.083, η2=0.110
1MHis (µmol·L-1)
Rest 10 12 14 16 18 Ex 5 10 15 20 30
Exercise: Speed (km·h-1)
Recovery: Time (min)
Group: p=0.062, η2=0.172
Stage: p<0.001, η2=0.485
Group*Stage: p=0.055, η2=0.111
3MHis (µmol·L-1)
Exercise: Speed (km·h-1)
Recovery: Time (min)
Group: p=0.110, η2=0.129
Stage: p=0.025, η2=0.152
Group*Stage: p=0.400, η2=0.050
Aad (µmol·L-1)
Exercise: Speed (km·h-1)
Recovery: Time (min)

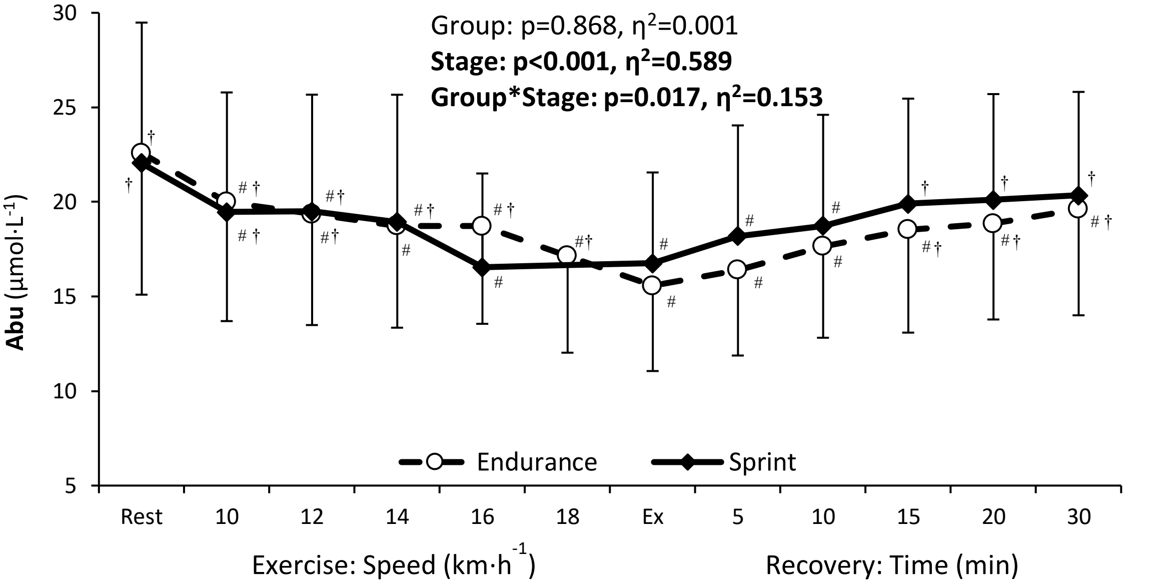
Group: p=0.868, η2=0.001
Stage: p<0.001, η2=0.589
Group*Stage: p=0.017, η2=0.153
Abu (µmol·L-1)
Endurance
Sprint
Rest 10 12 14 16 18 Ex 5 10 15 20 30
Exercise: Speed (km·h-1)
Recovery: Time (min)

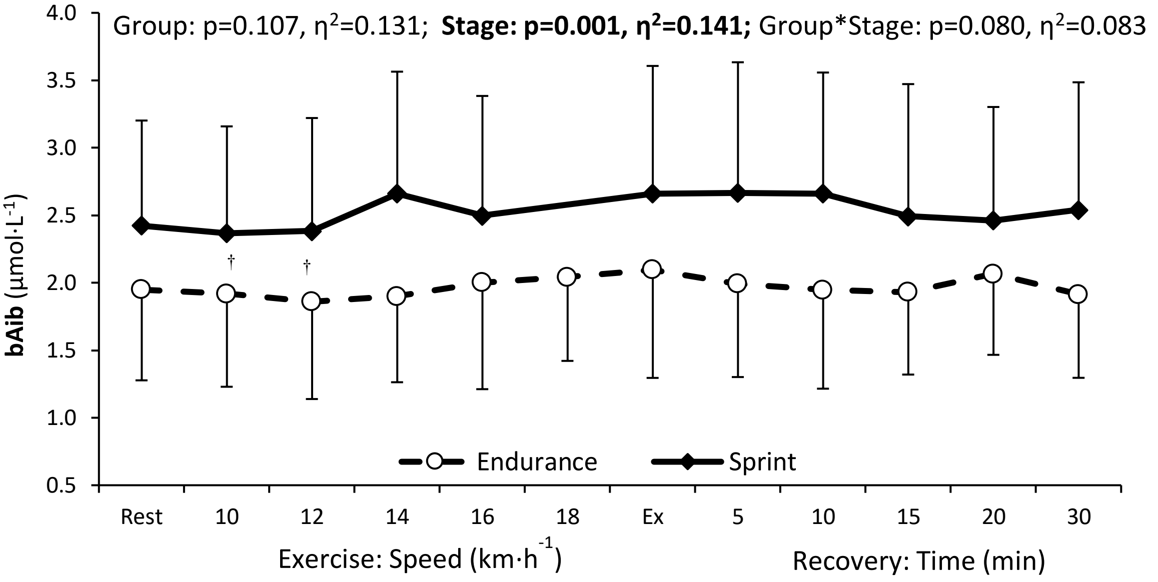
Group: p=0.107, η2=0.131; Stage: p=0.001, η2=0.141; Group*Stage: p=0.080, η2=0.083
bAib (µmol·L-1)
Endurance
Sprint
Rest 10 12 14 16 18 Ex 5 10 15 20 30
Exercise: Speed (km·h-1)
Recovery: Time (min)

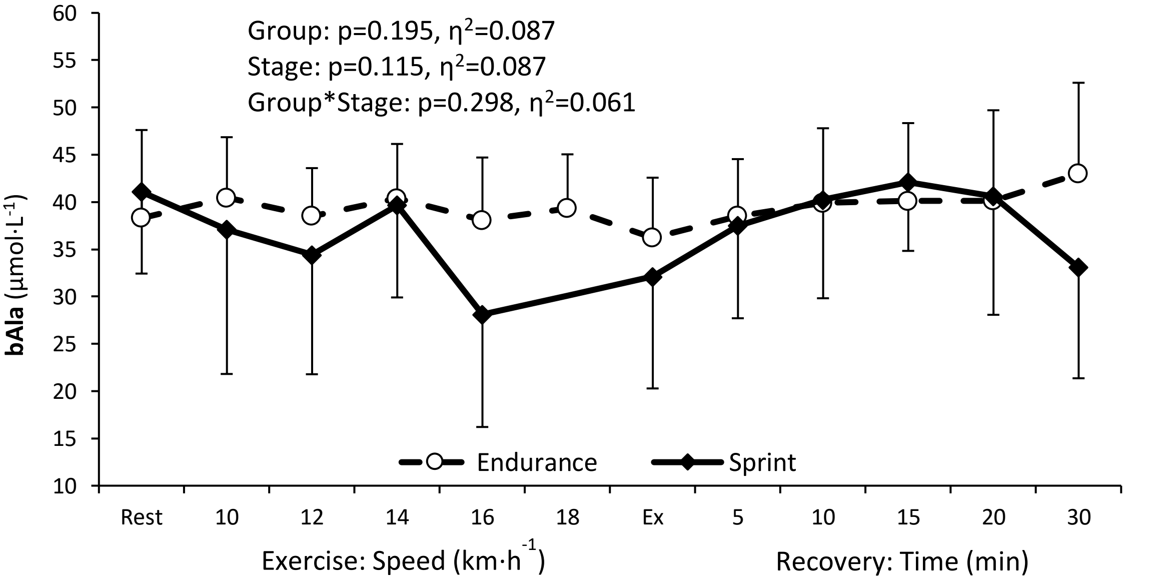
Group: p=0.195, η2=0.087
Stage: p=0.115, η2=0.087
Group*Stage: p=0.298, η2=0.061
bAla (µmol·L-1)
Endurance
Sprint
Rest 10 12 14 16 18 Ex 5 10 15 20 30
Exercise: Speed (km·h-1)
Recovery: Time (min)

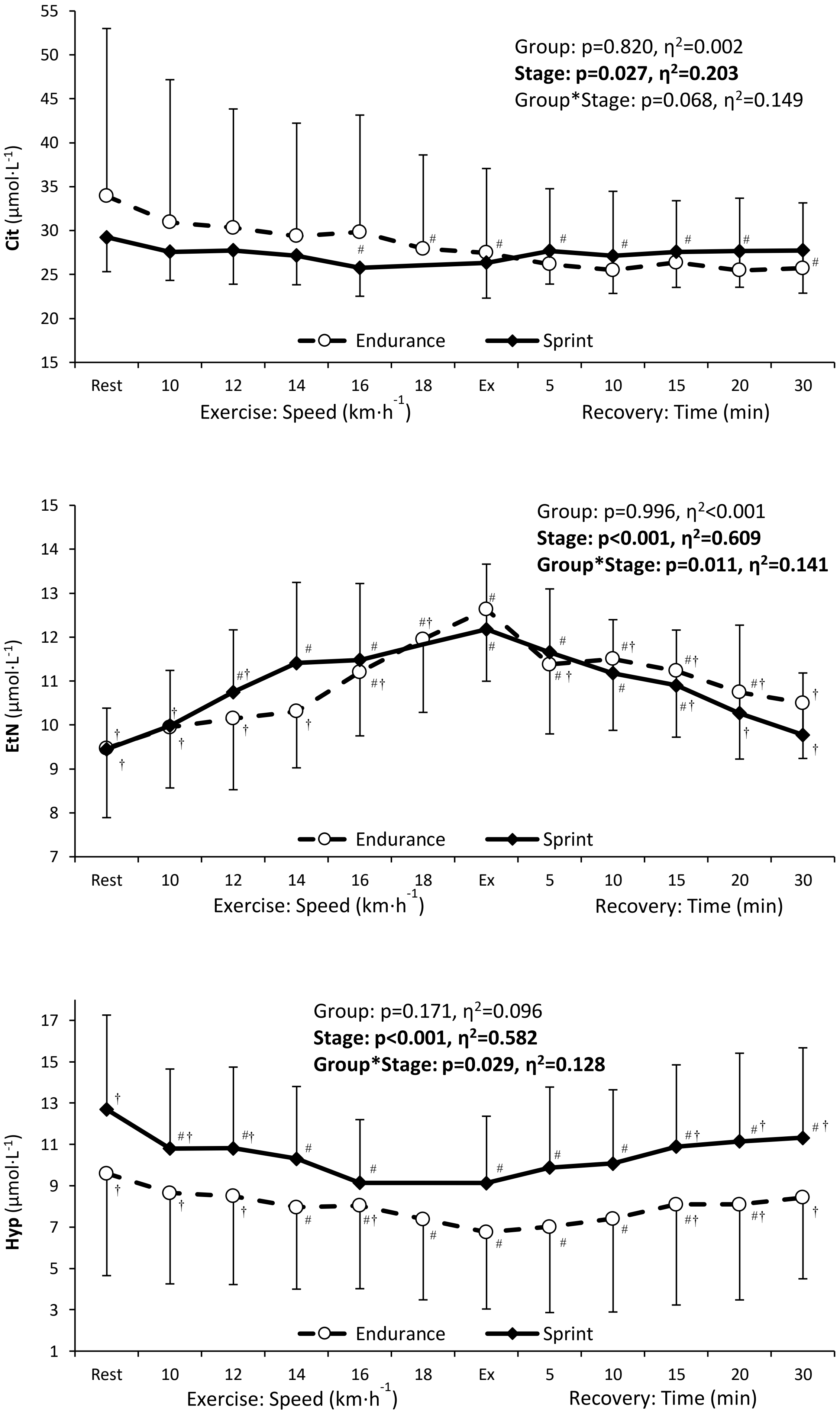
Group: $p=0.820$, $\eta^2=0.002$
Stage: $p=0.027$, $\eta^2=0.203$
Group*Stage: $p=0.068$, $\eta^2=0.149$
Cit (µmol·L^{-1})
Endurance
Sprint
Rest 10 12 14 16 18 Ex 5 10 15 20 30
Exercise: Speed (km·h^{-1})
Recovery: Time (min)
Group: $p=0.996$, $\eta^2<0.001$
Stage: $p<0.001$, $\eta^2=0.609$
Group*Stage: $p=0.011$, $\eta^2=0.141$
EtN (µmol·L^{-1})
Endurance
Sprint
Rest 10 12 14 16 18 Ex 5 10 15 20 30
Exercise: Speed (km·h^{-1})
Recovery: Time (min)
Group: $p=0.171$, $\eta^2=0.096$
Stage: $p<0.001$, $\eta^2=0.582$
Group*Stage: $p=0.029$, $\eta^2=0.128$
Hyp (µmol·L^{-1})
Endurance
Sprint
Rest 10 12 14 16 18 Ex 5 10 15 20 30
Exercise: Speed (km·h^{-1})
Recovery: Time (min)

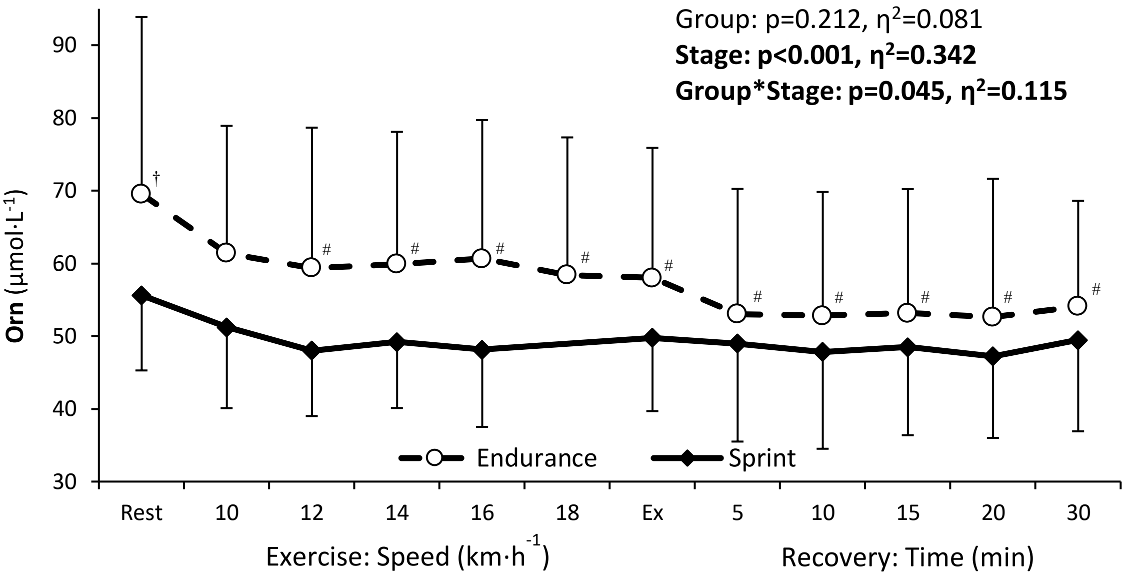
Group: p=0.212, η2=0.081
Stage: p<0.001, η2=0.342
Group*Stage: p=0.045, η2=0.115
Orn (µmol·L-1)
Endurance
Sprint
Rest 10 12 14 16 18 Ex 5 10 15 20 30
Exercise: Speed (km·h-1)
Recovery: Time (min)

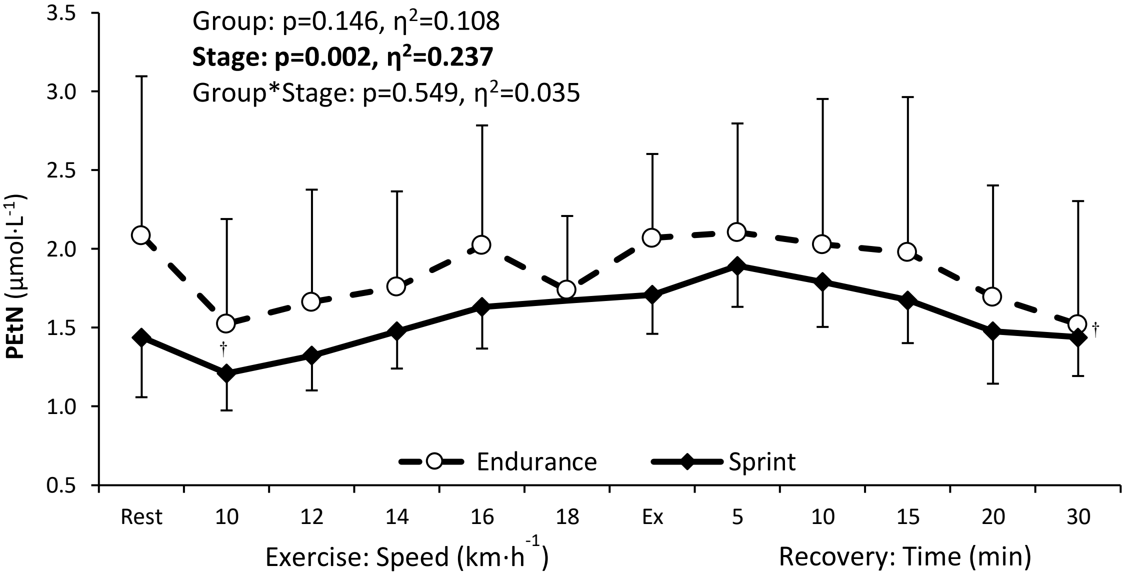
Group: p=0.146, η2=0.108
Stage: p=0.002, η2=0.237
Group*Stage: p=0.549, η2=0.035
PEtN (µmol·L-1)
Endurance
Sprint
Rest 10 12 14 16 18 Ex 5 10 15 20 30
Exercise: Speed (km·h-1)
Recovery: Time (min)

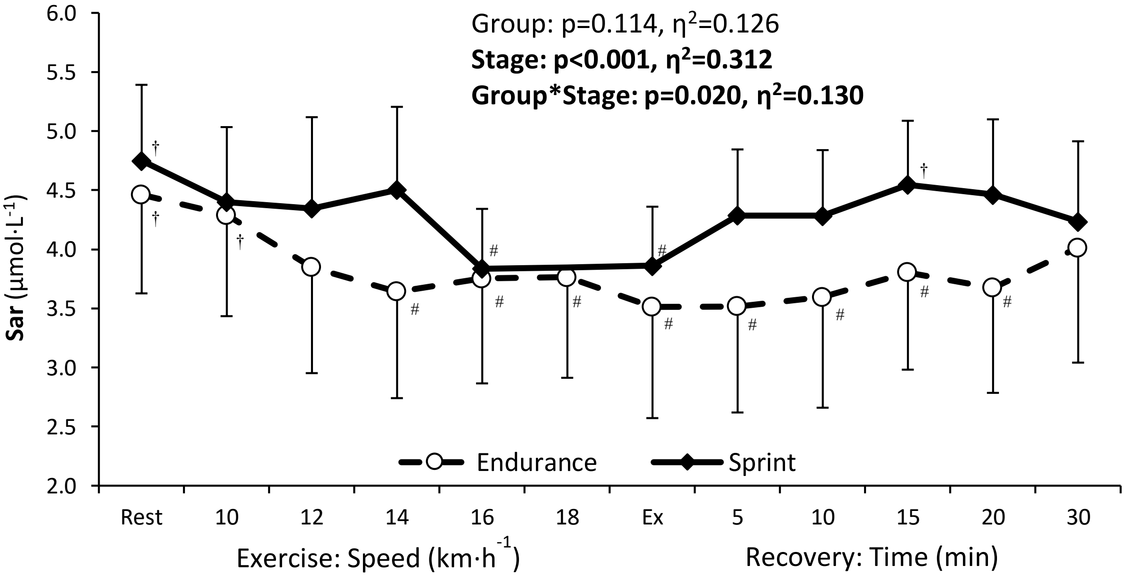
Group: p=0.114, η2=0.126
Stage: p<0.001, η2=0.312
Group*Stage: p=0.020, η2=0.130
Sar (µmol·L-1)
Endurance
Sprint
Rest 10 12 14 16 18 Ex 5 10 15 20 30
Exercise: Speed (km·h-1)
Recovery: Time (min)

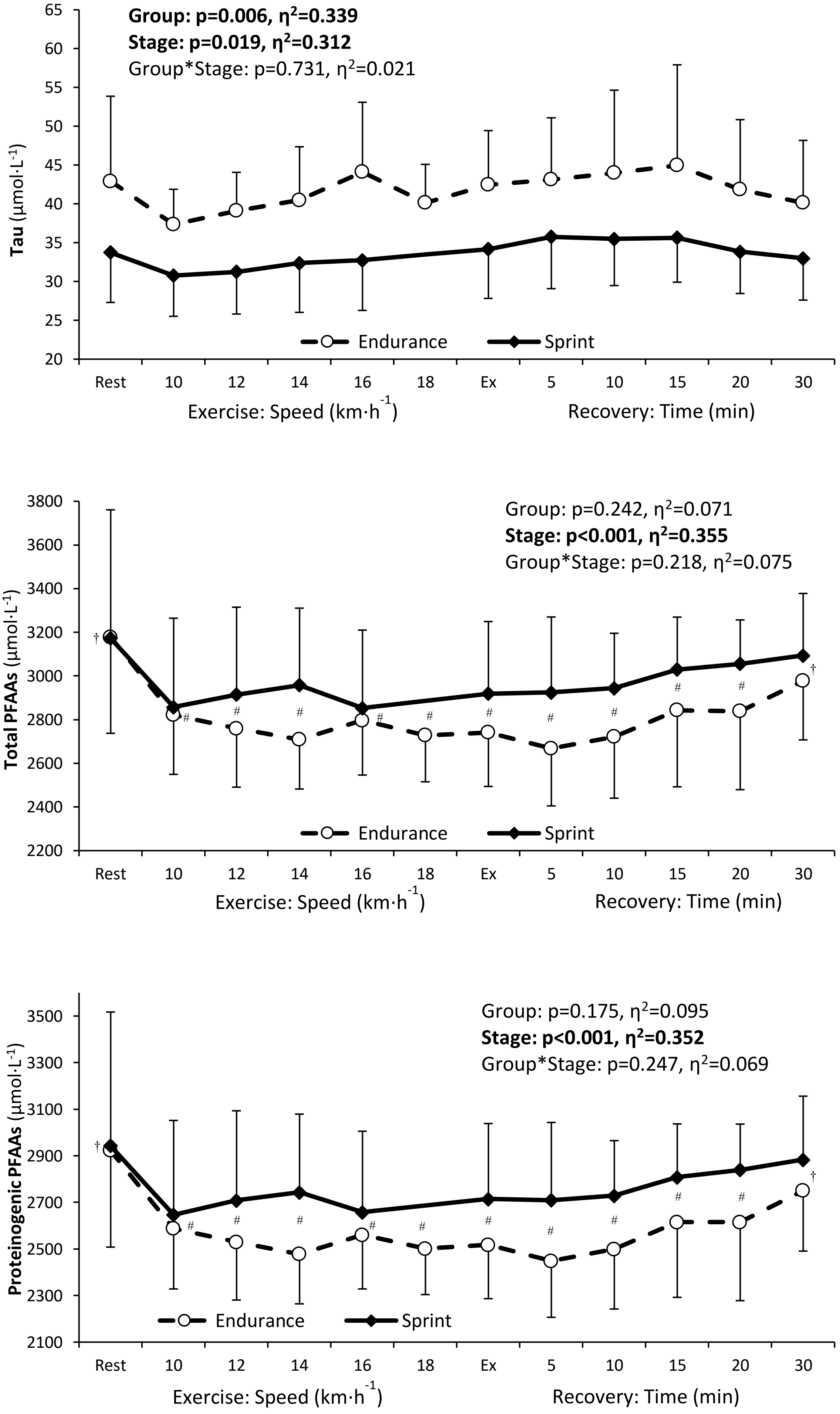

Group: p=0.006, η2=0.339
Stage: p=0.019, η2=0.312
Group*Stage: p=0.731, η2=0.021
Tau (μmol·L-1)
65
60
55
50
45
40
35
30
25
20
Endurance
Sprint
Rest
10
12
14
16
18
Ex
5
10
15
20
30
Exercise: Speed (km·h-1)
Recovery: Time (min)
Group: p=0.242, η2=0.071
Stage: p<0.001, η2=0.355
Group*Stage: p=0.218, η2=0.075
Total PFAAs (μmol·L-1)
3800
3600
3400
3200
3000
2800
2600
2400
2200
Endurance
Sprint
Rest
10
12
14
16
18
Ex
5
10
15
20
30
Exercise: Speed (km·h-1)
Recovery: Time (min)
Group: p=0.175, η2=0.095
Stage: p<0.001, η2=0.352
Group*Stage: p=0.247, η2=0.069
Proteinogenic PFAAs (μmol·L-1)
3500
3300
3100
2900
2700
2500
2300
2100
Endurance
Sprint
Rest
10
12
14
16
18
Ex
5
10
15
20
30
Exercise: Speed (km·h-1)
Recovery: Time (min)

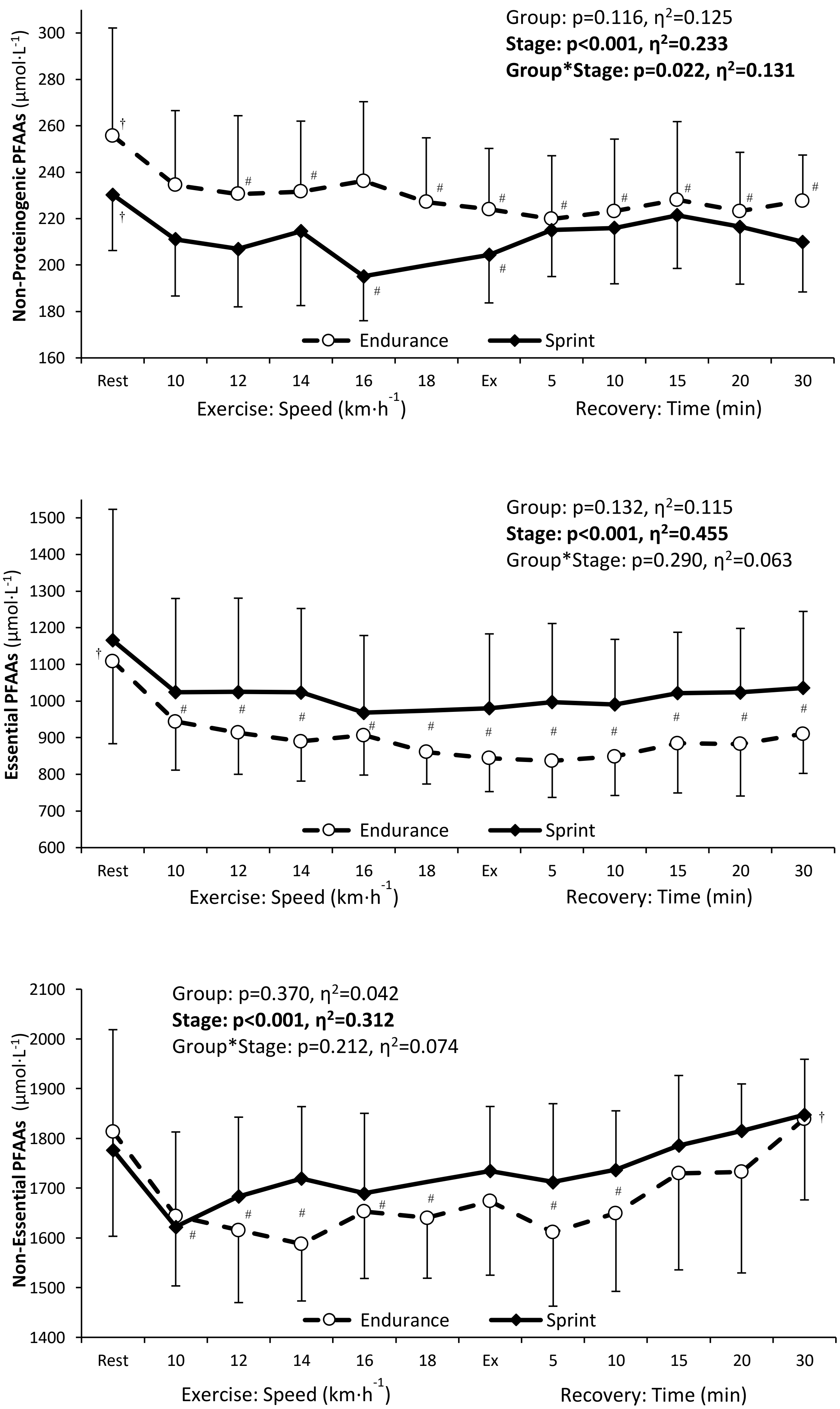
Group: p=0.116, η2=0.125
Stage: p<0.001, η2=0.233
Group*Stage: p=0.022, η2=0.131
Non-Proteinogenic PFAAs (µmol·L-1)
Endurance
Sprint
Rest
10
12
14
16
18
Ex
5
10
15
20
30
Exercise: Speed (km·h-1)
Recovery: Time (min)
Group: p=0.132, η2=0.115
Stage: p<0.001, η2=0.455
Group*Stage: p=0.290, η2=0.063
Essential PFAAs (µmol·L-1)
Group: p=0.370, η2=0.042
Stage: p<0.001, η2=0.312
Group*Stage: p=0.212, η2=0.074
Non-Essential PFAAs (µmol·L-1)

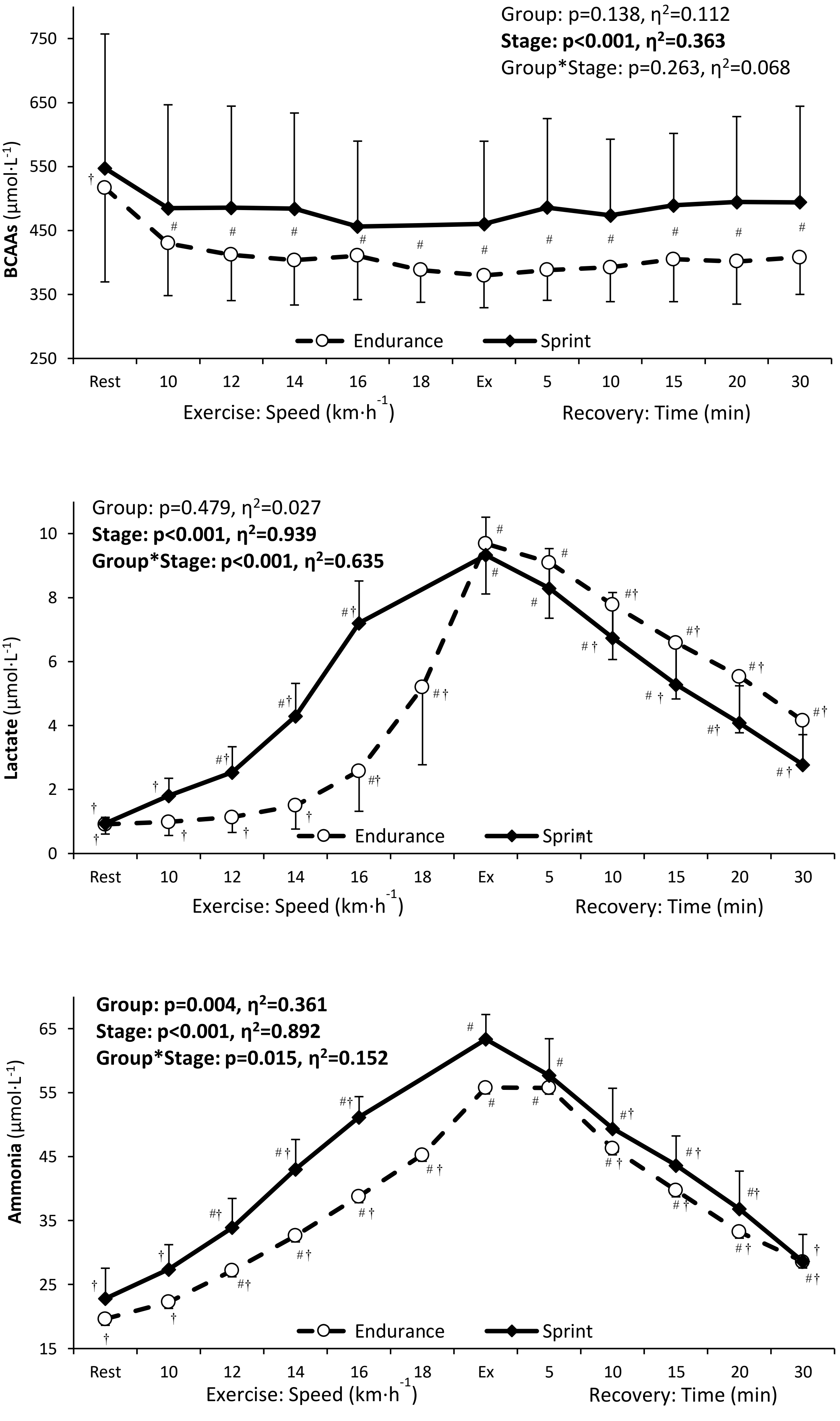
Group: p=0.138, η2=0.112
Stage: p<0.001, η2=0.363
Group*Stage: p=0.263, η2=0.068
BCAAs (µmol·L-1)
Endurance
Sprint
Rest
10
12
14
16
18
Ex
5
10
15
20
30
Exercise: Speed (km·h-1)
Recovery: Time (min)
Group: p=0.479, η2=0.027
Stage: p<0.001, η2=0.939
Group*Stage: p<0.001, η2=0.635
Lactate (µmol·L-1)
Endurance
Sprint
Exercise: Speed (km·h-1)
Recovery: Time (min)
Group: p=0.004, η2=0.361
Stage: p<0.001, η2=0.892
Group*Stage: p=0.015, η2=0.152
Ammonia (µmol·L-1)
Endurance
Sprint
Exercise: Speed (km·h-1)
Recovery: Time (min)
