# Supplementary material for: Exercise-induced response of proteinogenic and non-proteinogenic plasma free amino acids is sport-specific: A comparison of sprint and endurance athletes
Source: PLoS One. 2024 Aug 30;19(8):e0309529. doi: 10.1371/journal.pone.0309529 (PMC11364291; doi:10.1371/journal.pone.0309529)
Supplement: S2 Fig — (PDF) [file pone.0309529.s004.PDF]

S2 Figures The time course of the individual PFAAs concentrations (SMM-adjusted values) in endurance (open circles, dashed line) and speed-power (black squares, solid line) athletes at rest, during progressive exercise until exhaustion (Ex), and post-exercise recovery. For the full names of the amino acids, see the list of abbreviations in the main text.

Explanation: ANOVA main effects for the group (sports specialty), stage (exercise and recovery phase), and their interaction are shown in bold, if statistically significant. Bonferroni post hoc tests are denoted as follows: * different from the sprint-trained group at the same test stage (phase), # different from resting values, † different from values at exhaustion. If there was no group effect or interaction, post hoc test markings apply to both sports groups.

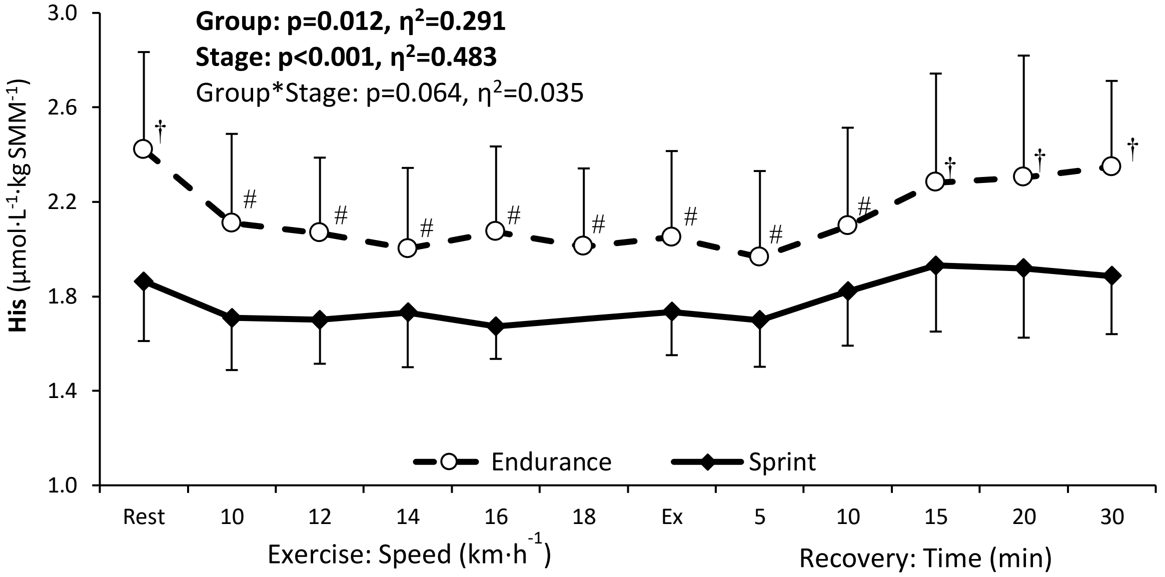

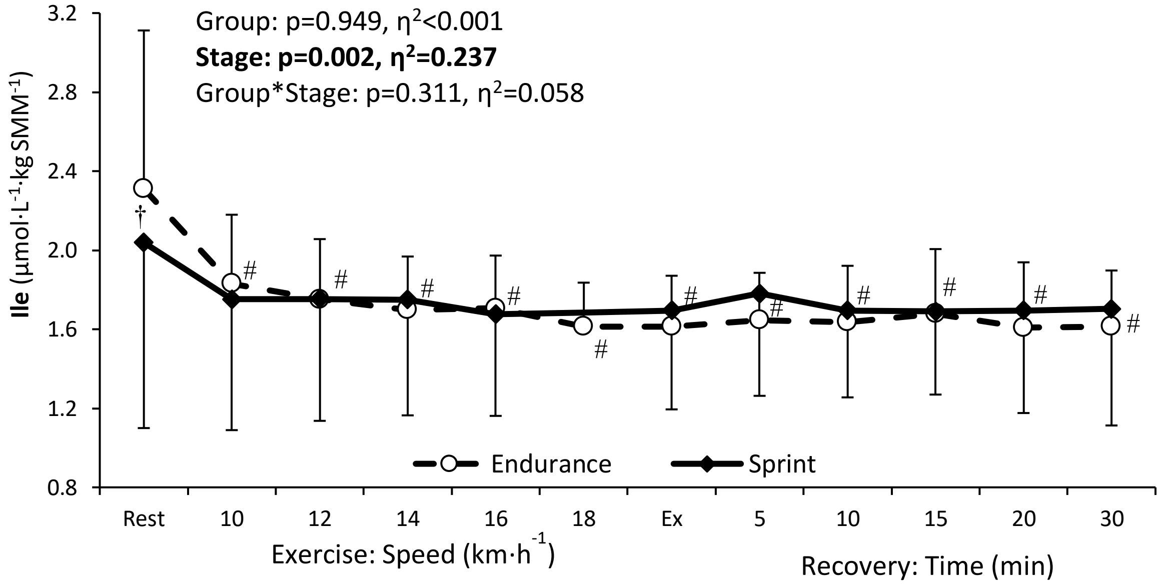

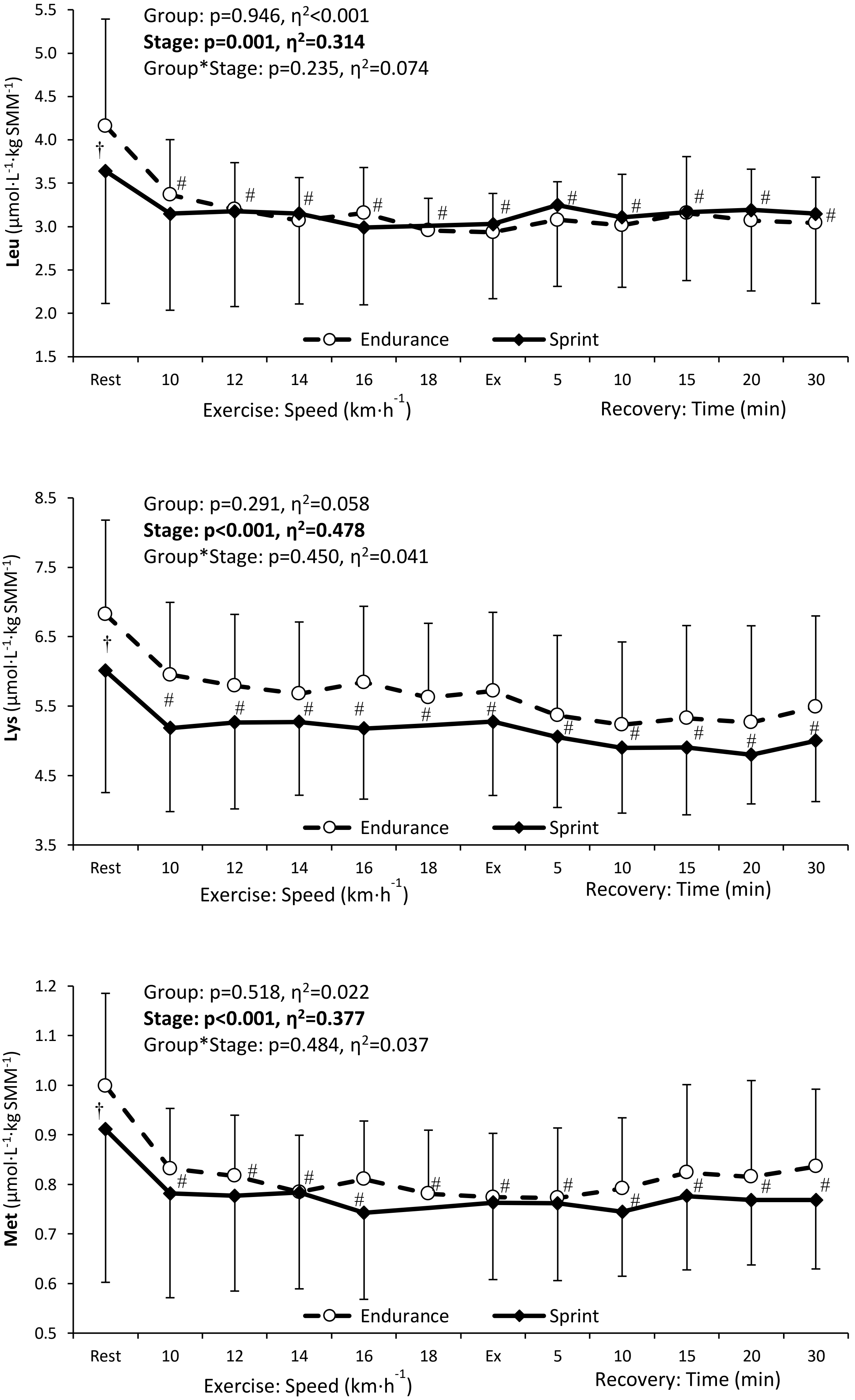

Group: p=0.946, η²<0.001
Stage: p=0.001, η²=0.314
Group*Stage: p=0.235, η²=0.074
Leu (µmol·L⁻¹·kg SMM⁻¹)
5.5
5.0
4.5
4.0
3.5
3.0
2.5
2.0
1.5
Endurance
Sprint
Rest
10
12
14
16
18
Ex
5
10
15
20
30
Exercise: Speed (km·h⁻¹)
Recovery: Time (min)
Group: p=0.291, η²=0.058
Stage: p<0.001, η²=0.478
Group*Stage: p=0.450, η²=0.041
Lys (µmol·L⁻¹·kg SMM⁻¹)
8.5
7.5
6.5
5.5
4.5
3.5
Group: p=0.518, η²=0.022
Stage: p<0.001, η²=0.377
Group*Stage: p=0.484, η²=0.037
Met (µmol·L⁻¹·kg SMM⁻¹)
1.2
1.1
1.0
0.9
0.8
0.7
0.6
0.5

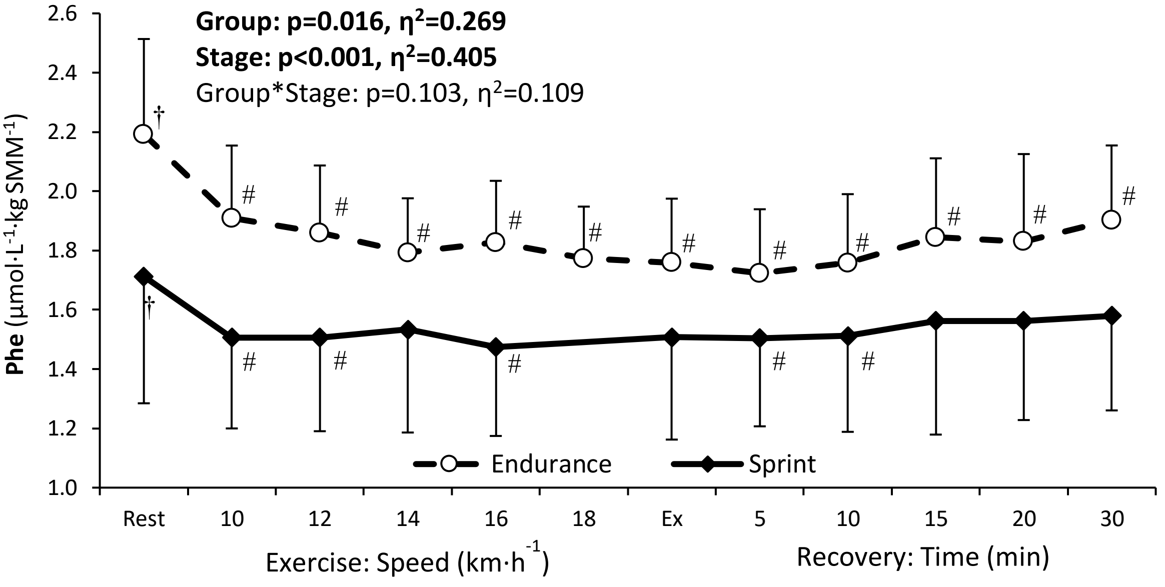
Group: p=0.016, η2=0.269
Stage: p<0.001, η2=0.405
Group*Stage: p=0.103, η2=0.109
Phe (µmol·L-1·kg SMM-1)
2.6
2.4
2.2
2.0
1.8
1.6
1.4
1.2
1.0
Endurance
Sprint
Rest
10
12
14
16
18
Ex
5
10
15
20
30
Exercise: Speed (km·h-1)
Recovery: Time (min)

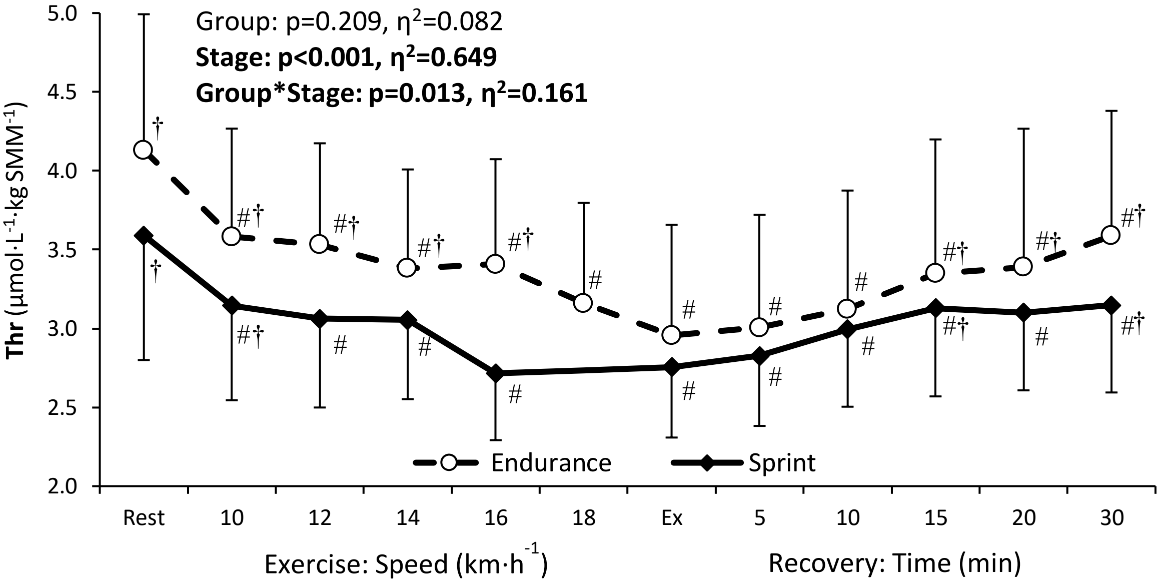
Group: p=0.209, η2=0.082
Stage: p<0.001, η2=0.649
Group*Stage: p=0.013, η2=0.161
Thr (µmol·L-1·kg SMM-1)
5.0
4.5
4.0
3.5
3.0
2.5
2.0
Endurance
Sprint
Rest
10
12
14
16
18
Ex
5
10
15
20
30
Exercise: Speed (km·h-1)
Recovery: Time (min)

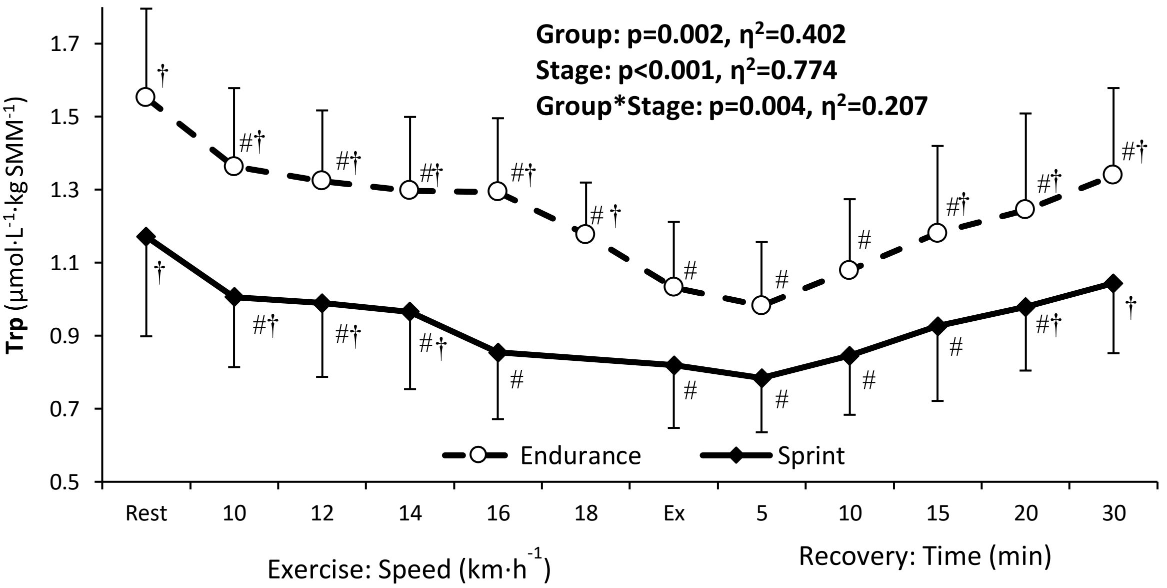
Group: p=0.002, η2=0.402
Stage: p<0.001, η2=0.774
Group*Stage: p=0.004, η2=0.207
Trp (µmol·L-1·kg SMM-1)
1.7
1.5
1.3
1.1
0.9
0.7
0.5
Endurance
Sprint
Rest
10
12
14
16
18
Ex
5
10
15
20
30
Exercise: Speed (km·h-1)
Recovery: Time (min)

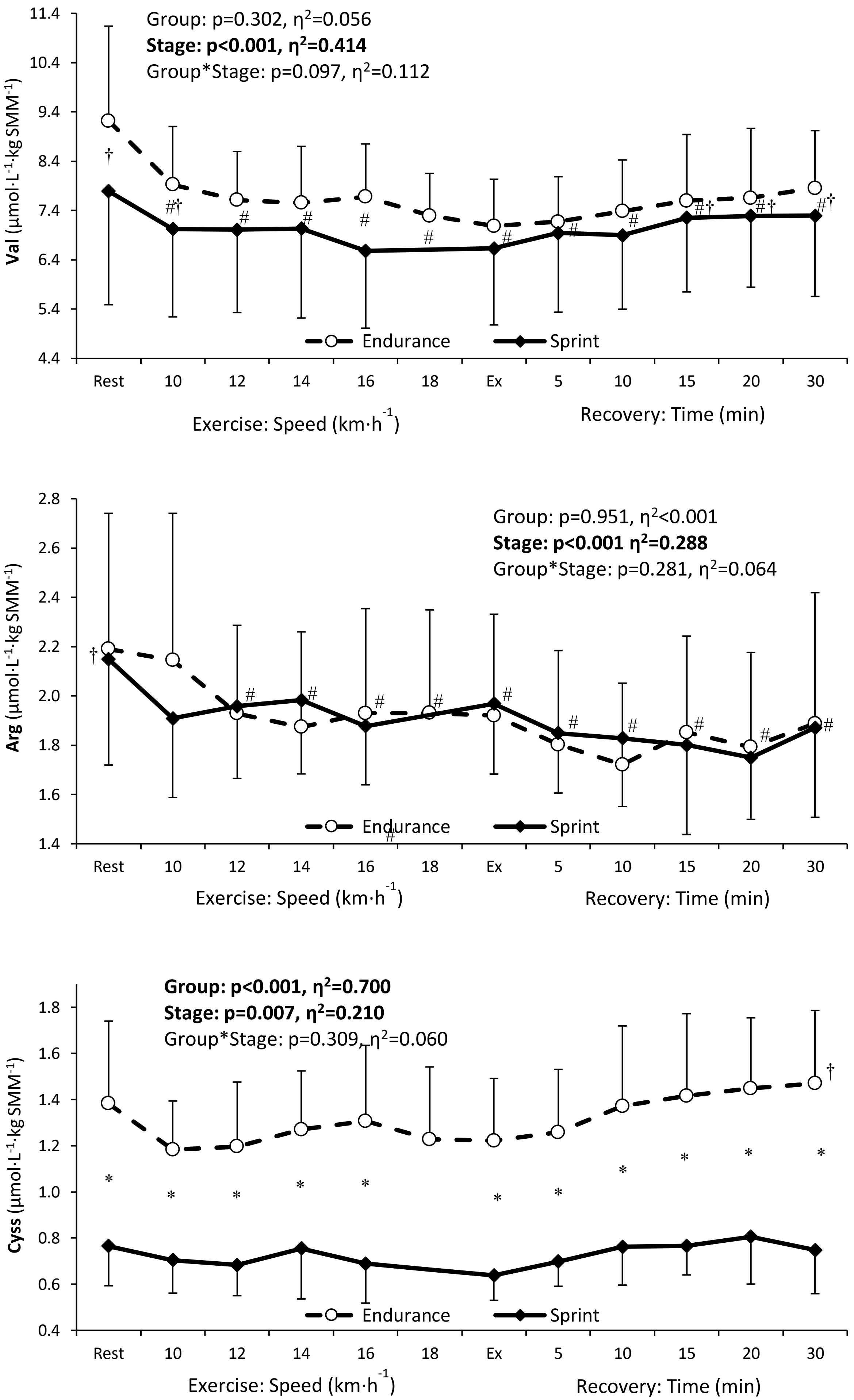

Group: p=0.302, η2=0.056
Stage: p<0.001, η2=0.414
Group*Stage: p=0.097, η2=0.112
Val (µmol·L-1·kg SMM-1)
Endurance
Sprint
Rest 10 12 14 16 18 Ex 5 10 15 20 30
Exercise: Speed (km·h-1)
Recovery: Time (min)
Group: p=0.951, η2<0.001
Stage: p<0.001 η2=0.288
Group*Stage: p=0.281, η2=0.064
Arg (µmol·L-1·kg SMM-1)
Endurance
Sprint
Rest 10 12 14 16 18 Ex 5 10 15 20 30
Exercise: Speed (km·h-1)
Recovery: Time (min)
Group: p<0.001, η2=0.700
Stage: p=0.007, η2=0.210
Group*Stage: p=0.309, η2=0.060
Cyss (µmol·L-1·kg SMM-1)
Endurance
Sprint
Rest 10 12 14 16 18 Ex 5 10 15 20 30
Exercise: Speed (km·h-1)
Recovery: Time (min)

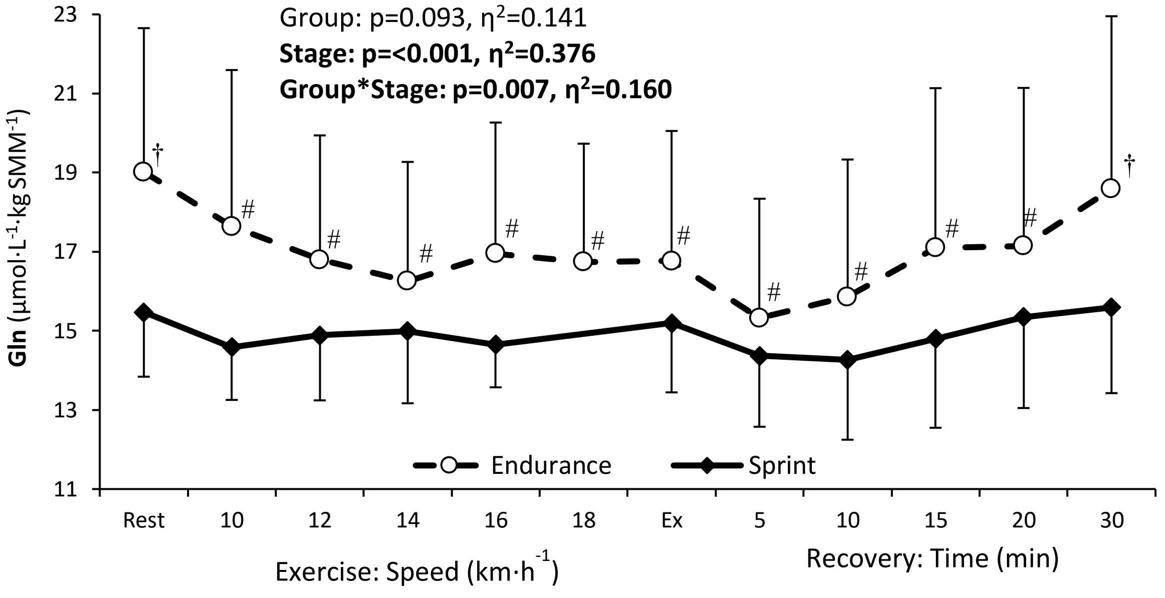
Group: p=0.093, η2=0.141
Stage: p=<0.001, η2=0.376
Group*Stage: p=0.007, η2=0.160
Gln (µmol·L-1·kg SMM-1)
Endurance
Sprint
Rest 10 12 14 16 18 Ex 5 10 15 20 30
Exercise: Speed (km·h-1)
Recovery: Time (min)

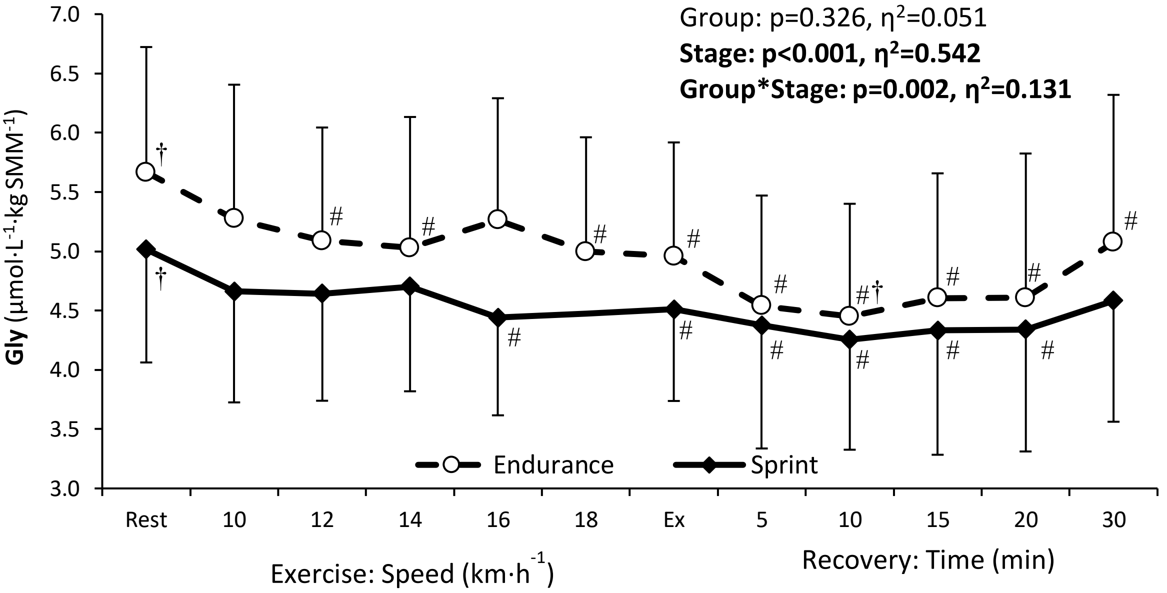
Group: p=0.326, η2=0.051
Stage: p<0.001, η2=0.542
Group*Stage: p=0.002, η2=0.131
Gly (µmol·L-1·kg SMM-1)
Endurance
Sprint
Rest 10 12 14 16 18 Ex 5 10 15 20 30
Exercise: Speed (km·h-1)
Recovery: Time (min)

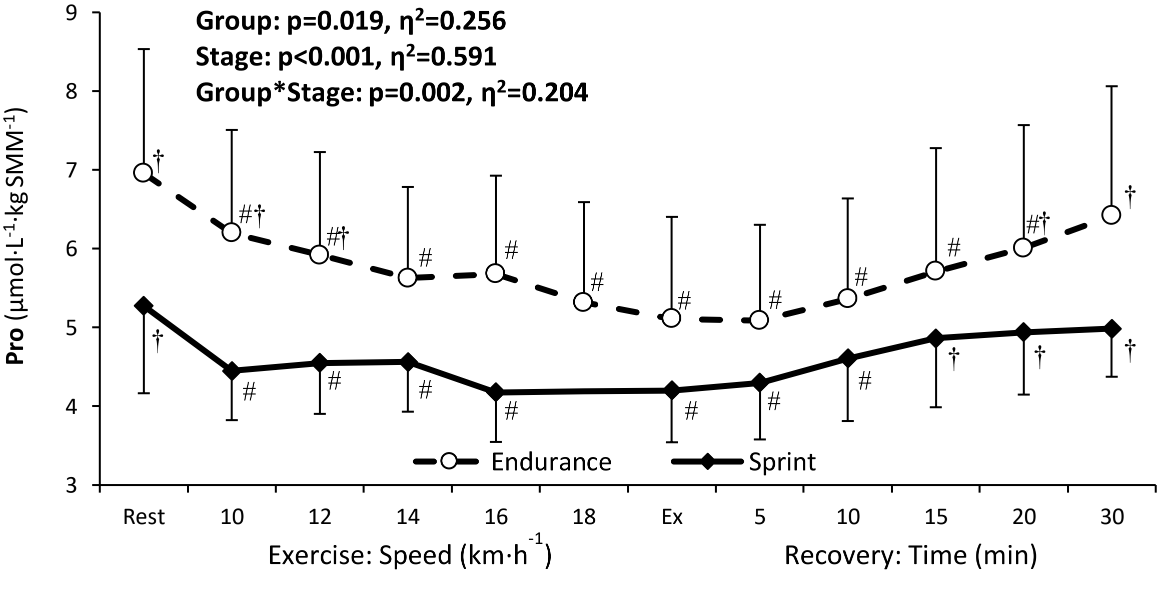
Group: p=0.019, η2=0.256
Stage: p<0.001, η2=0.591
Group*Stage: p=0.002, η2=0.204
Pro (µmol·L-1·kg SMM-1)
Endurance
Sprint
Rest 10 12 14 16 18 Ex 5 10 15 20 30
Exercise: Speed (km·h-1)
Recovery: Time (min)

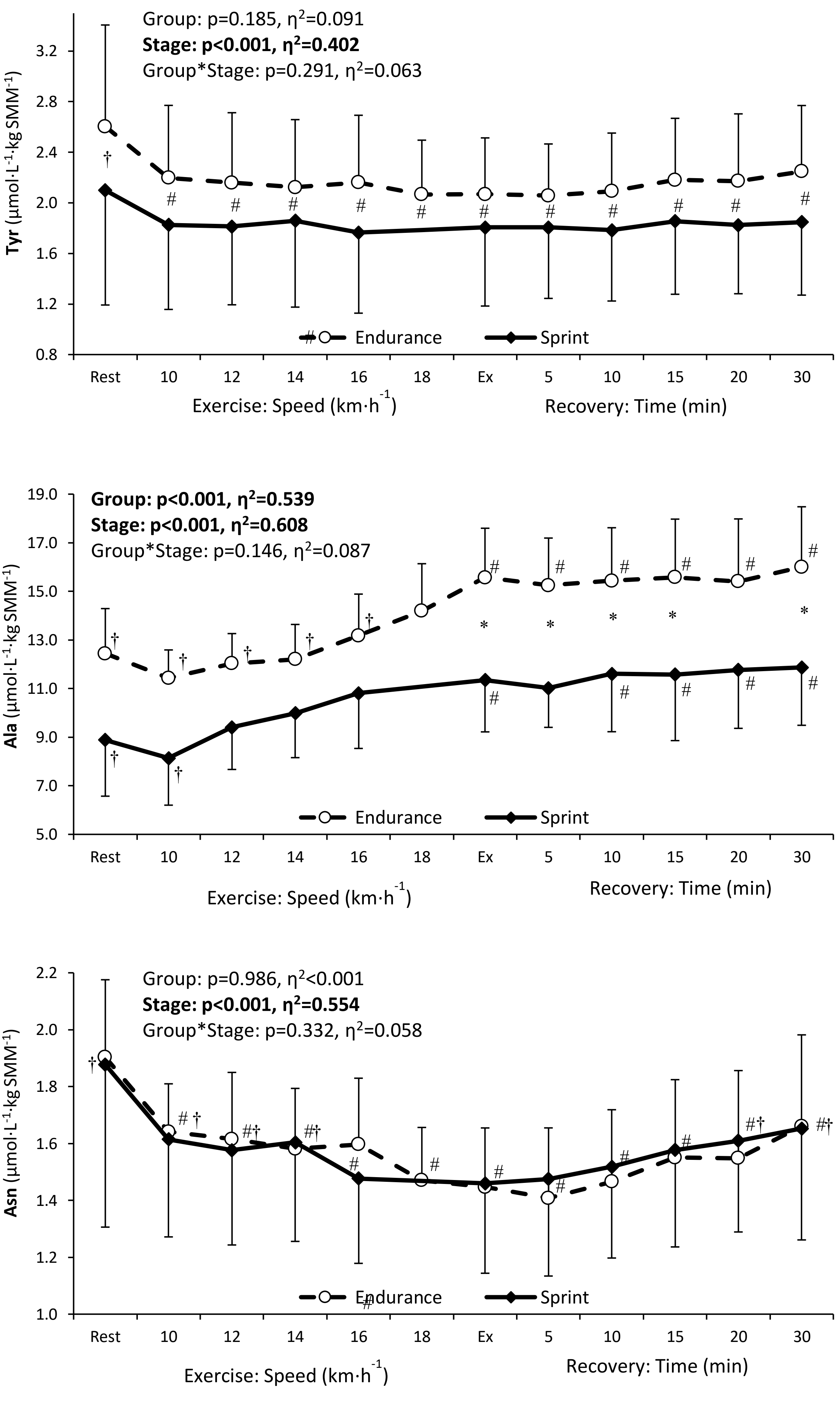

Group: p=0.185, η2=0.091
Stage: p<0.001, η2=0.402
Group*Stage: p=0.291, η2=0.063
Tyr (µmol·L-1·kg SMM-1)
Endurance
Sprint
Rest
10
12
14
16
18
Ex
5
10
15
20
30
Exercise: Speed (km·h-1)
Recovery: Time (min)
Group: p<0.001, η2=0.539
Stage: p<0.001, η2=0.608
Group*Stage: p=0.146, η2=0.087
Ala (µmol·L-1·kg SMM-1)
Group: p=0.986, η2<0.001
Stage: p<0.001, η2=0.554
Group*Stage: p=0.332, η2=0.058
Asn (µmol·L-1·kg SMM-1)

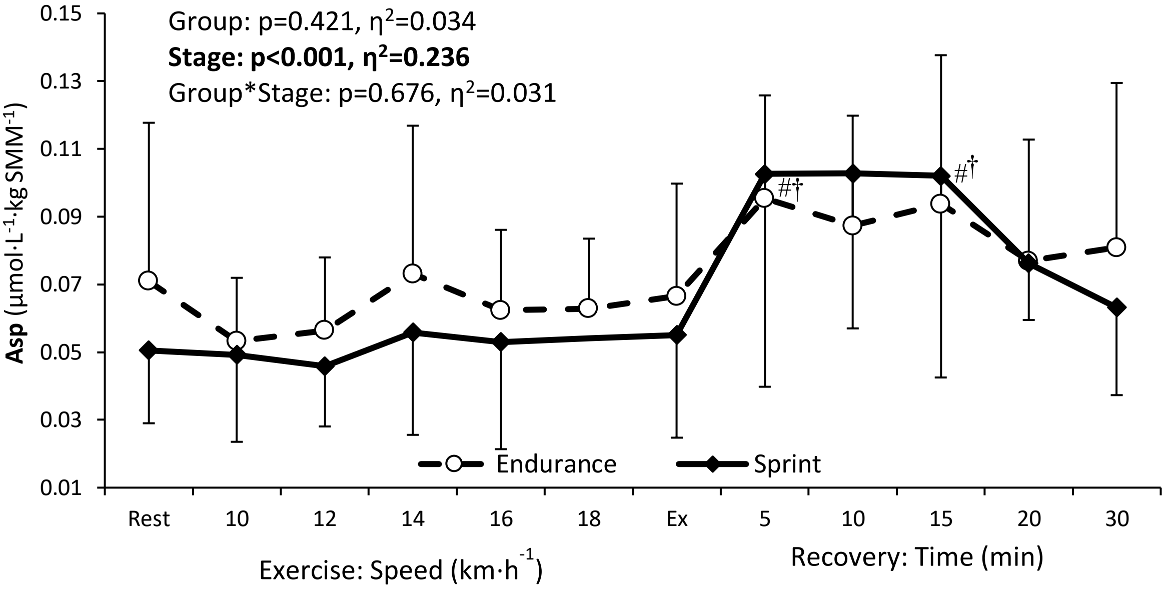
Group: p=0.421, η2=0.034
Stage: p<0.001, η2=0.236
Group*Stage: p=0.676, η2=0.031
Asp (µmol·L-1·kg SMM-1)
Endurance
Sprint
Rest 10 12 14 16 18 Ex 5 10 15 20 30
Exercise: Speed (km·h-1)
Recovery: Time (min)

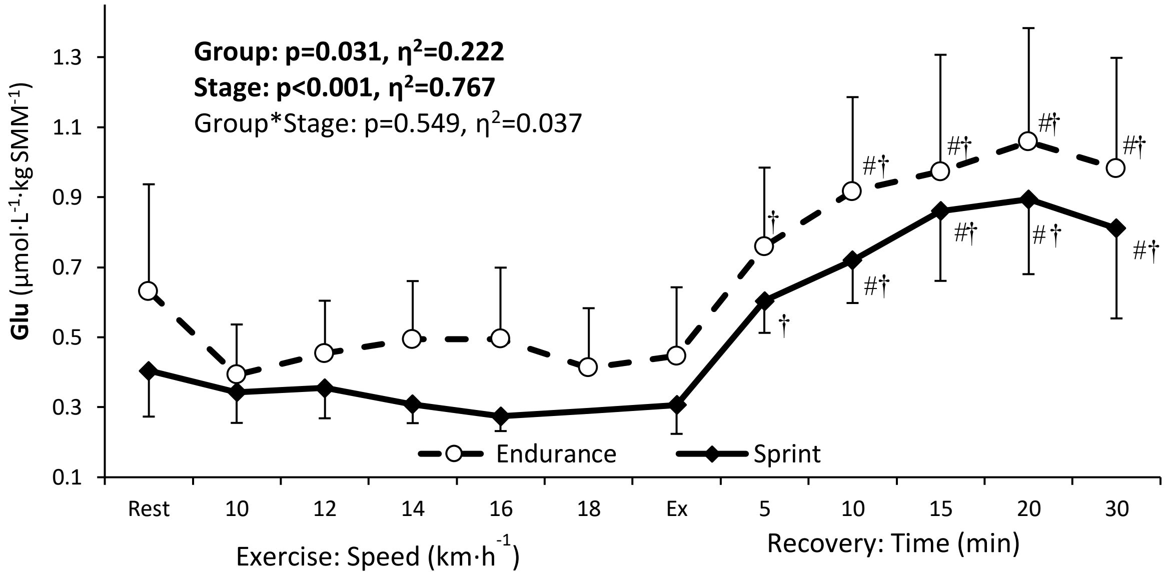
Group: p=0.031, η2=0.222
Stage: p<0.001, η2=0.767
Group*Stage: p=0.549, η2=0.037
Glu (µmol·L-1·kg SMM-1)
Endurance
Sprint
Rest 10 12 14 16 18 Ex 5 10 15 20 30
Exercise: Speed (km·h-1)
Recovery: Time (min)

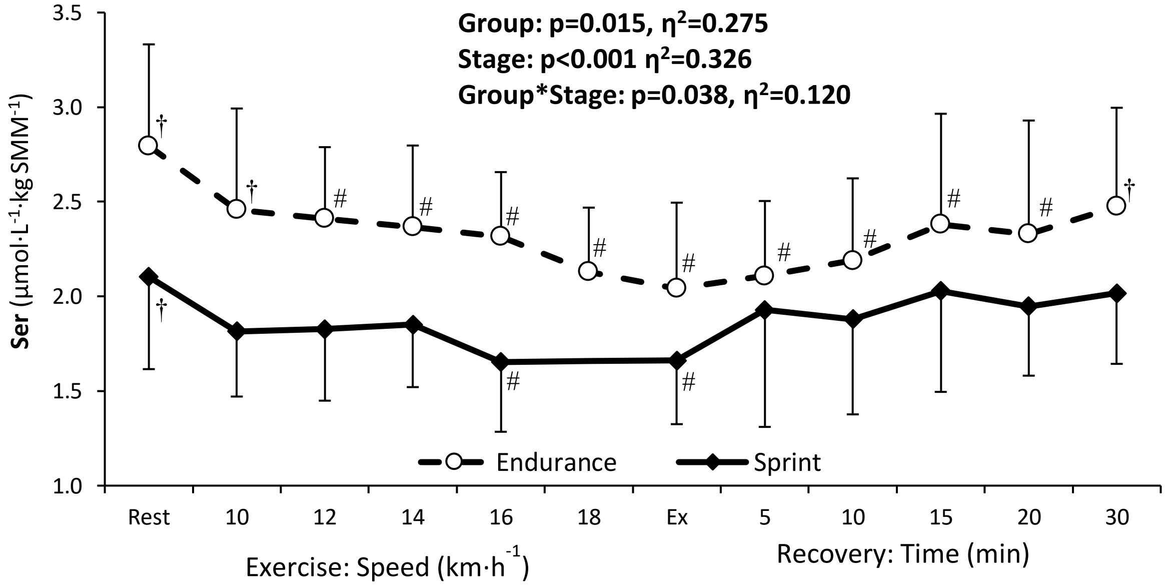
Group: p=0.015, η2=0.275
Stage: p<0.001 η2=0.326
Group*Stage: p=0.038, η2=0.120
Ser (µmol·L-1·kg SMM-1)
Endurance
Sprint
Rest 10 12 14 16 18 Ex 5 10 15 20 30
Exercise: Speed (km·h-1)
Recovery: Time (min)

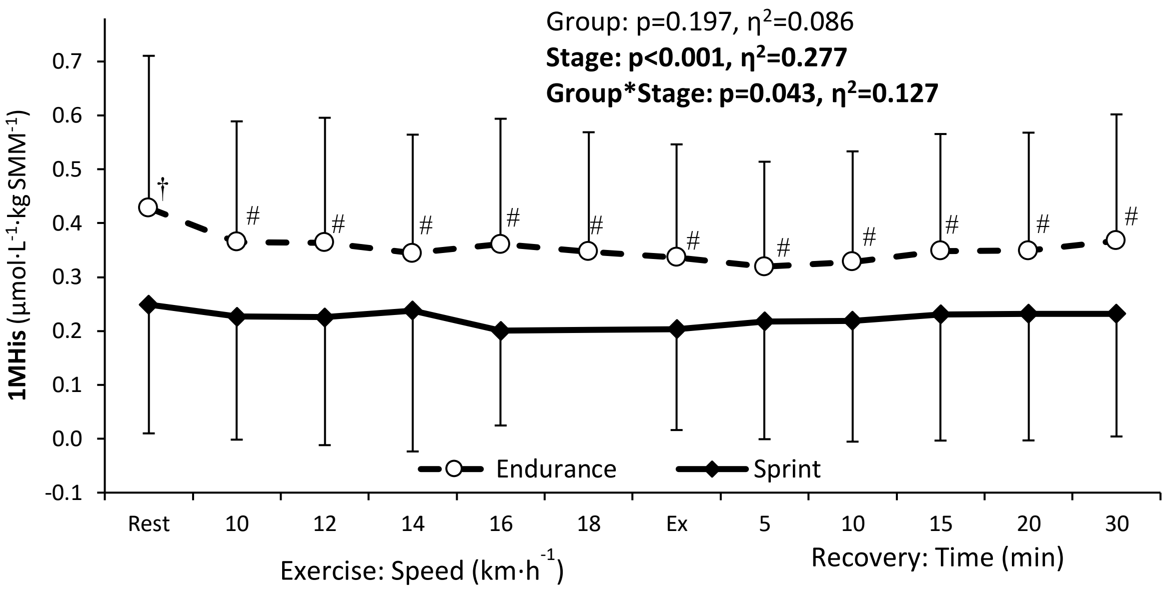

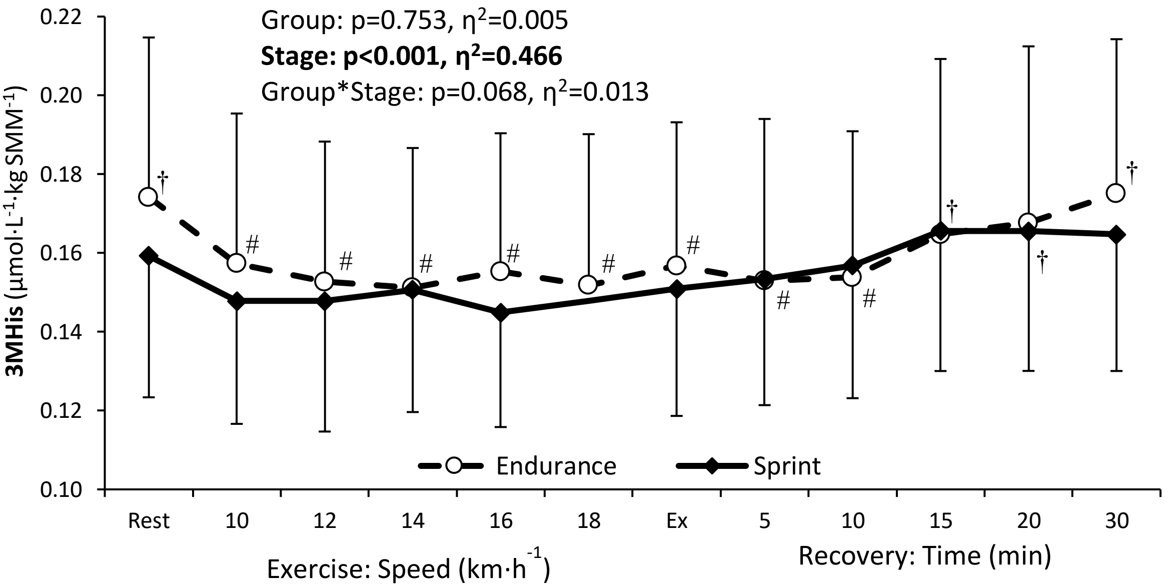

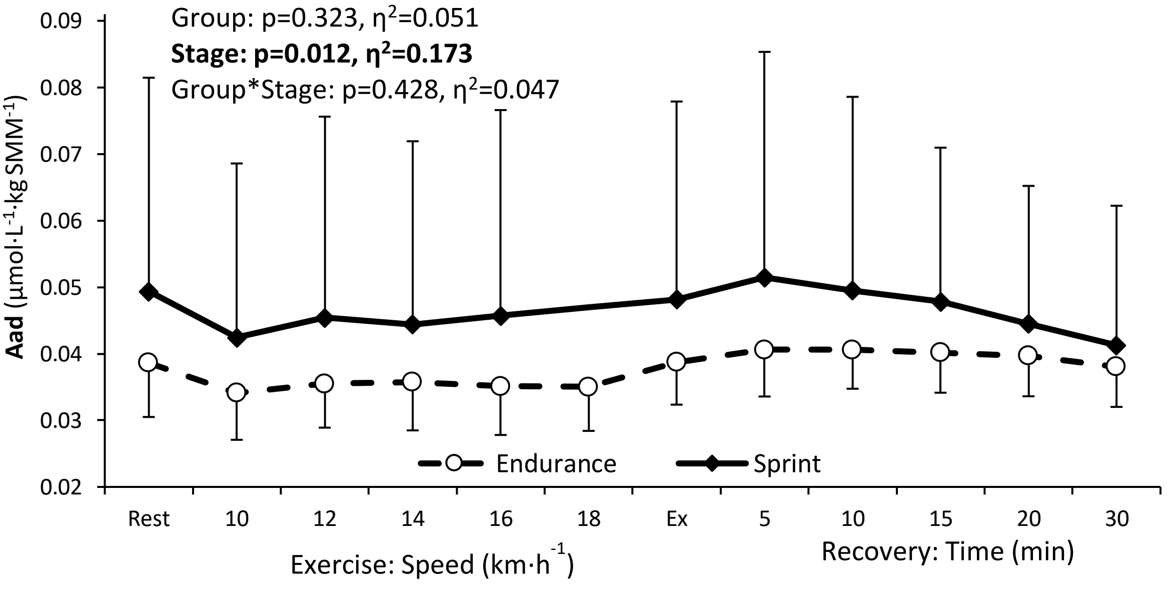

Aad: significant difference between exercise at10 km/h and 5−10th min of recovery; significant difference between 5th and 30th min of recovery

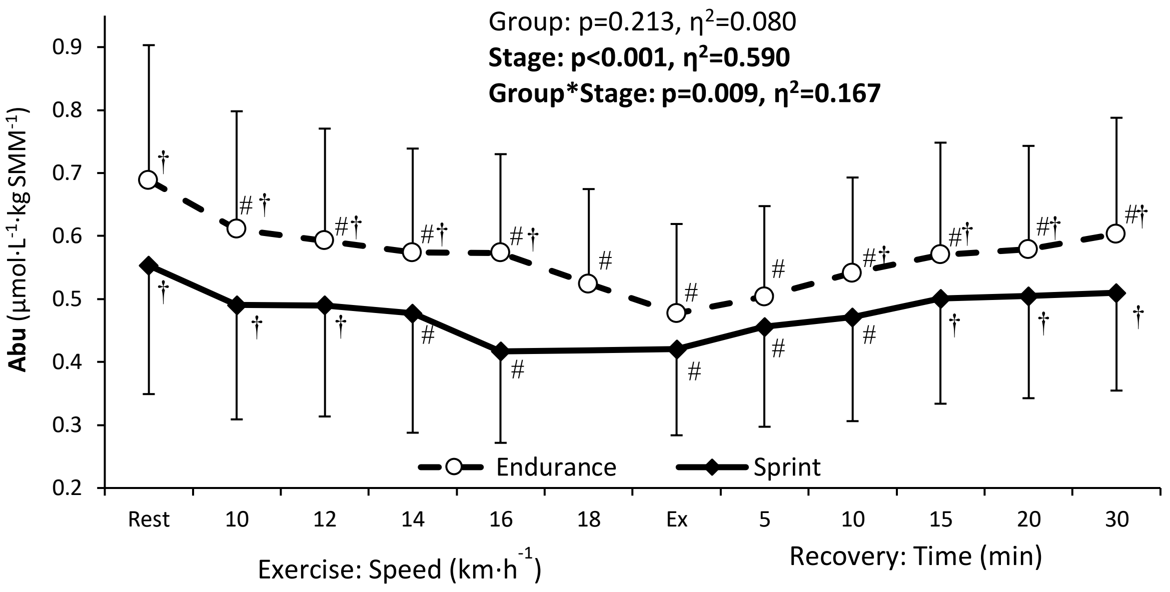

Group: p=0.213, η2=0.080
Stage: p<0.001, η2=0.590
Group*Stage: p=0.009, η2=0.167
Abu (µmol·L-1·kg SMM-1)
Endurance
Sprint
Rest 10 12 14 16 18 Ex 5 10 15 20 30
Exercise: Speed (km·h-1)
Recovery: Time (min)

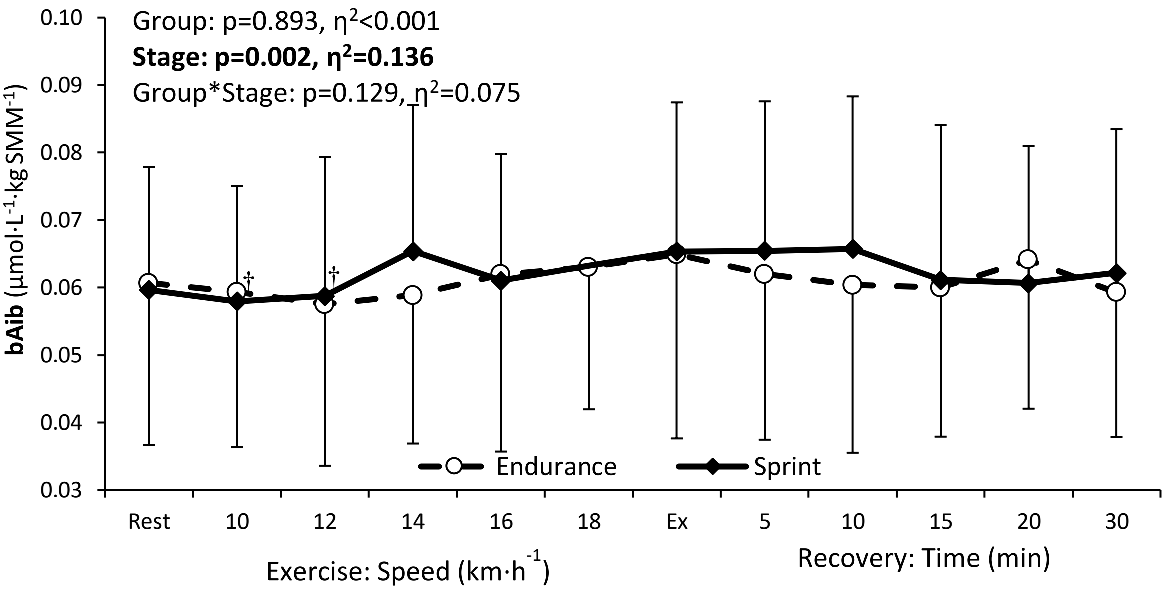

Group: p=0.893, η2<0.001
Stage: p=0.002, η2=0.136
Group*Stage: p=0.129, η2=0.075
bAib (µmol·L-1·kg SMM-1)
Endurance
Sprint
Rest 10 12 14 16 18 Ex 5 10 15 20 30
Exercise: Speed (km·h-1)
Recovery: Time (min)

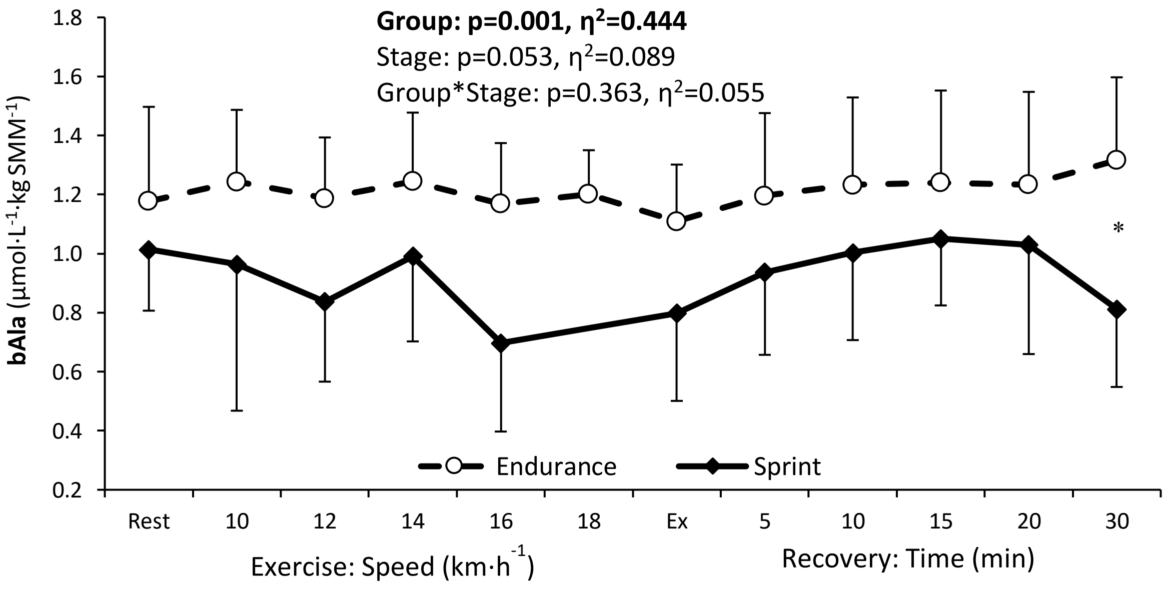

Group: p=0.001, η2=0.444
Stage: p=0.053, η2=0.089
Group*Stage: p=0.363, η2=0.055
bAla (µmol·L-1·kg SMM-1)
Endurance
Sprint
Rest 10 12 14 16 18 Ex 5 10 15 20 30
Exercise: Speed (km·h-1)
Recovery: Time (min)

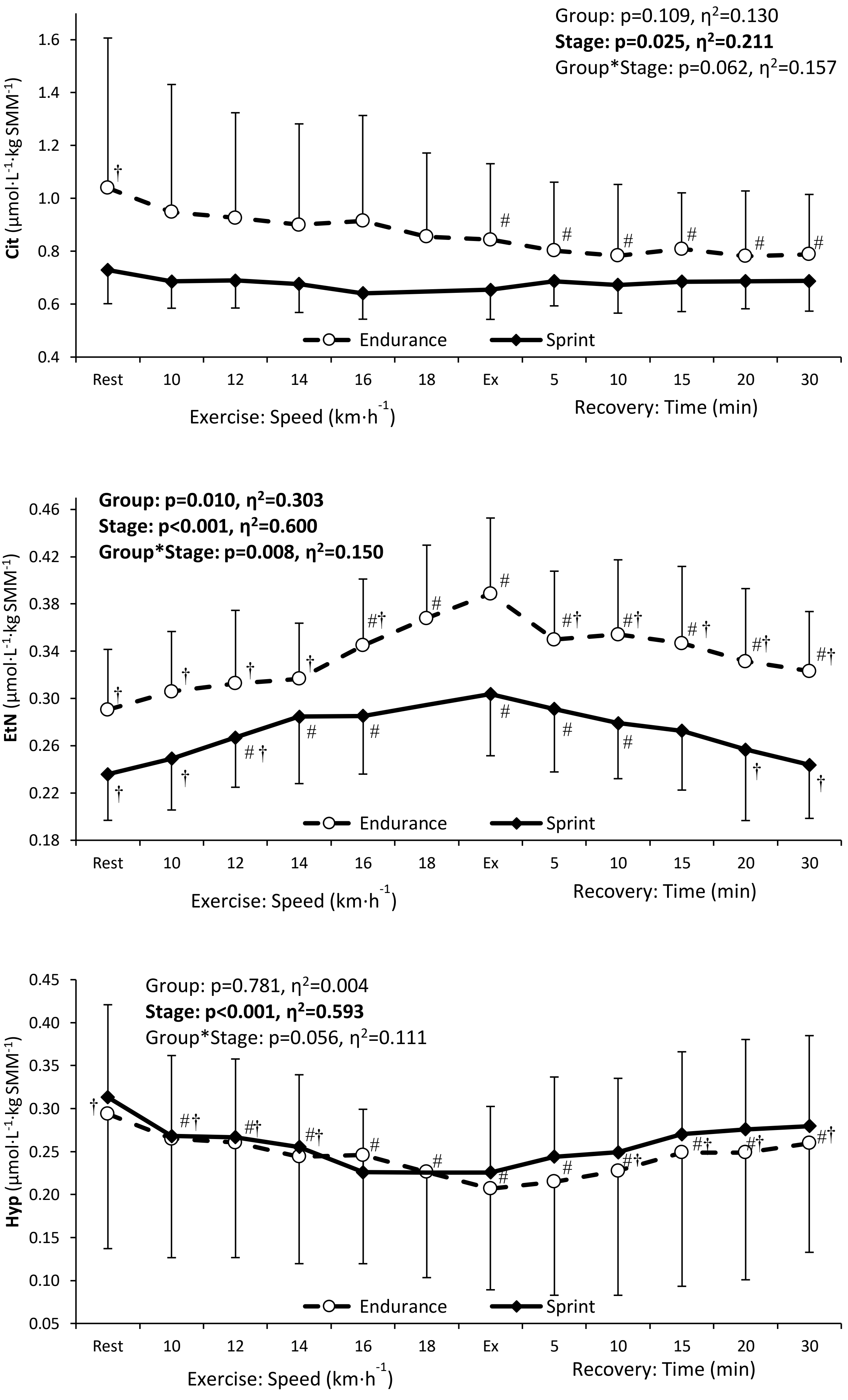
Group: p=0.109, η2=0.130
Stage: p=0.025, η2=0.211
Group*Stage: p=0.062, η2=0.157
Cit (µmol·L-1·kg SMM-1)
Endurance
Sprint
Rest
10
12
14
16
18
Ex
5
10
15
20
30
Exercise: Speed (km·h-1)
Recovery: Time (min)
Group: p=0.010, η2=0.303
Stage: p<0.001, η2=0.600
Group*Stage: p=0.008, η2=0.150
EtN (µmol·L-1·kg SMM-1)
Group: p=0.781, η2=0.004
Stage: p<0.001, η2=0.593
Group*Stage: p=0.056, η2=0.111
Hyp (µmol·L-1·kg SMM-1)

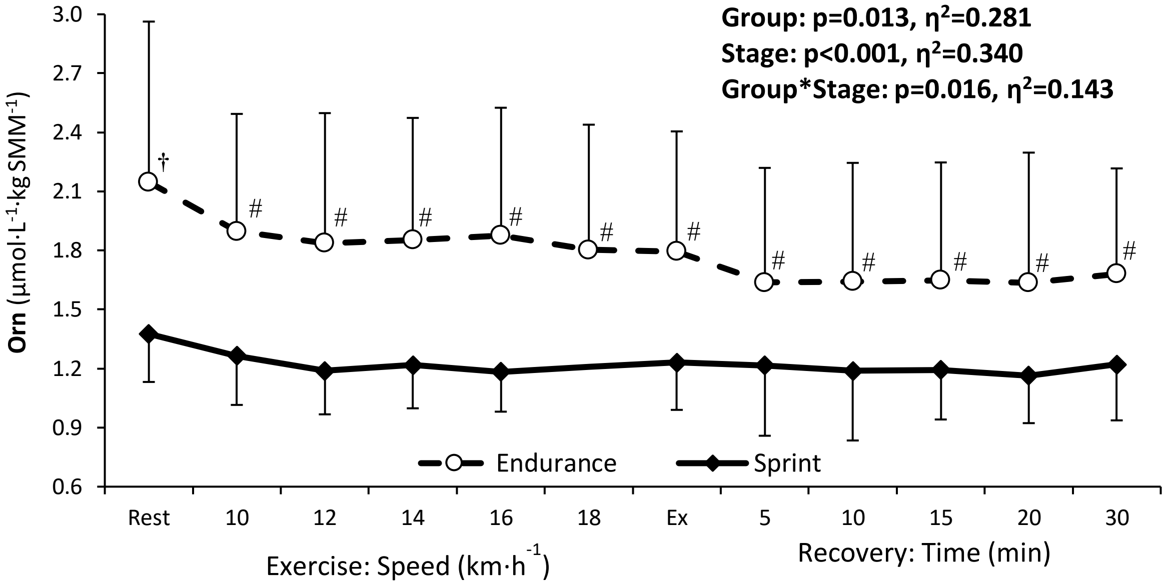

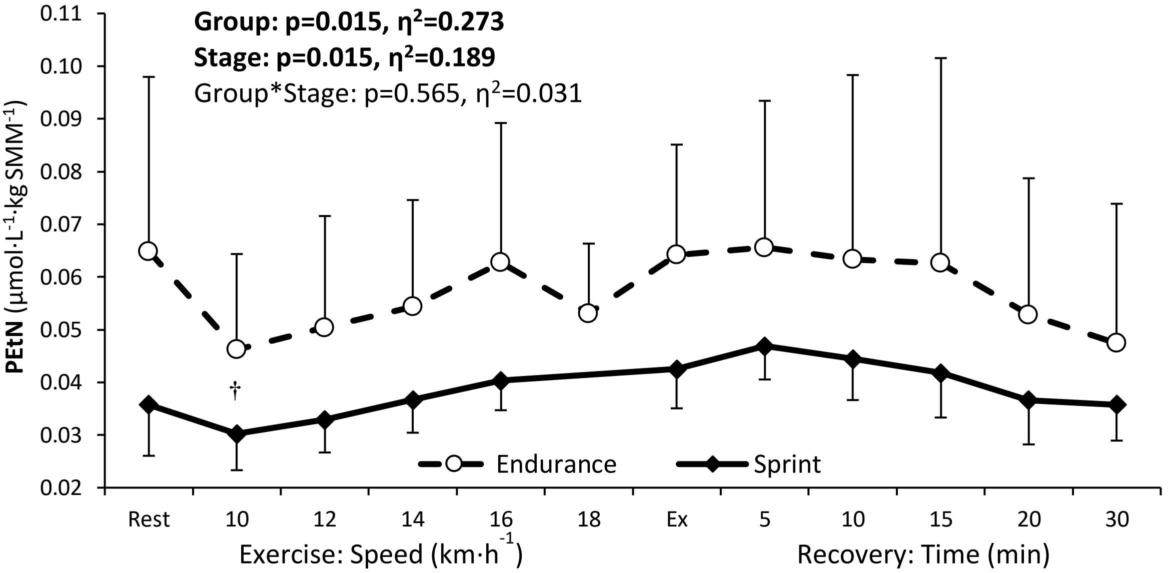

PEtN: significant difference between exercise at10 km/h and 5−15th min of recovery; significant difference between 5th and 30th min of recovery

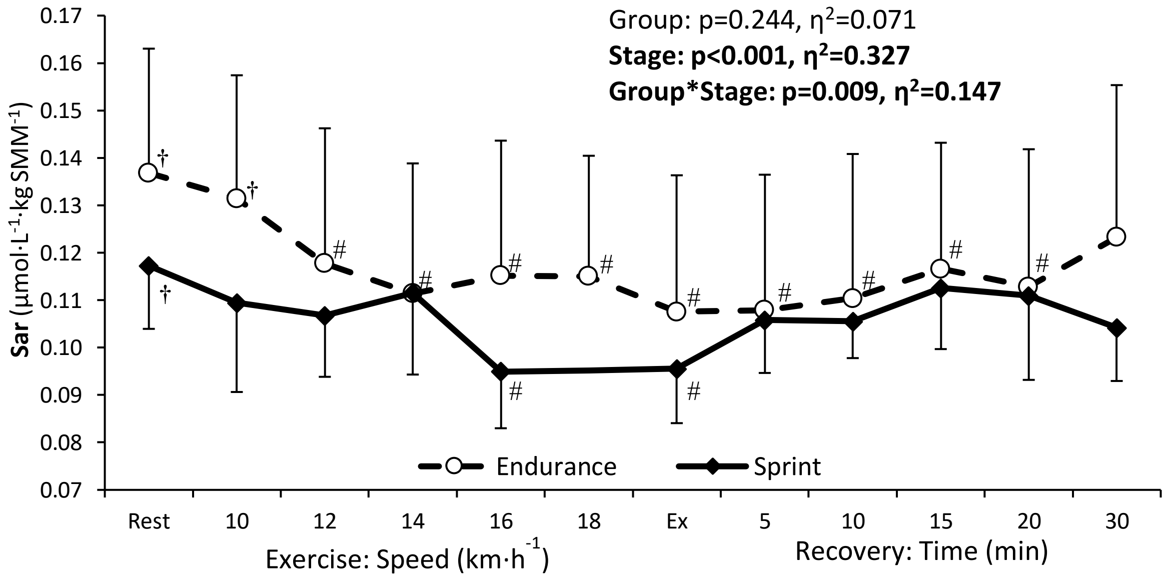

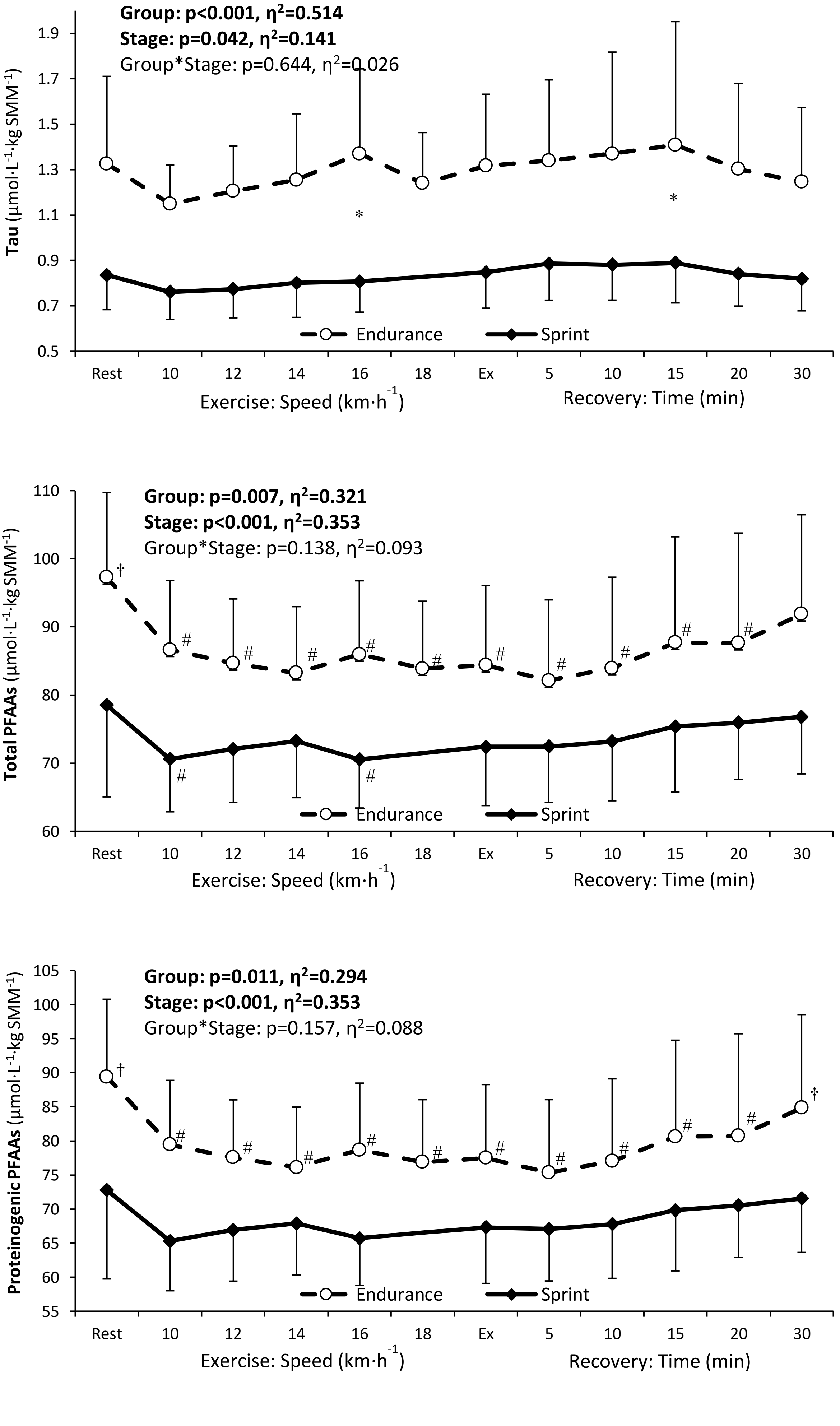
Group: p<0.001, η2=0.514
Stage: p=0.042, η2=0.141
Group*Stage: p=0.644, η2=0.026
Tau (μmol·L-1·kg SMM-1)
1.9
1.7
1.5
1.3
1.1
0.9
0.7
0.5
*
*
Endurance
Sprint
Rest
10
12
14
16
18
Ex
5
10
15
20
30
Exercise: Speed (km·h-1)
Recovery: Time (min)
Group: p=0.007, η2=0.321
Stage: p<0.001, η2=0.353
Group*Stage: p=0.138, η2=0.093
Total PFAAs (μmol·L-1·kg SMM-1)
110
100
90
80
70
60
†
#
Endurance
Sprint
Rest
10
12
14
16
18
Ex
5
10
15
20
30
Exercise: Speed (km·h-1)
Recovery: Time (min)
Group: p=0.011, η2=0.294
Stage: p<0.001, η2=0.353
Group*Stage: p=0.157, η2=0.088
Proteinogenic PFAAs (μmol·L-1·kg SMM-1)
105
100
95
90
85
80
75
70
65
60
55
†
#
Endurance
Sprint
Rest
10
12
14
16
18
Ex
5
10
15
20
30
Exercise: Speed (km·h-1)
Recovery: Time (min)

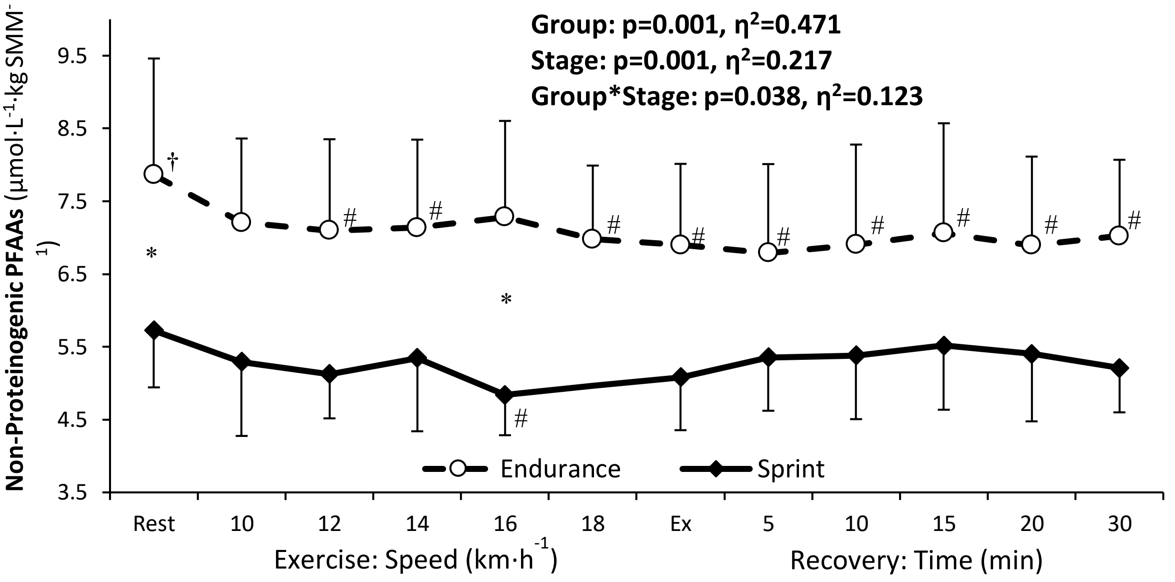
Group: p=0.001, η2=0.471
Stage: p=0.001, η2=0.217
Group*Stage: p=0.038, η2=0.123
Non-Proteinogenic PFAAs (µmol·L-1·kg SMM-1)
Endurance
Sprint
Rest 10 12 14 16 18 Ex 5 10 15 20 30
Exercise: Speed (km·h-1)
Recovery: Time (min)

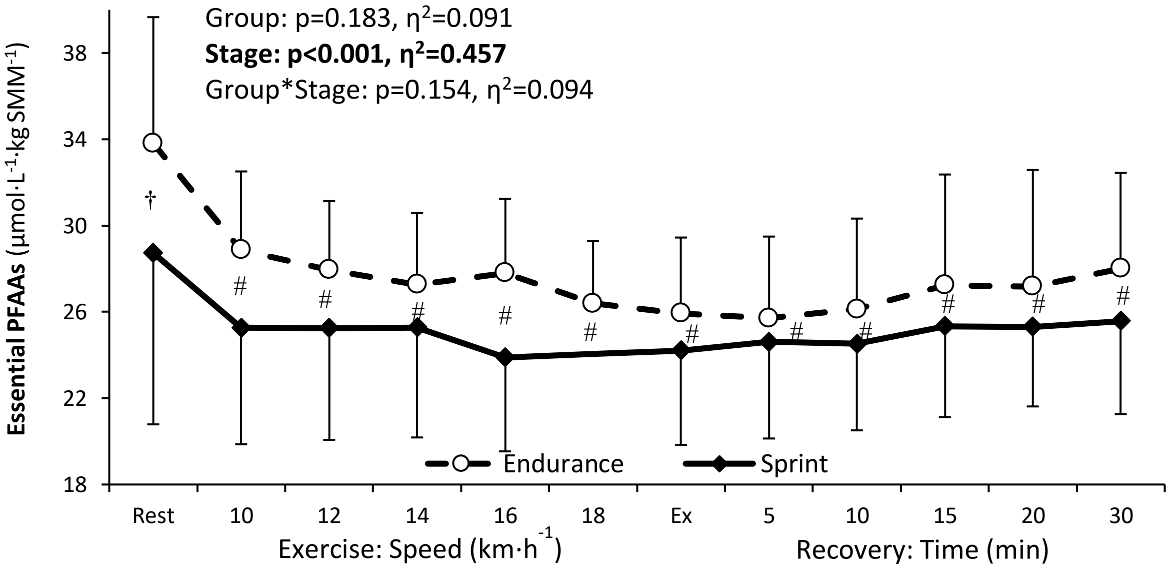
Group: p=0.183, η2=0.091
Stage: p<0.001, η2=0.457
Group*Stage: p=0.154, η2=0.094
Essential PFAAs (µmol·L-1·kg SMM-1)
Endurance
Sprint
Rest 10 12 14 16 18 Ex 5 10 15 20 30
Exercise: Speed (km·h-1)
Recovery: Time (min)

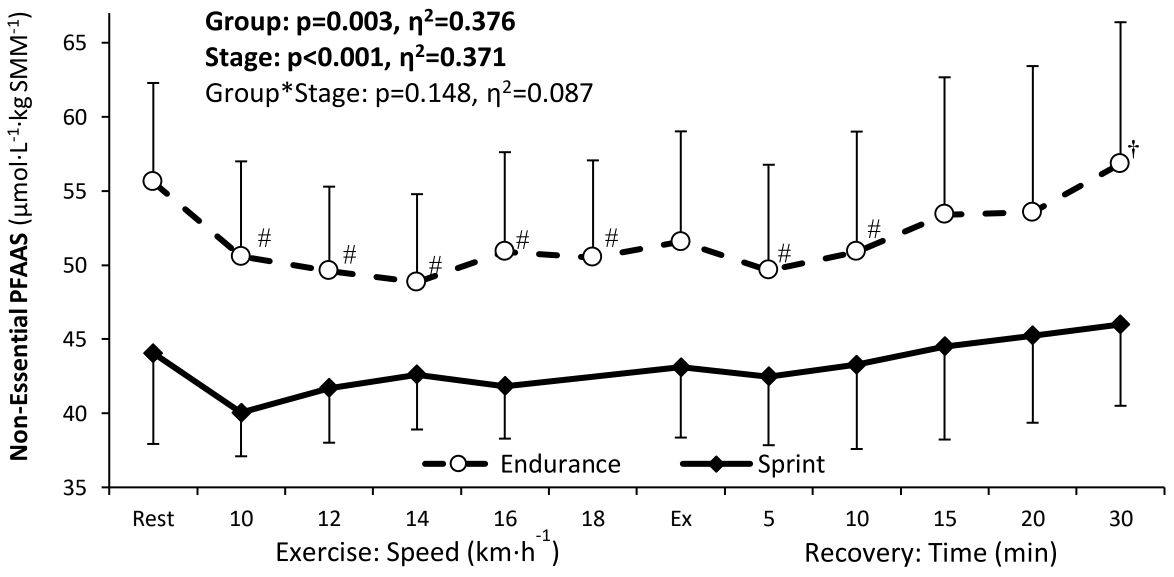
Group: p=0.003, η2=0.376
Stage: p<0.001, η2=0.371
Group*Stage: p=0.148, η2=0.087
Non-Essential PFAAS (µmol·L-1·kg SMM-1)
Endurance
Sprint
Rest 10 12 14 16 18 Ex 5 10 15 20 30
Exercise: Speed (km·h-1)
Recovery: Time (min)

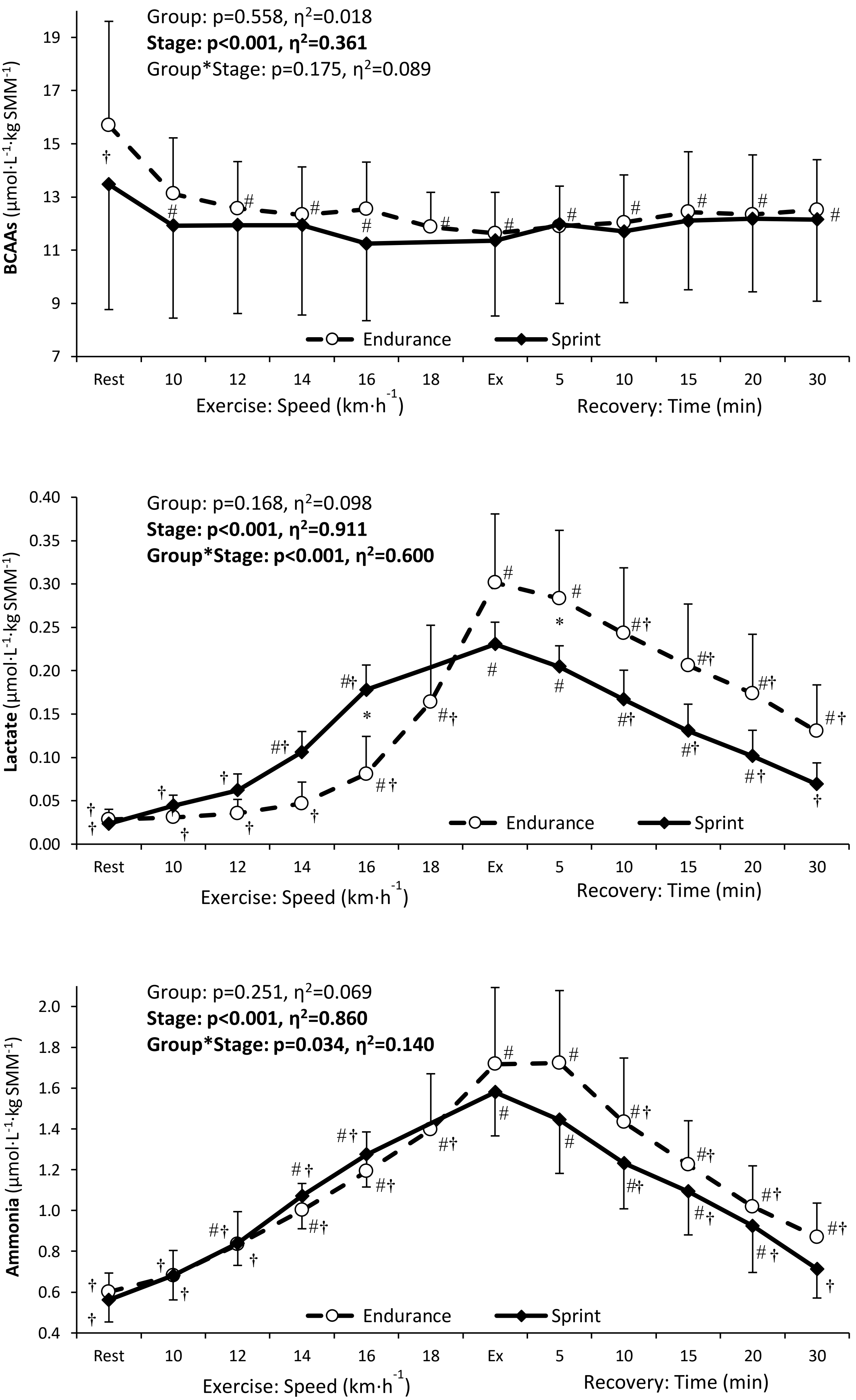

Group: p=0.558, η2=0.018
Stage: p<0.001, η2=0.361
Group*Stage: p=0.175, η2=0.089
BCAAs (µmol·L-1·kg SMM-1)
Endurance
Sprint
Rest 10 12 14 16 18 Ex 5 10 15 20 30
Exercise: Speed (km·h-1)
Recovery: Time (min)
Group: p=0.168, η2=0.098
Stage: p<0.001, η2=0.911
Group*Stage: p<0.001, η2=0.600
Lactate (µmol·L-1·kg SMM-1)
Endurance
Sprint
Rest 10 12 14 16 18 Ex 5 10 15 20 30
Exercise: Speed (km·h-1)
Recovery: Time (min)
Group: p=0.251, η2=0.069
Stage: p<0.001, η2=0.860
Group*Stage: p=0.034, η2=0.140
Ammonia (µmol·L-1·kg SMM-1)
Endurance
Sprint
Rest 10 12 14 16 18 Ex 5 10 15 20 30
Exercise: Speed (km·h-1)
Recovery: Time (min)
