# Supplementary material for: Exercise-induced response of proteinogenic and non-proteinogenic plasma free amino acids is sport-specific: A comparison of sprint and endurance athletes
Source: PLoS One. 2024 Aug 30;19(8):e0309529. doi: 10.1371/journal.pone.0309529 (PMC11364291; doi:10.1371/journal.pone.0309529)
Supplement: Attachment — Submitted filename: Review.docx [file pone.0309529.s005.docx]

The publication titled “Exercise-induced response of proteinogenic and non-proteinogenic plasma free amino acids is sport-specific: A comparison of sprint and endurance athletes” is a significant study examining how exercise affect the levels of proteinogenic and non-proteinogenic plasma free amino acids. I recommend that the editor to accept this manuscript after a minor revision addressing the following concerns:

1. The supporting information is missing from the original file. The author should upload the supporting information when submitting the revision.
2. The author should consider arranging the figures horizontally instead of vertically.
