# Supplementary material for: Exercise-induced response of proteinogenic and non-proteinogenic plasma free amino acids is sport-specific: A comparison of sprint and endurance athletes
Source: PLoS One. 2024 Aug 30;19(8):e0309529. doi: 10.1371/journal.pone.0309529 (PMC11364291; doi:10.1371/journal.pone.0309529)
Supplement: Attachment — Submitted filename: 0-Response to Reviews.docx [file pone.0309529.s006.docx]

PONE-D-24-00565

Exercise-induced response of proteinogenic and non-proteinogenic plasma free amino acids is sport-specific: A comparison of sprint and endurance athletes

PLOS ONE

**Reviewer #1:** The manuscript is about comparison of exercise-induced response of proteinogenic and non-proteinogenic plasma free amino acids (PFAAs) between individuals with opposite metabolic adaptations to athletic training (sprint versus endurance athletes).

Although this topic was addressed relatively frequently in the 1990s, the authors of the present study applied a valuable methodological solution based on robust methods and procedures for PFAA determination and analysis (i.e., multiple blood draws and expression of concentrations relative to SMM). Unfortunately, the available literature mainly provides limited pre- and post-exercise data, ignoring the course of PFAA changes during progressive exercise.

Thank you for your overall positive assessment. We realize, there are many studies dealing with various aspects of exercise-induced amino acid metabolism. We are trying to complete the picture by analyzing proteinogenic and non-proteinogenic amino acids at the same time, taking blood samples more frequently, and, most importantly, directly comparing in a single study individuals with extremely different metabolic adaptations (such attempts have not been made to our knowledge).

Abbreviations and units

I would recommend writing the maximum oxygen uptake as „V̇O2max” not as „V̇O2max”. Please change throughout the text.

Thank you for your comment. There are simplified versions of the abbreviation for maximal oxygen consumption used in various scientific and popular texts. We are not sure about the Reviewer’s intention, as both abbreviations in the above comment are written identically. Dot above V is obvious (volumetric flow) and this is the notation we used. The ‘2’ should be and is in subscript, so we think it is about "max", which we in the manuscript write as subscript. In fact, writing "max" in the normal (base)line of type may be more correct, so we changed the subscript “max” to normal line. We have also consistently corrected the abbreviations for HRmax, NH_3_max, and LAmax.

When it refers to heart rate (HR) I would recommend using „b·min-1” rather than „bpm”. Please change throughout the text

Thank you. Corrected.

Participants

Lines 76-78: Although the authors provide the inclusion criteria for the training experience of the participants (i.e. „involvement in a planned and structured training process for at least 5 years”), it is worthwhile to provide accurate data on the training age/experience of both groups. The authors can include this information in Table 1.

Thank you for indicating this detail. The data have been supplemented in Table 1 as years of ‘specialized training’.

Study design

In general, the control of participants' diet is the biggest limitation of the study (especially protein supplementation), but for objective reasons it is very difficult to precisely control this issue within the implemented study model.

We know that environmental factors like diet (e.g. protein-rich diet in strength athletes), dietary supplement intake (e.g. amino acid supplements), medication and exercise training (e.g. muscle damage) should be recorded and ideally standardized as they can influence metabolite concentrations in blood.

At least an estimate of what the protein intake per kilogram of body weight was would have been useful. This is important in the context of the results obtained, because, for example chronic arginine aspartate supplementation in runners reduces total PFAA level at rest and during a marathon run. Moreover, the speed and strength training period strongly decreases the fasting concentrations of amino acids in the power-trained athletes in a good anabolic state with the daily protein intake of 1.26 g·kg-1 body weight. At the same time the intensive lactic exercise session induces strong decreases, especially in valine, asparagine, and taurine.

Practically, on the day before testing, protein intake should have been standardized to 20% of total macronutrient intake.

Thank you for this crucial comment. We fully agree on the relevance of the factors mentioned by the reviewer that affect the concentration of amino acids in the blood. In our study, we can exclude the influence of medication, since the athletes studied were healthy and were not treated for any chronic or acute diseases. Sports training specificity was, by design, radically different in the two groups (that's why we compared them), thus the "standardization" of training could only consist in the cessation of strenuous high-intensity and/or long-duration training sessions 24‒48 hours before the laboratory visit in order to keep the athletes sufficiently rested and recovered (to which the coaches and athletes willingly adapted, since they were motivated for obtaining the best possible test performance and diagnose their cardiorespiratory and body composition parameters to support training control and planning). Please note that the average creatine kinase levels (CK; a common indirect measure of muscle damage, despite some controversies and areas to be clarified) on the day of exercise testing, provided in table 1, are relatively low in our athletes, considering that higher CK levels/reference values are expected in athletes than in general population (see e.g. 10.1136/bjsm.2006.034041). Importantly, CK levels did not differ significantly between endurance athletes and sprinters. Therefore, we believe that we have managed to control the muscle damage factor to a sufficient extent.

As for diet and dietary supplements control, we collected such data (estimates of energy, protein, fat, and carbohydrate intake) and the new manuscript has been updated accordingly (methods, results, discussion). The limitation is that we did not obtain complete records from all athletes (7/11 in the endurance group and 6/10 in sprinters). That is why we did not include this data in the original version. However, we currently believe that even partial data can be representative and valuable for better interpretation of the results. It is most important that the studied (sub)groups did not differ significantly in the consumption of macronutrients per kg of body weight and the percentage contribution in the total energy intake. Thus, both sports groups seem to be equivalent ('standardized') in this respect, which strongly suggests that the clearly different levels and exercise-induced responses of certain plasma amino acids are due to differences in sport-specific metabolic adaptations. Overall, the percentage of energy intake from carbohydrate, protein and fat was in accordance with dietary recommendations of the Int. Soc. Sports Nutr., and the protein intake per kg body weight (2.1‒2.2 g/kg body weight) was that expected in athletes engaged in intense training (1.7‒2.2 g/kg body weight; Kersick et al. 2018, doi: 10.1186/s12970-018-0242-y). In addition, the concentration of proteinogenic plasma amino acids in our athletes did not differ significantly from the ranges shown in normally adapted high-performance athletes (Kingsbury et al. 1998, doi: 10.1136/bjsm.32.1.25). Thus, it appears that our athletes were not protein or amino acid deficient at the time of the study.

As for the day before testing, athletes were asked not to modify their dietary habits and not to hydrate extra or dehydrate before the test, not to intake supplements or aids potentially affecting amino acid levels and physical performance. To check this, they reported on their food and supplementary intake over the past 48 hours to detect unusual eating behaviors that could affect the study results (in which case laboratory measurements could be rescheduled). The energy contribution from proteins was on average within a range of 16‒17% in our athletes, thus within the typical/normal range as shown in Table 2 (Kersick et al. 2018, doi: 10.1186/s12970-018-0242-y). We agree that a standardizing to 20% could be more precise, but on the other hand, it is unclear how a sudden one-time change in dietary routine would affect the overall metabolic state in the athletes.

Lines 100-101: The authors use a vague description of the PFAA analysis, writing that they used a "well-established and validated method". It is advisable to include a detailed description of the method used.

Thank you for this comment. This section contains only main assumptions of the study design, hence it is a general description. To avoid confusion, the part of the sentence that reads “using a well-established and validated method” has been removed. The details of the analytical method with further references are described in the section “Plasma free amino acids assay”.

Statistical analysis

Lines 200-210: Can the authors provide the name of the statistical program with which they performed the statistical analyses (e.g. Statistica v13.3 PL, IBM® SPSS)?

Thank you for noticing this. All statistical analyses were performed using the Statistica 13.3 software package (TIBCO software Inc., Santa Clara, CA, USA). The information has been supplemented.

Results

The results are a very strong part of the manuscript, they were prepared with precision, contain in-depth analysis and look very good graphically.

Thank you very much for your positive evaluation. We tried to do our best.

Future research

If the authors plan further research in this area, it would be worthwhile to study the interactions between genotype and metabolome.

Influences on the metabolome are manifold and further studies are needed to disentangle the specific contributions of genetic variants, of adaptations to sports-specific exercise training or of special nutrition to the systematic metabolic differences between differently trained individuals. Genetics contribute to metabolite and protein concentration differences between subjects and can be responsible for up to 75% (for metabolites) of the metabolite concentration in blood. Especially in small and highly specialized cohorts, genetics can influence outcomes in exercise studies.

It is hard not to agree with the reviewer. A more comprehensive combined metabolomic and genomic approach could help better interpret the exercise responses, especially in the case of athletes, who in principle must have certain genetic predispositions to perform at the highest level in sports. The contribution of genetic and environmental (training) factors to exercise response, physical fitness and sports performance is still a topical issue. So far, our team has made one modest attempt in a related topic (https://doi.org/10.1007/s13353-022-00736-6).

**Reviewer #2:** The reviewer’s comments on the paper are as follows:

I have reviewed the research type article entitled “Exercise-induced response of proteinogenic and non-proteinogenic plasma free amino acids is sport-specific: A comparison of sprint and endurance athletes”.

It is interesting topic and totally, this manuscript is well organized and written. However, there are just a few points of clarification that need to be addresses. Therefore, I recommend that the manuscript must be revised before it can be reconsidered for publication. Here are my comments:

Thank you very much for your overall positive assessment. Below, we address the valuable comments. Appropriate changes were also made to the manuscript.

- Page 3, Introduction, Line 44-45: The authors wrote “Circulating blood plasma is a temporary and small but important reservoir of free AAs (~1% of total AAs)”. If plasma is temporary tank of FAAs and including one percent of total body AAs, please explain why PFAAs could have crucial role regard to exercise metabolism in particular sports specialization?

Plasma amino acid stores are temporary in the sense that their levels can change rapidly due to various factors, and in our study such a factor is exercise of increasing intensity. But, of course, the flow of this fraction of the amino acid pool in the circulatory system is continuous and ceaseless. It is well known that with exercise intensity, blood flow in skeletal muscles increases dramatically, and with it the rate of amino acid transport flow. During exercise, the circulatory system is essentially the only fast and efficient route for the exchange of amino acids between muscles and other tissues and organs. It allows the efficiency of metabolic processes to be continuously adjusted to meet the demands of physical exertion. The plasma PFAA concentration profile can be considered a good indirect indicator of amino acid turnover during exercise and the "strategy" of their utilization depending on the sports specialty. A relevant brief explanation is included in the introduction.

As an aside, it can be added that typically only the 'static' concentration of amino acids at a given exercise intensity is measured. It would be even more interesting to measure the magnitude and changes in PFAAs flow, e.g. in micromoles per liter per minute (or production and consumption of PFAAs as mentioned in the limitation section). Unfortunately, more invasive methods, not feasible for some reasons, would have to be used.

- Page 5, Line 107: Considering possible effects of diet especially day before testing, please describe more details of a 48-hour recall of participants’ dietary intake consist of total daily calorie intake, macronutrients distribution, and percent protein consumption. These data should be reported as a separate table.

Thank you for this crucial comment. As for diet and dietary supplements control, we had collected such data (estimates of energy, protein, fat, and carbohydrate intake) and the manuscript has been updated accordingly (methods, additional table in the results section, discussion). The limitation is that we did not obtain complete records from all athletes (7/10 in the endurance group and 6/10 in sprinters). That is why we didn't include this data in the original version. However, we currently believe that even partial data can be representative and valuable for better interpretation of the results. It is most important that the studied (sub)groups did not differ significantly in the consumption of macronutrients per kg of body weight and the percentage contribution in the total energy intake. Thus, both sports groups seem to be equivalent ('standardized') in this respect, which strongly suggests that the clearly different levels and exercise-induced responses of certain plasma amino acids are due to differences in sport-specific metabolic adaptations. Overall, the percentage of energy intake from carbohydrate, protein and fat was in accordance with Int. Soc. Sports Nutr. (ISSN) recommendations, and the protein intake per kg body weight (2.1‒2.2 g/kg body weight) was typical of athletes engaged in intense training (1.7‒2.2 g/kg body weight; Kersick et al. 2018, doi: 10.1186/s12970-018-0242-y). In addition, the concentration of proteinogenic plasma amino acids in our athletes did not differ significantly from the ranges shown in normally adapted high-performance athletes (Kingsbury et al. 1998, doi: 10.1136/bjsm.32.1.25). Thus, it appears that our athletes were not protein or amino acid deficient at the time of the study. The manuscript has been updated accordingly (methods, results, discussion).

By the way, the 48-hour recall of participants' dietary intake was just an additional quick check to detect abnormal 'momentary' eating behavior.

- Page 5, Exercise protocol, Line 125-126: It has been reported that total exercise duration ranged from 20 min (sprinters) to 24 min (endurance athletes). This point can be important at the observed changes in variables (Lactate, PFAAs, etc.) between two kinds of exercise metabolism and so their interpretation. Therefore, please include this issue for relevant discussion.

Thank you for raising this important question. Obviously, with an identical exercise protocol until exhaustion (same speed progression), our endurance athletes reached higher running speeds, and the test lasted longer for them. However, in the end, at the point of exhaustion, athletes from both groups presented values of physiological and biochemical indicators that objectively reflected their maximal effort/fatigue and subsequent recovery. In this regard, the groups are fully comparable (including heart rate, oxygen uptake, lactate, ammonia and all PFAAs). In this sense, the duration of the test did not affect the interpretation of our comparisons. The same applies to rest conditions, which have been standardized.

Some disparity may occur at sub-maximal exercise intensities, as, for example, at 16 km/h endurance athletes typically achieve a lower percentage of V̇O_2_max than sprinters, i.e. a lower exercise intensity in physiological terms. Also, at the same speed, sprinters may be above, whereas endurance runners below ventilatory/lactate/anaerobic threshold (not determined in this study). In our opinion, however, this does not change the main directions of our interpretation, since the main effects and interactions of the ANOVA analysis were counted globally for the entire range: from rest, through the subsequent stages of the test, to the end of recovery. Moreover, as can be seen in Figures 2, 3 and 4, the intergroup differences or their lack in the levels of PFAAs remain fairly stable over the entire range: at rest, at different intensities, and during recovery. The same is essentially true for many individual PFAAs (supplementary figures S2). In addition, an interaction effect (exercise stage*group) was shown for some of the amino acids (Thr, Trp, Gln, Gly, Pro, Ser, 1MHis, Abu, EtN, Orn, Sar, combined non-proteinogenic), i.e. the pattern of concentration changes differed between the groups. The difference generally relied on a stronger exercise response in endurance athletes, i.e. significant differences between resting and subsequent exercise concentrations were more often seen, whereas the amino acid responses in sprinters were more flattened (less frequent or smaller differences between resting and exercise concentrations). Here, too, differences in test length and relative exercise intensity were not crucial. Appropriate additions were made in the discussion.

- Please clarify why the researchers choose incremental running exercise as identical test for investigating the response of participants? What’s the reasons for? Why two tests weren’t selected fit to both sport events corresponding to specific adaptations?

Thank you for this important question. We agree that using two different sport-specific exercise tests (one for endurance, the second for speed-power abilities) would yield further interesting results. In our study, the endurance group is "favored" to some extent because the test used is more in line with the specifics of their sports discipline. The progressive test until exhaustion on a treadmill is a compromise solution in a way. A single sprint exercise bout (lasting a few or several seconds), being the target ability in our sprinters, could be to short to elicit clear PFAA changes and between-group differences. Sprinting efforts repeated multiple times could be used, however, not they are specific for any of the groups under study (more typical for team sports; and more exhaustive ‒ it would be difficult to get the consent of the athletes in the pre/competition phase). Prolonged (up to several hours) moderate-intensity exercise at or slightly above the ventilatory or lactate threshold would be an optimal option for endurance athletes, but not feasible by sprinters. So we have chosen a classic progressive test until exhaustion (universal, widely used in many sports), lasting long enough to induce changes in PFAAs concentration, but tolerated and accepted by both groups. Also, the gradual increase in running speed was very important, giving a picture of the profile of PFAA changes with exercise intensity. Most important, the test used just fulfilled its role as a standardized exercise stimulus. Even if it was not fully specific to the sports groups tested, it successfully revealed the metabolic specificity of these groups.

- Blood samples were drawn 11 times before, during and after exercise test. 5 of them were in time of doing exercise. Weren’t these sampling effective on participants’ performance? If so, please mention it as limitation.

Thank you for this interesting methodological question. As is known, every measurement involves interfering with a given system subject to measurement and changing it in some way. As far as blood volume (BV) is concerned, acute blood loss after blood donation (400‒500 mL, equivalent to ~10% BV) results in significant reductions of maximal oxygen uptake, power output, and work rate from a few to dozen or so percent (see: <https://pubmed.ncbi.nlm.nih.gov/18535983>; <https://www.ncbi.nlm.nih.gov/pmc/articles/PMC6467450/>; <https://sportsmedicine-open.springeropen.com/articles/10.1186/s40798-016-0067-7>; <https://dial.uclouvain.be/pr/boreal/object/boreal:178587>; <https://pezcyclingnews.com/toolbox/how-blood-donation-affects-performance/>). However, V̇O_2_max was not significantly changed after BV reduction by 150 mL, i.e by about 3% BV (<https://doi.org/10.1249/mss.0000000000002618>).

We did not measure BV in our athletes. However, it is known that BV is directly proportional to a person’s body weight and lean body mass, with athletes typically having higher BV at the same body weight and stronger BV-weight and BV-lean mass relationship (r^2^=0.81 and 0.91, respectively). Therefore, the BV of our athletes can be estimated based on available prediction models for general population and athletes (e.g., <https://doi.org/10.1002/ajh.27162>), and is approximately 6.3 L in endurance athletes and 7.1 L in sprinters (due to differences in lean mass). It follows that the 5 to 6 blood samples that were taken at rest and during exercise test represent a blood loss of about 12.5-15 mL. As a rough estimate, it is about 0.2% of the total BV of our athletes. In light of the available scientific data, we can conclude that such blood loss is a factor of negligibly low impact, with no significant effect on the results of our measurements, is therefore not a limitation.

At the margin, the unavoidable natural/physiological reduction in blood (plasma) volume during exercise, as well as initial resting blood volume, really affects maximal aerobic capacity and performance (see: <https://www.frontiersin.org/journals/physiology/articles/10.3389/fphys.2021.679232/full>; 10.3389/fphys.2021.679232). These factors were standardized in our study (and PFAA concentration adjusted for changes in plasma volume).

- I didn’t see an explanation about participants’ sleep. Did the researchers give advices to them? Please clarify.

Thank you for this question. Some reports demonstrate clearly that acute sleep deprivation affects metabolomic analyses (e.g. [www.pnas.org/cgi/doi/10.1073/pnas.1402663111](http://www.pnas.org/cgi/doi/10.1073/pnas.1402663111)). With this in mind, it is important for athletes to get enough sleep before testing. We have not given a direct indication of the amount of sleep. However, the risk of sleep deprivation appears negligible in our study. The participants were professional athletes, training in a structured way, and their motivation to achieve best test scores was very high (declared in pre-test questionnaire). Exercise tests were very important to them (physical capacity diagnosis, sports level assessment by sports federation, training control and planning), so they prepared as seriously as they do for competition (under the guidance of coaches). We also didn't give them some other tips (e.g., on clothing, footwear, etc.), as such issues are obvious to them. Arguably more risk in this regard should be considered if non-training/non-athletic groups are studied.

- Page 5, Line 104: The authors wrote “Athletes arrived at the laboratory in the morning after an overnight fast”. What time the test was conducted exactly? Please write.

Thank you for indicating this important detail. Athletes arrived at the laboratory at 7 a.m. and exercise test were conducted between 8 and 11 a.m. We have added this information in the Methods section.

- Page 7, Other blood assays, Line 193-197: Please mention sensitivity or CV of measurements for blood lactate, ammonia, and creatine kinase.

Thank you for this important comment. The information has been supplemented in this section.

- Page 8, Table 1: Fat mass percent of endurance and sprint athletes were 13.7 vs. 10.7 respectively. Are those correct? Please just recheck.

Thank you for this question. In fact, training in many sports disciplines, especially those demanding highest levels of speed-power or endurance (or a mix) require and cause strong fat reduction. Our percentage fat mass values are correct, measured using the reliable DXA method. High-performance athletes at national and international level, in particular sprinters, are characterized by very low fat content in the pre-competition and competition training phases (compared to the general population). Therefore, these are values typical of competitive athletes in such sports disciplines, repeatedly observed in our laboratory on the occasion of other research projects, also mentioned as a standard in textbooks (e.g., American Council on Exercise, 2003, ACE Personal Trainer Manual, 3rd Ed., Ch. 6, p. 188, Table 6.14; Jeukendrup A, Gleeson M, 2019, Sport nutrition. Human Kinetics, Champaign, IL (3rd edition).

- Page 15, Implications, Line 429-455: It’s a bit long. Please summarize content without repeating the literature. Moreover, considering a lot of limitations in this study rewrite some sentences (practical messages) with possibility and uncertainty.

Thank you for this suggestion. We agree that the reader may find this part too long. We tried to make the text more concise, but the cited literature items are not repetitions, so we left them (except for two).

- Page 17, Conclusions, Line 475-481: This sub-section need to be revised on basis of previous comment. Caution should be considered when writing the concluding sentences in particular the first sentences.

Thank you for this critical comment. W agree that our conclusions should be expressed with caution, because this is the first study with such a design and needs to be verified in other studies. We have softened the key sentences of the conclusions to make them less authoritative/categorical.

Regards,

**Reviewer #3:** The publication titled “Exercise-induced response of proteinogenic and non-proteinogenic plasma free amino acids is sport-specific: A comparison of sprint and endurance athletes” is a significant study examining how exercise affect the levels of proteinogenic and non-proteinogenic plasma free amino acids. I recommend that the editor to accept this manuscript after a minor revision addressing the following concerns:

1. The supporting information is missing from the original file. The author should upload the supporting information when submitting the revision.

Thank you for this important technical remark. Files containing supporting information were correctly uploaded by us during submission. In the pdf file we have, one can access these materials by clicking on a special link on separate pages (e.g., Click here to access/download Supporting Information 0-S1TA~1.PDF). Perhaps there is some technical problem beyond the authors' control.

2. The author should consider arranging the figures horizontally instead of vertically.

Thank you for bringing it to our attention. The figures were uploaded to the system as separate files in eps format. In the file made available to us, the figures in the main text are presented on horizontally oriented pages, while figures in the supporting information are vertically oriented. However, these are working materials for now, and the question of how to present the figures is open. On the one hand, it is worth considering the convenience of the reader (turning the pages 90 degrees while reading may be uncomfortable), and on the other hand, the publisher has certain requirements and standards. We think that an optimal solution will be found.

***
