# Supplementary material for: Exercise-induced response of proteinogenic and non-proteinogenic plasma free amino acids is sport-specific: A comparison of sprint and endurance athletes
Source: PLoS One. 2024 Aug 30;19(8):e0309529. doi: 10.1371/journal.pone.0309529 (PMC11364291; doi:10.1371/journal.pone.0309529)
Supplement: Attachment — Submitted filename: Review 2.docx [file pone.0309529.s007.docx]

The authors have addressed all the concerns. I recommend the editor except this manuscript without further revision.
